# Pregnancy complications and risk of cardiometabolic-renal conditions in 1.4 million women in England: longitudinal study

**DOI:** 10.1136/bmjmed-2026-003035

**Published:** 2026-10-06

**Authors:** Steven Wambua, Megha Singh, Mohamed Rishard, Nadia Sarween, Pushpa Singh, Christopher Yau, Richard D Riley, Nicola J Adderley, Kym I E Snell, Francesca L Crowe, Krishnarajah Nirantharakumar

**Affiliations:** 1 King’s College London School of Life Course and Population Sciences, London, UK; 2 Department of Applied Health Sciences, College of Medicine and Health, University of Birmingham, Birmingham, UK; 3 Faculty of Medicine, University of Colombo, Colombo, Sri Lanka; 4 Li Ka Shing Centre for Health Information and Discovery, University of Oxford Big Data Institute, Oxford, UK; 5 Nuffield Department of Women’s and Reproductive Health, John Radcliffe Hospital, University of Oxford, Oxford, UK; 6 Health Data Research UK, London, UK; 7 National Institute for Health and Care Research (NIHR), NIHR Birmingham Biomedical Research Centre, Birmingham, UK

**Keywords:** Pregnancy complications, Cardiology, Hypertension, Diabetes mellitus

## Abstract

**Objective:**

To investigate the association between pregnancy complications and cardiometabolic-renal conditions (ie, cardiovascular disease, type 2 diabetes mellitus, hypertension, and chronic kidney disease) in the subsequent years after pregnancy.

**Design:**

Longitudinal study.

**Setting:**

Clinical Practice Research Datalink (CPRD) Aurum primary care database pregnancy register, UK, 1 January 2000 to 31 December 2022.

**Participants:**

1 432 795 women, aged 15-49 years, who had been pregnant, with or without pregnancy complications, and registered with a general practitioner between 1 January 2000 and 31 December 2022.

**Main outcome measures:**

First recorded diagnosis of each of the four conditions: cardiovascular disease (ischaemic heart disease or cerebrovascular disease, including stroke and transient ischaemic attack), type 2 diabetes mellitus, hypertension, and chronic kidney disease (stages 3-5).

**Results:**

1 422 287 participants were included in the cardiovascular disease outcome analysis, 1 389 460 in the hypertension, 1 411 883 in the type 2 diabetes mellitus, and 1 430 965 in the chronic kidney disease cohorts. Median follow-up was 4.1-4.3 years across the four cohorts (interquartile range approximately 1.5-9.8). Of 72 associations examined, 51 were significant after correction for multiple testing. Well established associations were found. Gestational hypertension and pre-eclampsia more than tripled the rate of hypertension and approximately doubled the rate of chronic kidney disease, with smaller increases for cardiovascular disease and type 2 diabetes mellitus. Gestational diabetes mellitus was associated with a sevenfold higher rate of type 2 diabetes mellitus and increased rates for hypertension and cardiovascular disease, but not for chronic kidney disease. Less well established associations were also found. Postnatal depression increased the rate of all four outcomes by 35-56% for the latest pregnancy (hypertension hazard ratio 1.36, 95% confidence interval (CI) 1.33 to 1.40; type 2 diabetes mellitus hazard ratio 1.41, 1.36 to 1.46; cardiovascular disease hazard ratio 1.56, 1.50 to 1.62; and chronic kidney disease hazard ratio 1.35, 1.25 to 1.45). Miscarriage, stillbirth, placental abruption, and preterm birth in the latest or in a previous pregnancy were associated with modestly higher rates of one or more outcomes (eg, stillbirth in the latest pregnancy: type 2 diabetes mellitus hazard ratio 1.67, 95% CI 1.33 to 2.08 and chronic kidney disease hazard ratio 1.79, 1.19-2.70; placental abruption in the latest pregnancy: chronic kidney disease hazard ratio 1.67, 1.06 to 2.62). Small-for-gestational-age birth was associated with higher rates of hypertension and cardiovascular disease, and large-for-gestational-age birth with higher rates for type 2 diabetes mellitus, including without gestational diabetes mellitus.

**Conclusions:**

Several pregnancy complications, including those occurring in previous pregnancies, were associated with an increased risk of cardiometabolic-renal conditions in the subsequent years after pregnancy. These findings support obtaining a full reproductive history and using the postpartum period as an opportune time for risk assessment and preventive cardioprotective strategies in women with a history of adverse pregnancy outcomes.

**WHAT IS ALREADY KNOWN ON THIS TOPIC**

- The association between gestational diabetes mellitus and hypertensive disorders of pregnancy, and hypertension, type 2 diabetes, cardiovascular disease, and chronic kidney disease has been established in systematic reviews and umbrella reviews
- For most other pregnancy complications, such as miscarriage, stillbirth, placental abruption, preterm birth, small and large for gestational age, and postnatal depression, limited evidence exists from systematic reviews
- Most studies considered only the most recent pregnancy, few examined chronic kidney disease, and recent reviews suggest that studies often adjusted for only a limited or inconsistent set of risk factors

**WHAT THIS STUDY ADDS**

- Ten pregnancy complications were evaluated, including gestational diabetes mellitus and hypertensive disorders of pregnancy in the most recent and previous pregnancies, in relation to four cardiometabolic-renal conditions while adjusting for a comprehensive and consistent set of established risk factors in a large primary care database
- As well as gestational diabetes mellitus and hypertensive disorders of pregnancy, postnatal depression was associated with all four cardiometabolic-renal conditions
- Preterm birth, miscarriage, stillbirth, placental abruption, and small and large for gestational age were associated with at least one cardiometabolic-renal condition

**HOW THIS STUDY MIGHT AFFECT RESEARCH, PRACTICE, OR POLICY**

- Postpartum cardiometabolic-renal risk assessment is typically prompted by the most recent pregnancy, but these findings suggest that complications in earlier pregnancies may also provide relevant risk information
- Risk assessment may need to extend beyond gestational diabetes mellitus and hypertensive disorders of pregnancy to include other pregnancy complications
- Further research should test whether cardiometabolic-renal risk prediction models that incorporate pregnancy history improve risk estimation in women who have been pregnant

## Introduction

Cardiometabolic-renal conditions, including cardiovascular disease, diabetes, hypertension, and chronic kidney disease, are leading causes of morbidity and mortality globally.^1–4^ For instance, cardiovascular disease is the leading cause of death in women, responsible for nearly a third of deaths in women aged <55 years, with increasing incidence in recent years.^5–7^ Although men tend to develop clinical disease 10-15 years earlier than women, outcomes are thought to be worse for women because of delayed diagnosis, atypical presentation, and lack of recognition of sex specific risk factors, such as a history of hypertensive disorders of pregnancy and gestational diabetes, which are not considered comparable with more traditional cardiovascular risk factors, such as smoking and obesity.^8–10^ Furthermore, women from ethnic minority groups and those from lower income backgrounds often encounter even greater inequalities in both the incidence and outcomes from cardiometabolic disease because of inequalities affecting access to care, prevention, early diagnostic tools, and effective treatments.^11–14^ The under-representation of women in clinical trials, for example in stroke trials, has also hindered the development of sex sensitive medical interventions, reinforcing these health inequalities.^15 16^

Pregnancy is a unique physiological state and can affect longer term health outcomes. Some adverse pregnancy outcomes, such as hypertensive disorders of pregnancy and gestational diabetes mellitus, increase a woman's lifetime risk of developing cardiometabolic-renal conditions that include ischaemic heart disease, stroke, hypertension, type 2 diabetes mellitus, and chronic kidney disease.^1–3 9 10^ Better understanding and identification of risk factors for cardiometabolic-renal conditions in women is needed to facilitate early preventive interventions. In particular, the postpartum period has been identified as an opportune time to start preventive measures to reduce the risk of cardiovascular disease in this population.^17^ The American Heart Association has recommended that a woman's reproductive history should be recorded routinely to help assess her cardiovascular risk when screening for targeted interventions.^18^ Although previous studies have assessed the association between pregnancy complications and cardiometabolic-renal conditions, many had important limitations. In particular, several studies adjusted for only a limited or inconsistent set of risk factors, so that establishing whether pregnancy complications added prognostic information beyond the established risk factors routinely available to clinicians was unclear. Some studies examined associations for just one pregnancy and only a few studies assessed outcomes from previous pregnancies as well as the most recent pregnancy. Also, most studies focused largely on gestational diabetes mellitus and hypertensive disorders of pregnancy, such as pre-eclampsia.^10 17 19^ Therefore, our main objective was to examine the association between a spectrum of pregnancy complications from multiple pregnancies and the risk of developing cardiovascular disease, type 2 diabetes mellitus, hypertension, and chronic kidney disease post partum.

## Methods

### Database and study population

We used the Clinical Practice Research Datalink (CPRD) Aurum primary care database pregnancy register to select the target population of women aged 15-49 years who had been pregnant and were registered with a general practitioner between 1 January 2000 and 31 December 2022, with linkage to Hospital Episodes Statistics.^9 10 20^ Participants were pregnant women and other pregnant people. To ensure that we had sufficient quality data at baseline, participants contributed to the cohort after a minimum registration period with their general practice of at least a year. Women were followed up from 15 months after the date of conception of the latest pregnancy (about six months post partum), the index date, to allow for normal physiological changes of pregnancy to resolve and allow time for postpartum information to be recorded in the primary care database.^21 22^ The latest pregnancy was chosen as the index because, in practice, postpartum risk assessment occurs when a woman is seen after her most recent pregnancy, with her full reproductive history available. Therefore, fixing the index date to this point reflects the clinical scenario where these findings would be applied. Pregnancies occurring before 1 January 2000 were not used to define cohort entry, but historical information obtained before 2000, when available, was used to record baseline characteristics and previous pregnancy complications. The study end date was 31 December 2022, and women were censored at the earliest of when the outcome occurred, transfer to another practice, the last data collection date, death, or 31 December 2022. Figure 1 summarises the study design.

**Figure 1:**
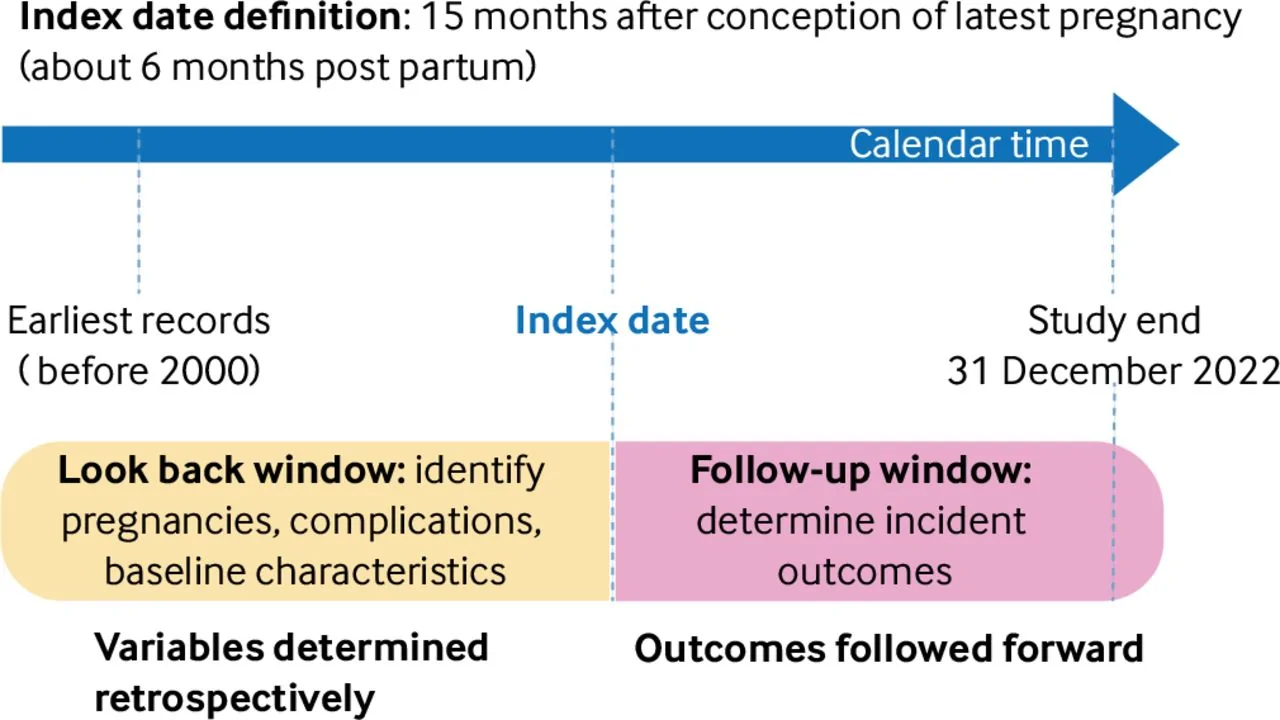
Study design

For each outcome, women were eligible only if they had no record of the outcome of interest at any time up to and including the index date, ensuring that the outcomes analysed were incident events that occurred during follow-up. Hence women with cardiovascular disease were excluded from the cardiovascular disease outcome analysis, those with pre-existing diabetes (type 1 or type 2) were excluded from the type 2 diabetes mellitus outcome analysis, those with chronic hypertension were excluded from the hypertension outcome analysis, and those with chronic kidney disease were excluded from the chronic kidney disease outcome analysis.

### Pregnancy complications and covariates at baseline

Ten pregnancy complications were identified from the literature and from discussions with clinicians and patient research partners as part of a national consortium investigating the relation between long term conditions and pregnancy complications (MuM-PreDiCT: Multimorbidity in Pregnancy: Determinants, Clusters, Consequences and Trajectories).^20^ These complications included the well established complications of gestational hypertension, pre-eclampsia, and gestational diabetes mellitus, as well as the less well established complications of miscarriage, stillbirth, placental abruption, preterm birth, small and large for gestational age, and postnatal depression. History status was categorised into three groups: history of the pregnancy complication in the last or latest pregnancy (eg, miscarriage in the latest pregnancy), history of the pregnancy complication in a previous pregnancy (eg, miscarriage in a previous pregnancy), and no history of the pregnancy complication (eg, no history of miscarriage).

Covariates were all of the traditional risk factors of the four cardiometabolic-renal conditions identified from the literature.^23–27^ Covariates were selected to support prognostic risk estimation rather than causal inference. Selection was informed by established cardiometabolic risk prediction models (including the QRISK3 and QDiabetes algorithms), previous epidemiological literature, and clinical expertise. Directed acyclic graphs were used to represent the relations between pregnancy complications, established risk factors, and cardiometabolic-renal outcomes, and to guide the selection of variables for adjustment. The directed acyclic graph for the cardiovascular disease analysis (online supplemental figure S1a) was developed by the authors using DAGitty, with the selection of some variables and causal relationships informed by a previous study of pregnancy complications and cardiovascular disease.^27^ The directed acyclic graphs for the remaining outcomes (online supplemental figure S1b–d) were developed using the same approach, informed by relevant literature and hypothesised relationships between variables. The directed acyclic graphs were used to identify established risk factors to condition on, so that the adjusted hazard ratios reflected the prognostic contribution of a complication after accounting for those risk factors. These risk factors were age, ethnic group, deprivation, systolic blood pressure (except for the hypertension outcome), body mass index, smoking status, alcohol misuse, family history of cardiovascular disease, diabetes mellitus (type 1 and type 2), venous thromboembolism, migraine, rheumatoid arthritis, systemic lupus erythematosus, severe mental illness, atrial fibrillation, learning disability, treatment for hypertension (except for the hypertension outcome), corticosteroid prescription, statins prescription, and atypical antipsychotic drug treatments.

10.1136/bmjmed-2026-003035.supp1 Supplementary data

Deprivation was measured with the index of multiple deprivation, an area level composite measure of relative deprivation in England, based on residential postcode. The index of multiple deprivation combines information across multiple domains, including income, employment, education, health, crime, housing, and living environment, and is widely used in UK epidemiological research. The index of multiple deprivation scores were categorised into five equal national groups, with group 1 representing the most deprived areas and group 5 the least deprived. If data were missing for the index of multiple deprivation, a separate missing category was retained in the analyses.

Because pre-eclampsia is closely linked to several complications, such as placental abruption, preterm birth, stillbirth, and small for gestational age, pre-eclampsia was considered a potential confounder and was adjusted in the models for these complications, similar to other studies.^27^ This approach aligns with previous epidemiological studies and helps reduce confounding due to underlying vascular or metabolic dysfunction. We did not try to differentiate between the proportion of risk attributable to one complication versus another, however, because this analysis is not feasible without strong causal assumptions that cannot be justified with observational data. Because several pregnancy complications may share underlying biological pathways or cluster within individuals, we did not mutually adjust all pregnancy complications for one another to avoid over-adjustment and collider bias. Instead, models were adjusted for established cardiometabolic-renal risk factors that were clinically relevant and routinely available in primary care.

### Outcome

The outcome of this study was the first recorded diagnosis of each of the four conditions: cardiovascular disease (ischaemic heart disease and cerebrovascular disease (stroke and transient ischaemic attack)), type 2 diabetes mellitus, hypertension, and chronic kidney disease (stages 3-5).

### Determining outcomes and variables

The variables, covariates, and outcomes were determined with SNOMED CT (Systematised Nomenclature of Medicine-Clinical Terms).^28^ As well as the clinical codes, chronic kidney disease was determined from at least two values for estimated glomerular filtration rate <60 mL/min/1.73 m^2^, more than 90 days apart.^29^ Online supplemental table S1 lists the definitions of pregnancy complications, covariates, and outcomes used in the analyses.

### Statistical analysis

We created four cohorts of eligible women for the analysis of each outcome (cardiovascular disease, type 2 diabetes mellitus, hypertension, and chronic kidney disease). These cohorts were not distinct but differed because of outcome specific exclusion criteria. Each of the pregnancy complications was studied in relation to each outcome.

Categorical variables are presented as counts and percentages and continuous variables as median (interquartile range) or mean (standard deviation), depending on the distribution. We used the cumulative hazard plots with the Aalen-Johansen estimator to visualise and compare the cumulative hazard over time in women with a complication in the latest pregnancy, in a previous pregnancy, or with no history of the pregnancy complication. Median follow-up time and 95% confidence intervals (CIs) were estimated with the reverse Kaplan-Meier method to account for censoring. Incidence rates were also calculated as the number of events divided by the person time at risk, and are reported per 1000 person years.

The association between pregnancy complications and cardiometabolic-renal outcomes in the postpartum period was assessed with Cox proportional hazards regression models accounting for general practice level clustering by using robust standard errors, because women attending the same general practice were likely to receive comparable care, similar to other studies.^30^ For each association, the models were used to estimate hazard ratios with 95% CIs and are presented as forest plots. Both univariable and multivariable Cox proportional hazards models were fitted for each association and multiple testing was accounted for with the Benjamini-Hochberg method of false discovery rate.^31^

Because pregnancy complications may coexist and may have shared causal pathways (eg, gestational diabetes mellitus and stillbirth), we treated each complication as a distinct clinical risk marker rather than a mutually exclusive causal variable. The objective of the study was prognostic rather than causal. We reported two complementary measures: unadjusted associations, which describe how the presence of a specific pregnancy complication in the latest or any previous pregnancy might determine a future cardiometabolic-renal risk, and are the most directly interpretable for simple risk stratification; and multivariable adjusted associations, which describe whether a complication in the latest or any previous pregnancy has prognostic information beyond established risk factors already available to clinicians (such as age, body mass index, smoking, and existing comorbidity). Neither is intended to estimate the causal effect of an individual complication. Accordingly, hazard ratios should be interpreted as reflecting the relative risk associated with having a history of that complication compared with no history, rather than as causal effects or as additive across the pregnancy complications.

### Sensitivity analyses

We carried out sensitivity analyses to assess the proportional hazards assumption violation with log-log survival plots. When the assumption was violated, we carried out a sensitivity analysis by refitting the regression model to include an interaction term with time, to explore the effect of the assumption violation on the findings. To assess whether our findings were sensitive to the choice of index pregnancy, we repeated the primary analysis with two alternative index definitions: the first recorded pregnancy and a randomly selected pregnancy for each participant.

### Missing data

A missing entry for a covariate (eg, migraine) was taken to indicate the absence of the condition (eg, no history of migraine). Missing data in continuous variables (body mass index and systolic blood pressure) were imputed with multiple imputation with chained equations, based on 15 imputations, which equals the percentage of incomplete cases, as recommended in previous literature.^32^ Specifically, multiple imputation by chained equations was performed in R with the MICE package, generating 15 imputed datasets. The imputation model included all variables used in the primary analysis for each outcome. To account for the time-to-event nature of the analysis, we included the Nelson-Aalen estimate of the cumulative baseline hazard (derived from the nelsonaalen() function from the survival package) and the event indicator, in accordance with the recommendations.^32 33^ The imputation procedure used fully conditional specification with predictive mean matching (only body mass index and systolic blood pressure were imputed). Diagnostics were performed with summary statistics and density plots to compare the distributions of the observed and imputed values. All analyses were conducted across the imputed datasets with Rubin's rules to pool estimates. If data for smoking status, index of multiple deprivation, or ethnic group were missing, a missing category was used. All analyses were performed in R version 4.4.1.

### Patient and public involvement

Patient and public involvement was extensive, from design to dissemination across the research outputs of the MuM-PreDiCT consortium. Our patient and public involvement representatives advised on the importance and relevance of this study and helped shape the research question. The representatives were also involved in identifying and prioritising the pregnancy complications examined in this study. Patient and public involvement representatives will contribute to the interpretation and dissemination of the findings to patients, the public, and healthcare professionals.

## Results

### Study population characteristics

Figure 2 shows the selection of the study population, with details of exclusions and final numbers in each of the four cohorts. The number of eligible women was 1 432 795. After excluding women with each of the outcomes at the index date, 1 422 287 participants were included in the cardiovascular disease outcome analysis, 1 389 460 in the hypertension, 1 411 883 in the type 2 diabetes mellitus, and 1 430 965 in the chronic kidney disease analyses. Table 1 shows the baseline characteristics for each cohort. Mean age across the cohorts was 33.1 (standard deviation 6.6) years and median body mass index was 24.6 (interquartile range 21.8-28.8). Most participants were from the white ethnic group, were non-smokers, and were from the most deprived areas. The most common comorbidity was migraine (11.0%). The most frequently diagnosed outcome during follow-up was hypertension (3.5%) followed by type 2 diabetes mellitus (1.5%), cardiovascular disease (1.1%), and chronic kidney disease (0.4%).

**Figure 2:**
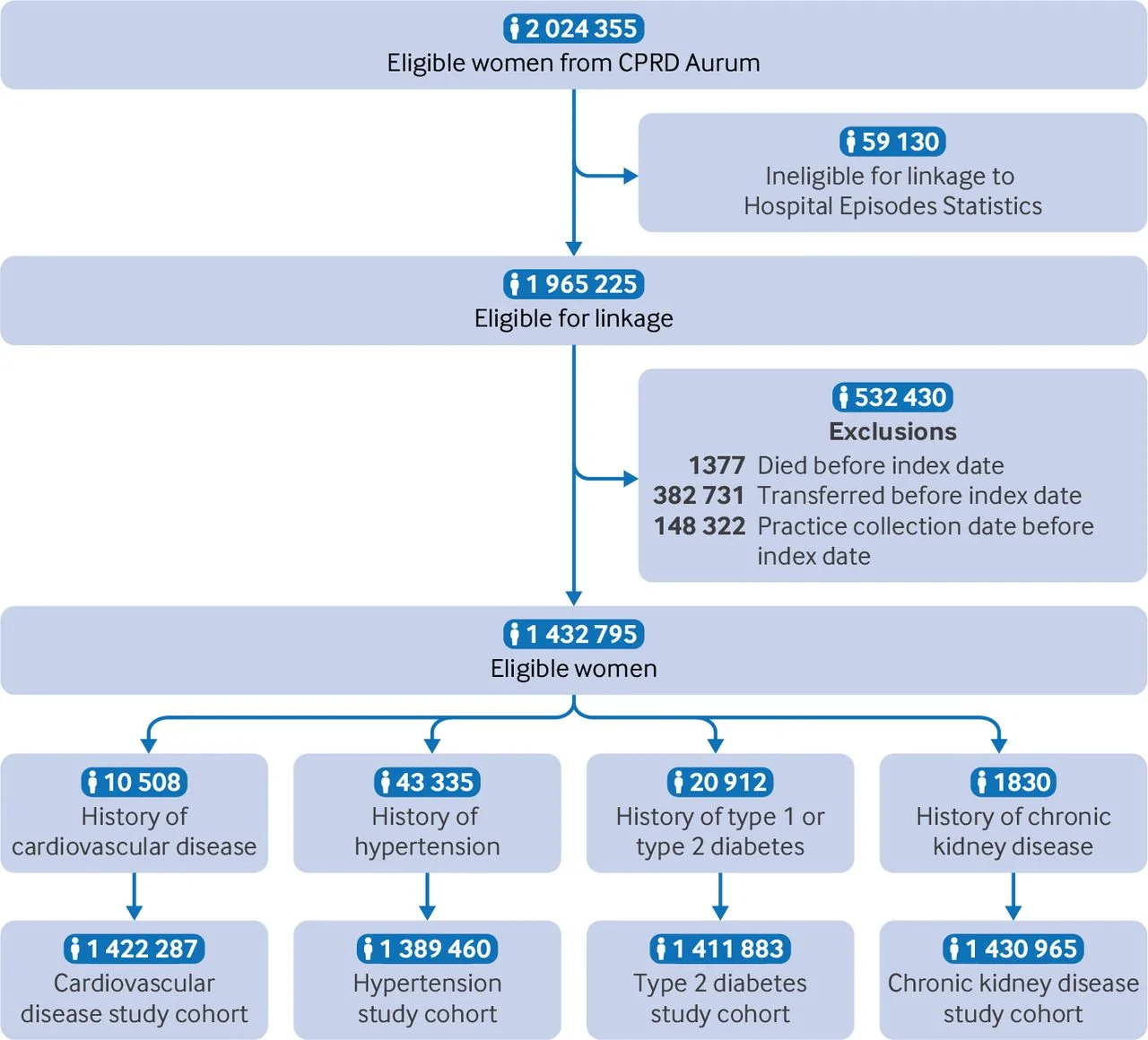
Flowchart of selection of patients for the four study cohorts

**Table 1:** Baseline characteristics of the four cohorts

Characteristics	Cohort			
	Hypertension (n=1 389 460)	Type 2 diabetes mellitus (n=1 411 883)	Cardiovascular disease (n=1 422 287)	Chronic kidney disease (n=1 430 965)
Mean (SD) age (years)	33.1 (6.6)	33.2 (6.6)	33.2 (6.6)	33.2 (6.6)
Index of multiple deprivation group:				
1 (most deprived)	303 343 (21.8)	308 870 (21.9)	312 517 (22.0)	314 934 (22.0)
2	296 038 (21.3)	301 154 (21.3)	303 692 (21.4)	306 119 (21.4)
3	262 103 (18.9)	266 343 (18.9)	267 998 (18.8)	269 563 (18.8)
4	260 797 (18.8)	264 681 (18.8)	266 046 (18.7)	267 318 (18.7)
5 (least deprived)	264 925 (19.1)	268 549 (19.0)	269 740 (19.0)	270 728 (18.9)
Missing index of multiple deprivation	2254 (0.2)	2286 (0.2)	2294 (0.2)	2303 (0.2)
Median (IQR) body mass index	24.60 (21.80-28.84)	24.70 (21.80-28.91)	24.75 (21.88-29.00)	24.77 (21.90-29.00)
Missing body mass index	203 896 (14.7)	206 804 (14.6)	205 156 (14.4)	207 361 (14.5)
Median (IQR) systolic blood pressure	116 (108-123)	116 (109-124)	116 (109-124)	116 (109-124)
Missing systolic blood pressure	41 126 (3.0)	41 290 (2.9)	41 291 (2.9)	41 381 (2.9)
Ethnic group:				
White	861 860 (62.0)	875 746 (62.0)	880 977 (61.9)	886 212 (61.9)
Black	63 140 (4.5)	66 816 (4.7)	68 224 (4.8)	68 646 (4.8)
Asian	116 541 (8.4)	116 652 (8.3)	120 121 (8.4)	120 706 (8.4)
Mixed	20 917 (1.5)	21 366 (1.5)	21 643 (1.5)	21 744 (1.5)
Other	16 274 (1.2)	16 467 (1.2)	16 627 (1.2)	16 709 (1.2)
Missing ethnic group	310 728 (22.4)	314 836 (22.3)	314 695 (22.1)	316 948 (22.1)
Smoking status:				
Non-smoker	744 341 (53.6)	757 057 (53.6)	764 394 (53.7)	768 411 (53.7)
Smoker	320 972 (23.1)	325 387 (23.0)	326 931 (23.0)	329 722 (23.0)
Ex-smoker	226 423 (16.3)	230 315 (16.3)	231 594 (16.3)	233 180 (16.3)
Missing smoking	97 724 (7.0)	99 124 (7.0)	99 368 (7.0)	99 652 (7.0)
Alcohol misuse	24 616 (1.8)	25 473 (1.8)	25 852 (1.8)	26 184 (1.8)
Family history of cardiovascular disease	95 919 (6.9)	99 285 (7.0)	100 398 (7.1)	101 514 (7.1)
Median (IQR) gravidity	3 (2-4)	3 (2-4)	3 (2-4)	3 (2-4)
Gravidity categories:				
1	309 970 (22.3)	313 387 (22.2)	315 369 (22.2)	316 977 (22.2)
2	332 221 (23.9)	336 912 (23.9)	338 494 (23.8)	340 357 (23.8)
3	268 723 (19.3)	273 236 (19.4)	274 887 (19.3)	276 538 (19.3)
4	185 209 (13.3)	188 594 (13.4)	190 056 (13.4)	191 196 (13.4)
≥5	293 337 (21.1)	299 754 (21.2)	303 481 (21.3)	305 897 (21.4)
Drug treatments:				
Statins	2040 (0.1)	1682 (0.1)	3358 (0.2)	3905 (0.3)
Treated hypertension	—	9476 (0.7)	10 929 (0.8)	11 387 (0.8)
Corticosteroids	3904 (0.3)	4222 (0.3)	4323 (0.3)	4369 (0.3)
Antipsychotic agents	4621 (0.3)	4751 (0.3)	4955 (0.3)	5059 (0.4)
Health conditions:				
Type 1 diabetes mellitus	5434 (0.4)		6387 (0.4)	6382 (0.4)
Venous thromboembolism	9312 (0.7)	9665 (0.7)	9618 (0.7)	9935 (0.7)
Migraine	150 537 (10.8)	154 540 (11.1)	155 248 (11.0)	157 002 (11.1)
Rheumatoid arthritis	4828 (0.3)	5038 (0.4)	5182 (0.4)	5265 (0.4)
Systemic lupus erythematosus	2103 (0.2)	2362 (0.2)	2340 (0.2)	2373 (0.2)
Severe mental illness	10 874 (0.8)	11 111 (0.8)	11 407 (0.8)	11 568 (0.8)
Atrial fibrillation	1174 (0.1)	1414 (0.1)	1313 (0.1)	1492 (0.1)
Learning disability	4351 (0.3)	4457 (0.3)	4578 (0.3)	4634 (0.3)
Hypertension		35 470 (2.5)	38 554 (2.7)	39 489 (2.8)
Type 2 diabetes mellitus	11 568 (0.8)		15 690 (1.1)	16 145 (1.1)
Cardiovascular disease	8396 (0.6)	9695 (0.7)		10 353 (0.7)
Chronic kidney disease	814 (0.1)	1468 (0.1)	1675 (0.1)	
Events:				
Outcomes	49 233 (3.5)	21 754 (1.5)	16 069 (1.1)	5288 (0.4)
Median (IQR) follow-up (years)	4.11 (1.46-9.36)	4.21 (1.49-9.65)	4.24 (1.49-9.69)	4.25 (1.49-9.75)
Median (95% CI) follow-up (years)*	4.24 (4.23 to 4.25)	4.26 (4.25 to 4.28)	4.27 (4.26 to 4.29)	4.27 (4.25 to 4.28)

Data are number (%) unless indicated otherwise.

*Based on reverse Kaplan-Meier method.

CI, confidence interval; IQR, interquartile range; SD, standard deviation.

Online supplemental tables S2–S5 show the baseline characteristics of participants in each cohort by history status (no history of the pregnancy complication, pregnancy complication in the latest pregnancy, and previous history of the pregnancy complication). In the hypertension cohort, women were of similar ages across these groups for pregnancy complications, except for miscarriage (no history of miscarriage, age 33 years; miscarriage in the latest pregnancy, 36 years; and previous miscarriage, 35 years), gestational diabetes mellitus (no history of gestational diabetes mellitus, age 33 years; gestational diabetes mellitus in the latest pregnancy, 34 years; and previous gestational diabetes mellitus, 36 years), and stillbirth (no history of stillbirth, age 33 years; stillbirth in the latest pregnancy, 32 years; and previous stillbirth, 35 years) where women with the pregnancy complications in the latest pregnancy or previous history of these complications were generally older. Women with gestational diabetes mellitus or with babies born small for gestational age in the latest pregnancy or with a previous history of the complications were more likely to be from the Asian ethnic group (15.1-21.8%) than those without a history of the pregnancy complications (7.7-8.1%). Body mass index was higher in women with pregnancy complications in the latest pregnancy or with a previous history of the complications than in those without a history of pregnancy complications, except for placental abruption, preterm birth, and small for gestational age. Women with postnatal depression in the latest pregnancy were more likely to have alcohol misuse (4.5-4.6%) than those with no history (1.3%) and those with previous postnatal depression (2.9-3.0%).

### Risk of hypertension

During a median follow-up of four years, 49 233 (3.5%) women developed hypertension. The relatively short median follow-up period, despite a study period of more than two decades, reflects the open cohort design of the CPRD Aurum where each woman contributed follow-up data from her individual index date. Figure 3 shows the cumulative incidence of hypertension in women with no history of the pregnancy complications, with pregnancy complications in the latest pregnancy, and with a previous history of the pregnancy complications. Incident hypertension rates were higher overall in women with pregnancy complications in the latest pregnancy or with a previous history of pregnancy complications than in those without a history of the pregnancy complications. Women with gestational hypertension, pre-eclampsia, or gestational diabetes mellitus in the latest pregnancy had higher rates than women with other pregnancy complications. Online supplemental table S6 shows the incidence rates of hypertension by history of pregnancy complications.

**Figure 3:**
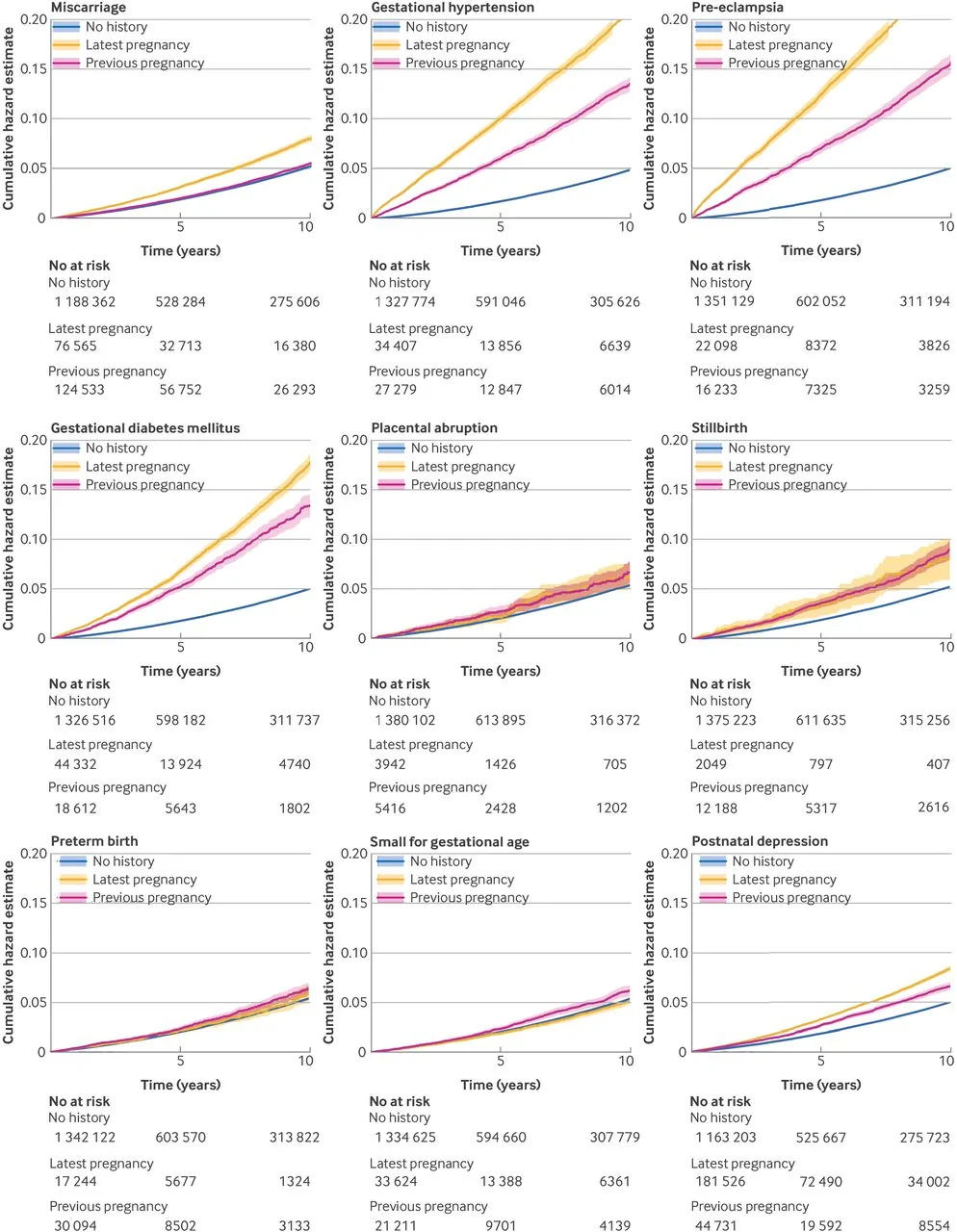
Cumulative hazard plots for hypertension in women with no history of the pregnancy complications, with pregnancy complications in the latest pregnancy, and with a previous history of the pregnancy complications

Figure 4 shows the hazard ratios for the association between pregnancy complications and hypertension, adjusting for traditional risk factors. Online supplemental table S7 provides a list of adjustment factors for each association.

**Figure 4:**
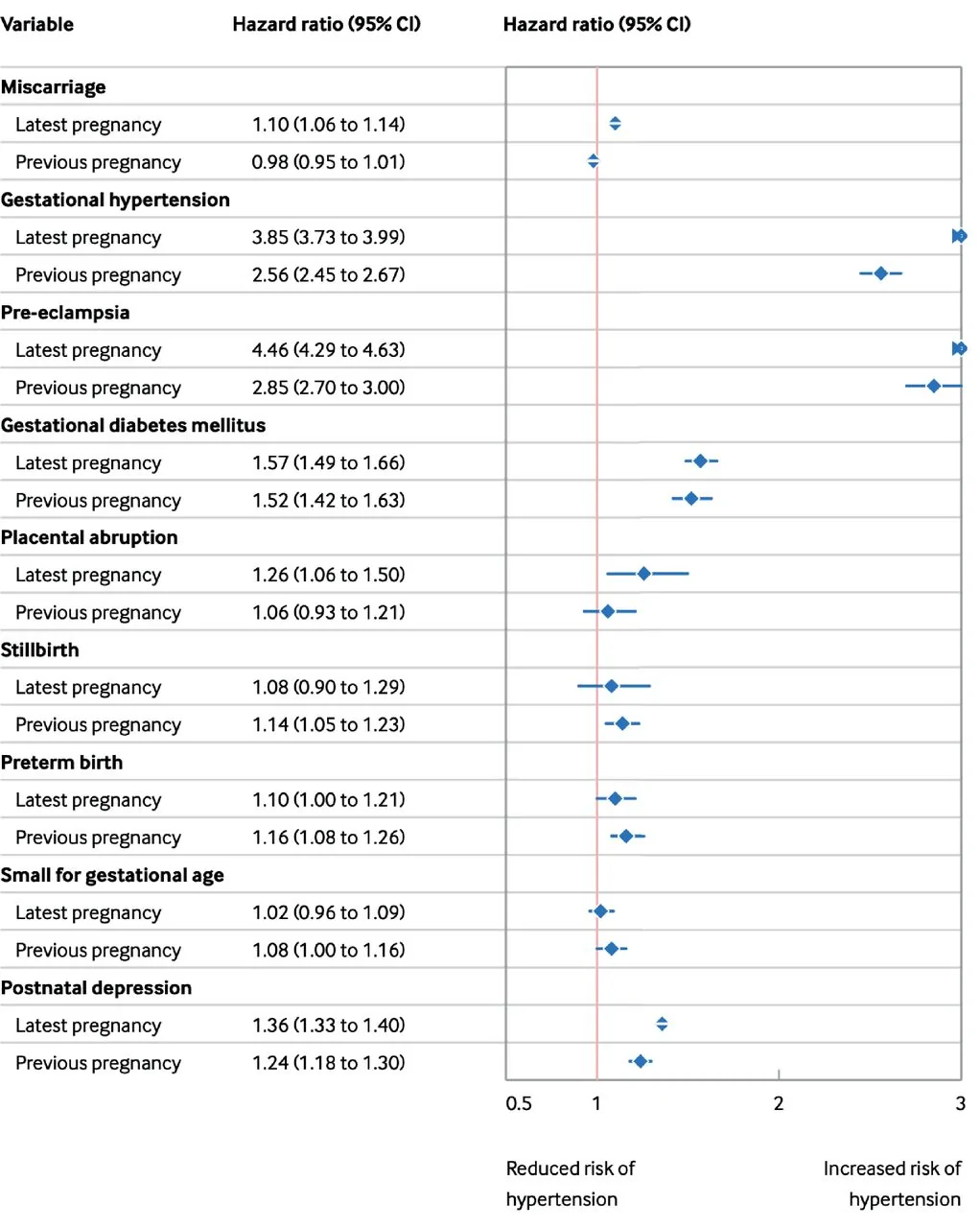
Adjusted hazard ratios for the association between pregnancy complications and hypertension in women with pregnancy complications in the latest pregnancy and with a previous history of the pregnancy complications, in comparison with women without a history of the complications

#### Well established associations

The rate of hypertension in women with gestational hypertension or pre-eclampsia in the latest pregnancy was tripled (gestational hypertension hazard ratio 3.85, 95% CI 3.73 to 3.99; pre-eclampsia hazard ratio 4.46, 4.29 to 4.63) and was doubled in women with a previous history of these complications (gestational hypertension hazard ratio 2.56, 2.45 to 2.67; pre-eclampsia hazard ratio 2.85, 2.70 to 3.00) compared with women without a history of the complications (figure 4). Women with gestational diabetes mellitus in the latest or in a previous pregnancy were also more likely to develop hypertension, with those with the complication in the latest pregnancy at even higher risk (gestational diabetes mellitus in the latest pregnancy, hazard ratio 1.57, 1.49 to 1.66; gestational diabetes mellitus in a previous pregnancy, hazard ratio 1.52, 1.42 to 1.63).

#### Less well established associations

Women with postnatal depression in the latest pregnancy or with a previous history of the complication were more likely to develop hypertension (postnatal depression in the latest pregnancy, hazard ratio 1.36, 95% CI 1.33 to 1.40; postnatal depression in a previous pregnancy, hazard ratio 1.24, 1.18 to 1.30) (figure 4). Although the rate of hypertension in women with stillbirth, preterm birth, or small for gestational age in the latest or in a previous pregnancy was higher than those without a history of the complication, the rate was similar in those with the complications in the latest pregnancy and those with a previous history of the complications (stillbirth in the latest pregnancy, hazard ratio 1.08, 0.90 to 1.29; stillbirth in a previous pregnancy, hazard ratio 1.14, 1.05 to 1.23; preterm birth in the latest pregnancy, hazard ratio 1.10, 1.00 to 1.21; preterm birth in a previous pregnancy, hazard ratio 1.16, 1.08 to 1.26; small for gestational age in a previous pregnancy, hazard ratio 1.08, 1.00 to 1.16; small for gestational age in the latest pregnancy, 1.02, 0.96 to 1.09). Women with miscarriage or placental abruption in the latest pregnancy had a higher rate of hypertension than those without a history of the complications, but we found no association in those with a previous history of miscarriage or placental abruption (miscarriage in the latest pregnancy, hazard ratio 1.10, 1.06 to 1.14; miscarriage in previous pregnancy, hazard ratio 0.98, 0.95 to 1.01; placental abruption in the latest pregnancy, hazard ratio 1.26, 1.06 to 1.50; placental abruption in a previous pregnancy, hazard ratio 1.06, 0.93 to 1.21). Online supplemental figure S2 shows the unadjusted hazard ratios.

### Risk of type 2 diabetes mellitus

During a median follow-up of four years, 21 754 (1.5%) women developed type 2 diabetes mellitus. Figure 5 shows the cumulative incidence of type 2 diabetes mellitus in women with no history of the pregnancy complications, with pregnancy complications in the latest pregnancy, and with a previous history of the pregnancy complications. Incident type 2 diabetes mellitus rates were higher overall in women with pregnancy complications in the latest pregnancy or with a previous history of pregnancy complications than in those with no history of the pregnancy complications. Women with gestational diabetes mellitus, pre-eclampsia, and gestational hypertension in either a previous or the latest pregnancy had higher rates of type 2 diabetes mellitus than women with other pregnancy complications. Online supplemental table S6 shows the incidence rates of type 2 diabetes mellitus by history of pregnancy complications.

**Figure 5:**
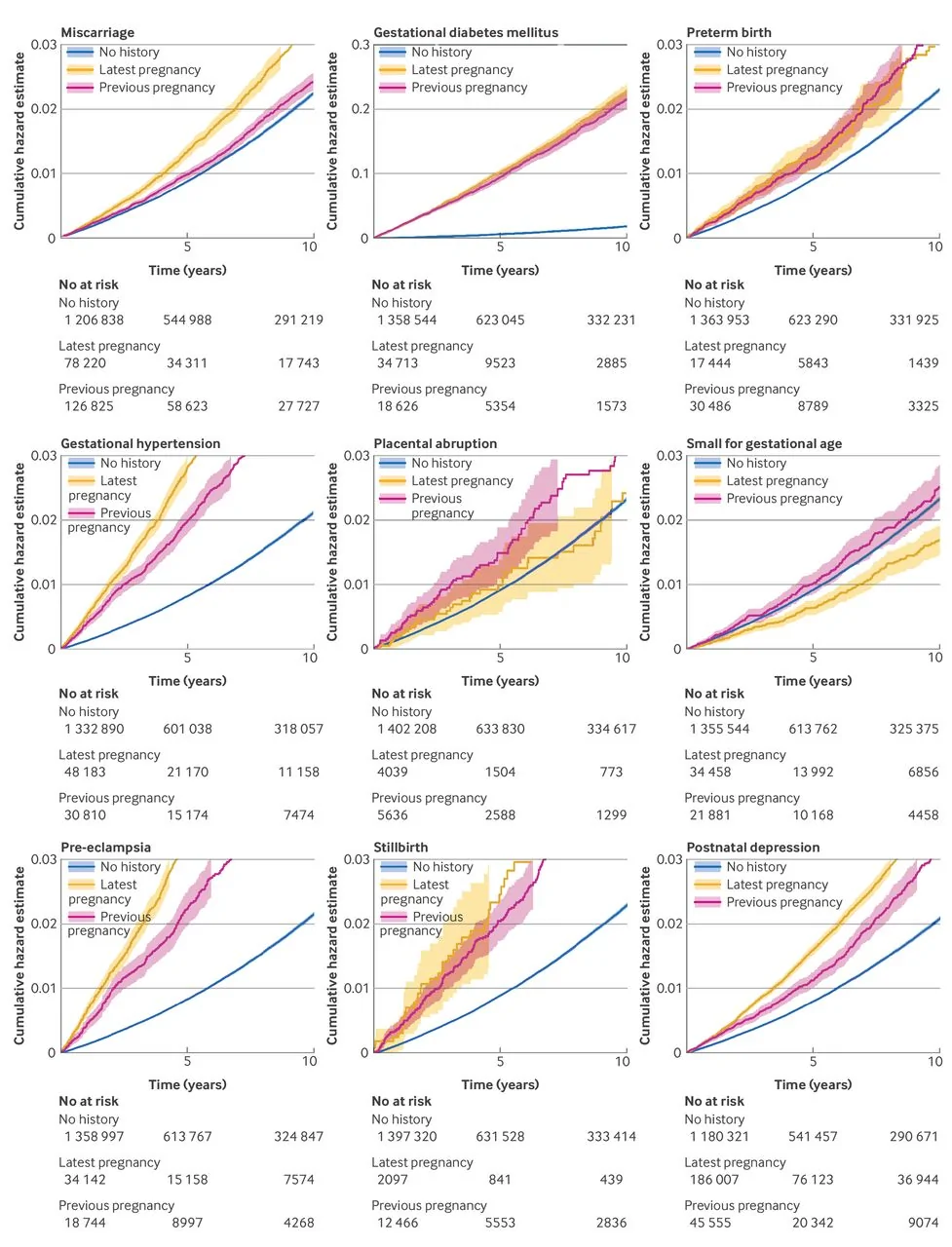
Cumulative hazard plots for type 2 diabetes in women with no history of the pregnancy complications, with pregnancy complications in the latest pregnancy, and with a previous history of the pregnancy complications

Figure 6 shows the hazard ratios of the association between pregnancy complications and type 2 diabetes mellitus, adjusting for traditional risk factors. Online supplemental table S7 provides a list of adjustment factors for each association.

**Figure 6:**
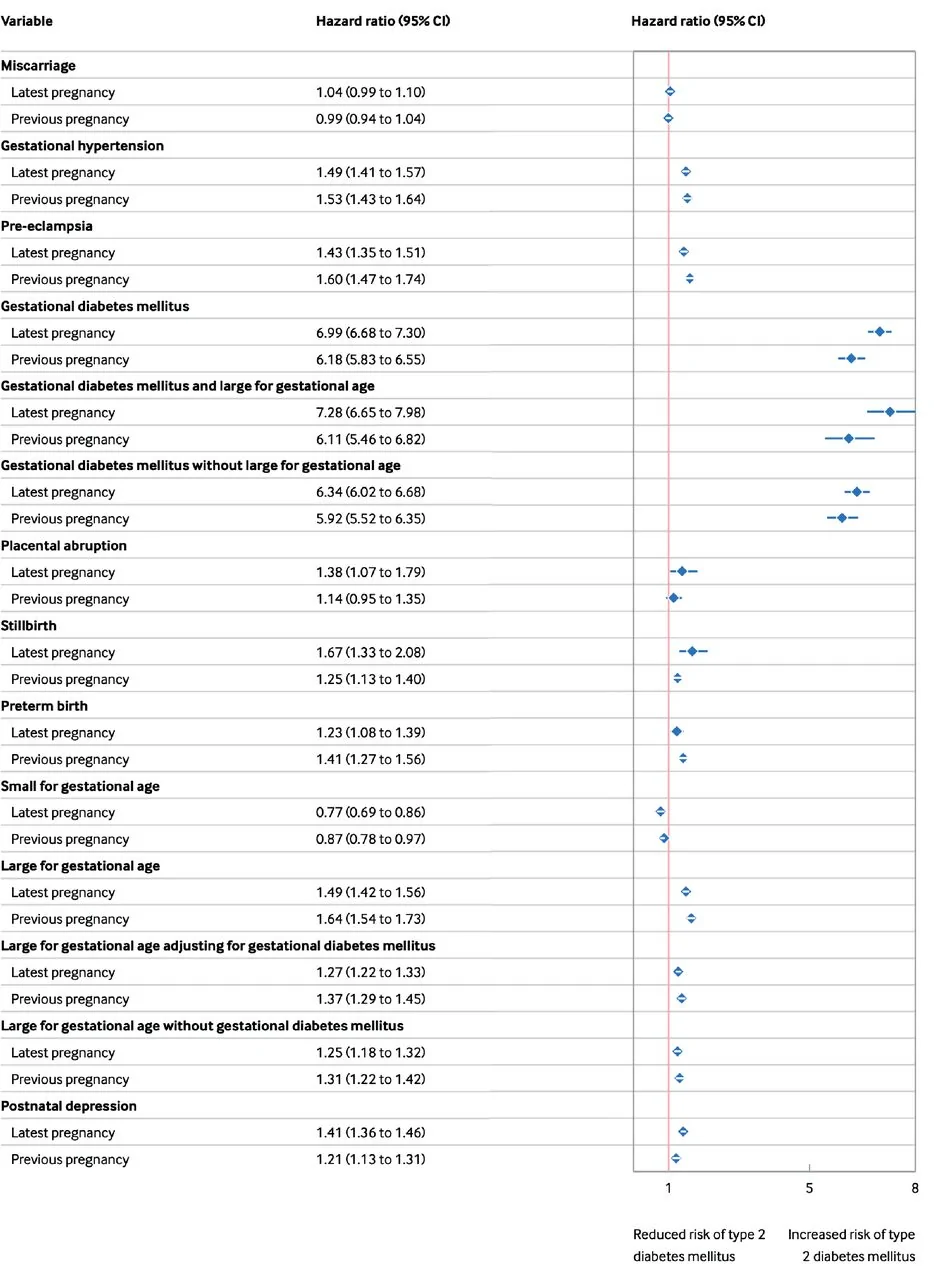
Adjusted hazard ratios of the association between pregnancy complications and type 2 diabetes mellitus in women with pregnancy complications in the latest pregnancy and with a previous history of the pregnancy complications, in comparison with women without a history of the complications

#### Well established associations

The rate of type 2 diabetes mellitus in women with gestational diabetes mellitus in the latest pregnancy was sevenfold (hazard ratio 6.99, 95% CI 6.68 to 7.30) and sixfold in women with a previous history of the pregnancy complication (hazard ratio 6.18, 5.83 to 6.55) compared with women without a history of the complication (figure 6). A higher rate of type 2 diabetes mellitus was also seen in women with both gestational diabetes mellitus and with a baby born large for gestational age (gestational diabetes mellitus with large for gestational age in the latest pregnancy, hazard ratio 7.28, 6.65 to 7.98; previous gestational diabetes mellitus with large for gestational age, hazard ratio 6.11, 5.46 to 6.82) and after excluding women with babies born large for gestational age in those with gestational diabetes mellitus (gestational diabetes mellitus without large for gestational age in the latest pregnancy, hazard ratio 6.34, 6.02 to 6.68; previous gestational diabetes mellitus without large for gestational age, hazard ratio 5.92, 5.52 to 6.35). Increased type 2 diabetes mellitus rates were seen following gestational hypertension and pre-eclampsia in both the most recent and in previous pregnancies (gestational hypertension in the latest pregnancy, hazard ratio 1.49, 1.41 to 1.57; previous gestational hypertension, hazard ratio 1.53, 1.43 to 1.64; pre-eclampsia in the latest pregnancy, hazard ratio 1.43, 1.35 to 1.51; previous pre-eclampsia, hazard ratio 1.60, 1.47 to 1.74).

#### Less well established associations

Women with a history of stillbirth, preterm birth, placental abruption, large-for-gestational-age birth, or postnatal depression also had higher rates of type 2 diabetes mellitus than women without these complications. Stillbirth was associated with an increased rate of type 2 diabetes mellitus in the most recent pregnancy (hazard ratio 1.67, 95% CI 1.33 to 2.08), with a weaker association for previous stillbirth (hazard ratio 1.25, 95% CI 1.13 to 1.40) (figure 6). Placental abruption was associated with a higher rate of type 2 diabetes mellitus in the most recent pregnancy (hazard ratio 1.38, 1.07 to 1.79), with no significant association for placental abruption in a previous pregnancy (hazard ratio 1.14, 0.95 to 1.35). Preterm birth was associated with a higher rate of type 2 diabetes mellitus in both the most recent (hazard ratio 1.23, 1.08 to 1.39) and previous (hazard ratio 1.41, 1.27 to 1.56) pregnancies. Large for gestational age was similarly associated with an increased rate of type 2 diabetes mellitus in the most recent pregnancy (hazard ratio 1.49, 1.42 to 1.56) and in previous pregnancies (hazard ratio 1.64, 1.54 to 1.73), as was postnatal depression (latest pregnancy, hazard ratio 1.41, 1.36 to 1.46; previous pregnancy, hazard ratio 1.21, 1.13 to 1.31). The association between large for gestational age and type 2 diabetes mellitus persisted after excluding women with gestational diabetes mellitus, both for the most recent pregnancy (hazard ratio 1.25, 1.18 to 1.32) and for previous pregnancies (hazard ratio 1.31, 1.22 to 1.42).

Women with babies born small for gestational age in the latest pregnancy or with a previous history of the complication had a lower rate of type 2 diabetes mellitus than women without a history of the complication (latest pregnancy, hazard ratio 0.77, 95% CI 0.69 to 0.86; previous pregnancy, hazard ratio 0.87, 0.78 to 0.97). We found no significant association between miscarriage and future risk of type 2 diabetes mellitus (latest pregnancy, hazard ratio 1.04, 0.99 to 1.10; previous pregnancy, hazard ratio 0.99, 0.94 to 1.04). Online supplemental figure S3 shows the unadjusted hazard ratios.

### Risk of cardiovascular disease

During follow-up, 16 069 (1.1%) women developed cardiovascular disease during a median follow-up of four years. Figure 7 shows the cumulative incidence of cardiovascular disease in women with no history of the pregnancy complications, with pregnancy complications in the latest pregnancy, and with a previous history of the pregnancy complications. Incident cardiovascular disease rates were higher overall in women with pregnancy complications in the latest or in a previous pregnancy than in those without a history of the pregnancy complications. Women with gestational diabetes mellitus, pre-eclampsia, gestational hypertension, and postnatal depression in the latest pregnancy had substantially higher rates of cardiovascular disease than women with other pregnancy complications. Online supplemental table S6 shows the incidence rates of cardiovascular disease by history of pregnancy complications.

**Figure 7:**
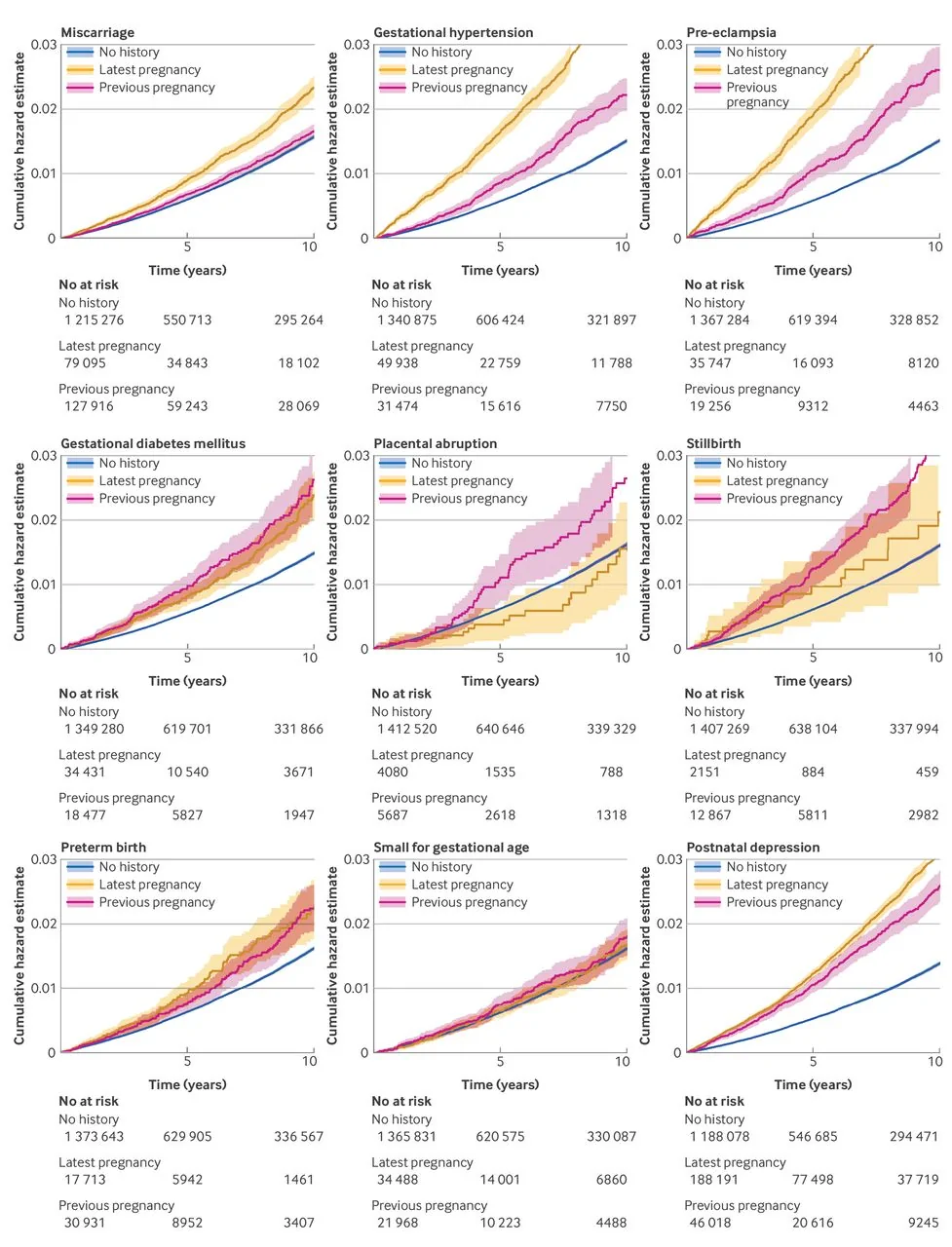
Cumulative hazard plots for cardiovascular disease in women with no history of the pregnancy complications, with pregnancy complications in the latest pregnancy, and with a previous history of the pregnancy complications

Figure 8 shows the hazard ratios of the association between pregnancy complications and cardiovascular disease, adjusting for traditional risk factors. Online supplemental table S7 provides a list of adjustment factors for each association.

**Figure 8:**
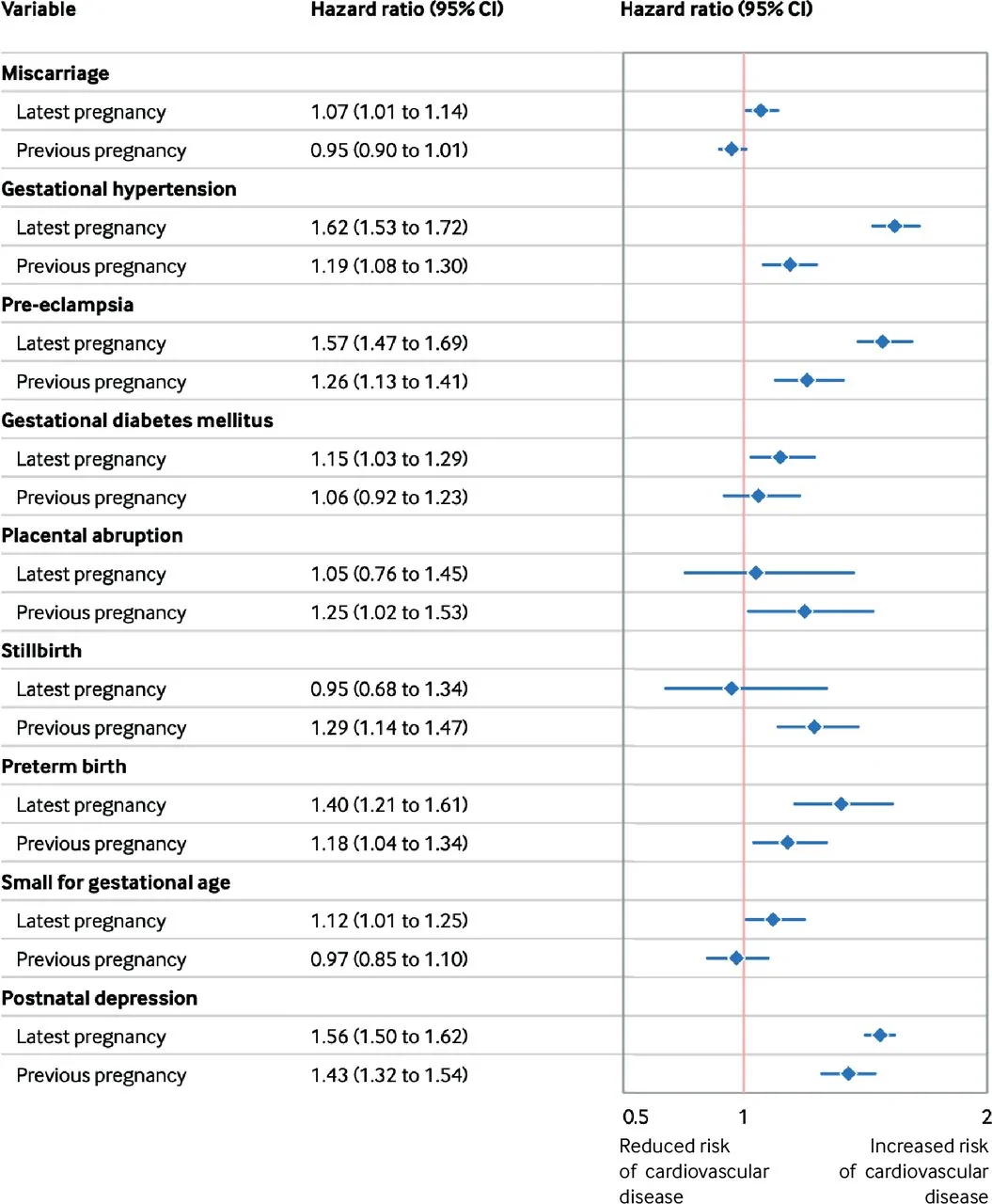
Adjusted hazard ratios of the association between pregnancy complications and cardiovascular disease in women with pregnancy complications in the latest pregnancy and with a previous history of the pregnancy complications, in comparison with women without a history of the complications

#### Well established associations

The rate of cardiovascular disease in women with gestational diabetes mellitus in the latest pregnancy was increased (hazard ratio 1.15, 95% CI 1.03 to 1.29) (figure 8). Although the rate was increased in those with previous gestational diabetes mellitus, the association was not significant (hazard ratio 1.06, 0.92 to 1.23). The rate of cardiovascular disease was also higher in women with either gestational hypertension or pre-eclampsia in the latest or in a previous pregnancy (gestational hypertension in the latest pregnancy, hazard ratio 1.62, 1.53 to 1.72; previous gestational hypertension, hazard ratio 1.19, 1.08 to 1.30; pre-eclampsia in the latest pregnancy, hazard ratio 1.57, 1.47 to 1.69; previous pre-eclampsia, hazard ratio 1.26, 1.13 to 1.41).

#### Less well established associations

The rate of cardiovascular disease was higher in women with either preterm birth or postnatal depression in the latest or in a previous pregnancy (preterm birth in the latest pregnancy, hazard ratio 1.40, 95% CI 1.21 to 1.61; previous preterm birth, hazard ratio 1.18, 1.04 to 1.34; postnatal depression in the latest pregnancy, hazard ratio 1.56, 1.50 to 1.62; previous postnatal depression, hazard ratio 1.43, 1.32 to 1.54) (figure 8). Women with stillbirth or placental abruption in the latest pregnancy or with a previous history of the complications had a higher rate of cardiovascular disease than women without a history of the complications, although the association in women with the pregnancy complications in the latest pregnancy was not significant (stillbirth in the latest pregnancy, hazard ratio 0.95, 0.68 to 1.34; previous stillbirth, hazard ratio 1.29, 1.14 to 1.47; placental abruption in the latest pregnancy, hazard ratio 1.05, 0.76 to 1.45; previous placental abruption, hazard ratio 1.25, 1.02 to 1.53). Women with babies born small for gestational age in the latest pregnancy had an increased risk of cardiovascular disease (hazard ratio 1.12, 1.01 to 1.25). We found no significant association between small for gestational age in a previous pregnancy and cardiovascular disease (hazard ratio 0.97, 0.85 to 1.10). Women with miscarriage in the latest pregnancy had a higher rate of cardiovascular disease than those without a history of the complication and we found no significant association between previous history of miscarriage and cardiovascular disease (miscarriage in the latest pregnancy, hazard ratio 1.07, 1.01 to 1.14; previous miscarriage, 0.95, 0.90 to 1.01). Online supplemental figure S4 shows the unadjusted hazard ratios.

### Risk of chronic kidney disease

During follow-up, 5288 (0.4%) women developed chronic kidney disease during a median follow-up of four years. Figure 9 shows the cumulative incidence of chronic kidney disease in women with no history of the pregnancy complications, with pregnancy complications in the latest pregnancy, and with a previous history of the pregnancy complications. Incident chronic kidney disease rates were higher overall in women with pregnancy complications in the latest or in a previous pregnancy than in those with no history of the pregnancy complications. Women with gestational diabetes mellitus, pre-eclampsia, or gestational hypertension in the latest pregnancy had higher rates of chronic kidney disease than women with other pregnancy complications. Online supplemental table S6 shows the incidence rates of chronic kidney disease by history of pregnancy complications.

**Figure 9:**
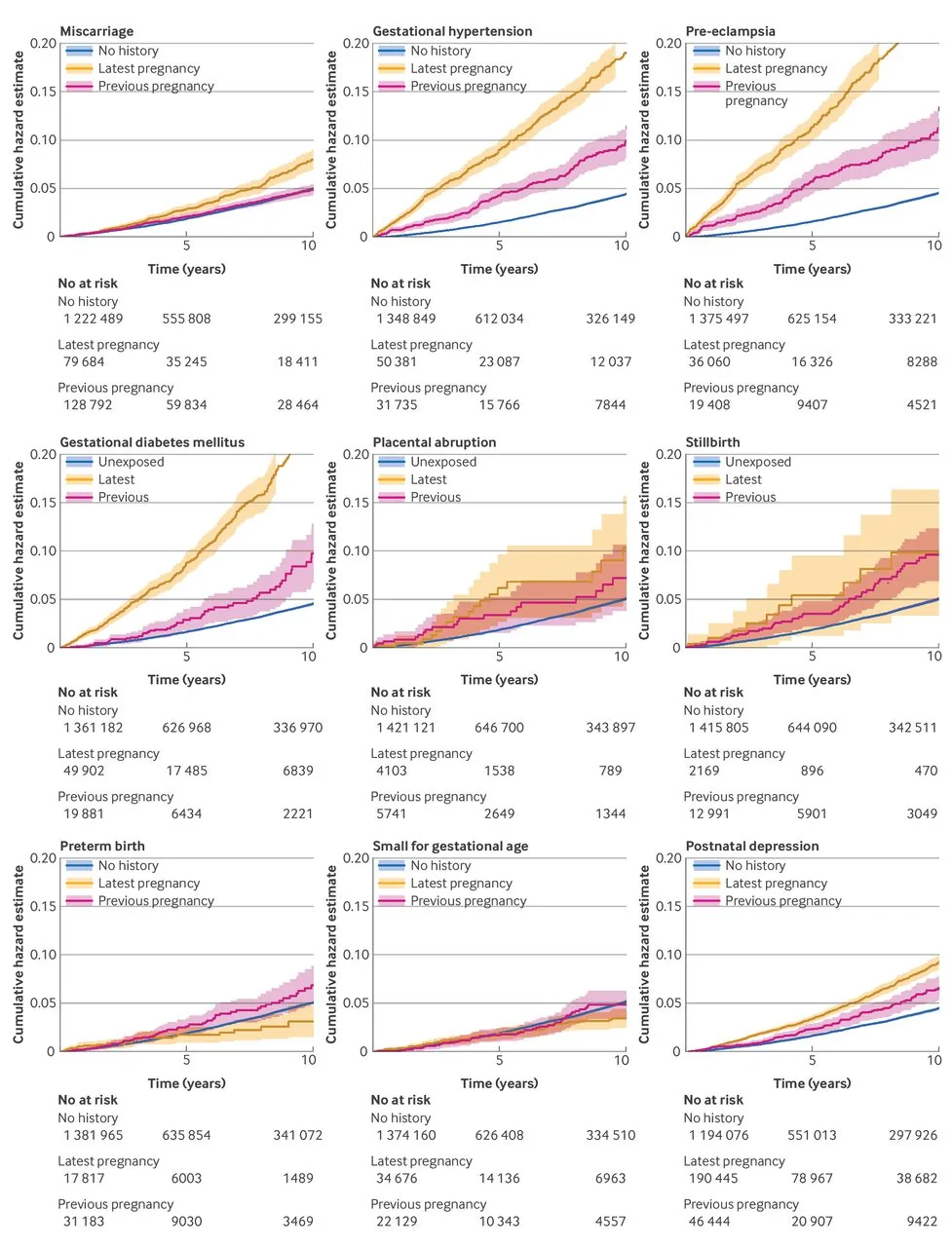
Cumulative hazard plots for chronic kidney disease in women with no history of the pregnancy complications, with pregnancy complications in the latest pregnancy, and with a previous history of the pregnancy complications

Figure 10 shows the hazard ratios of the association between pregnancy complications and chronic kidney disease, adjusting for traditional risk factors. Online supplemental table S7 provides a list of adjustment factors for each association.

**Figure 10:**
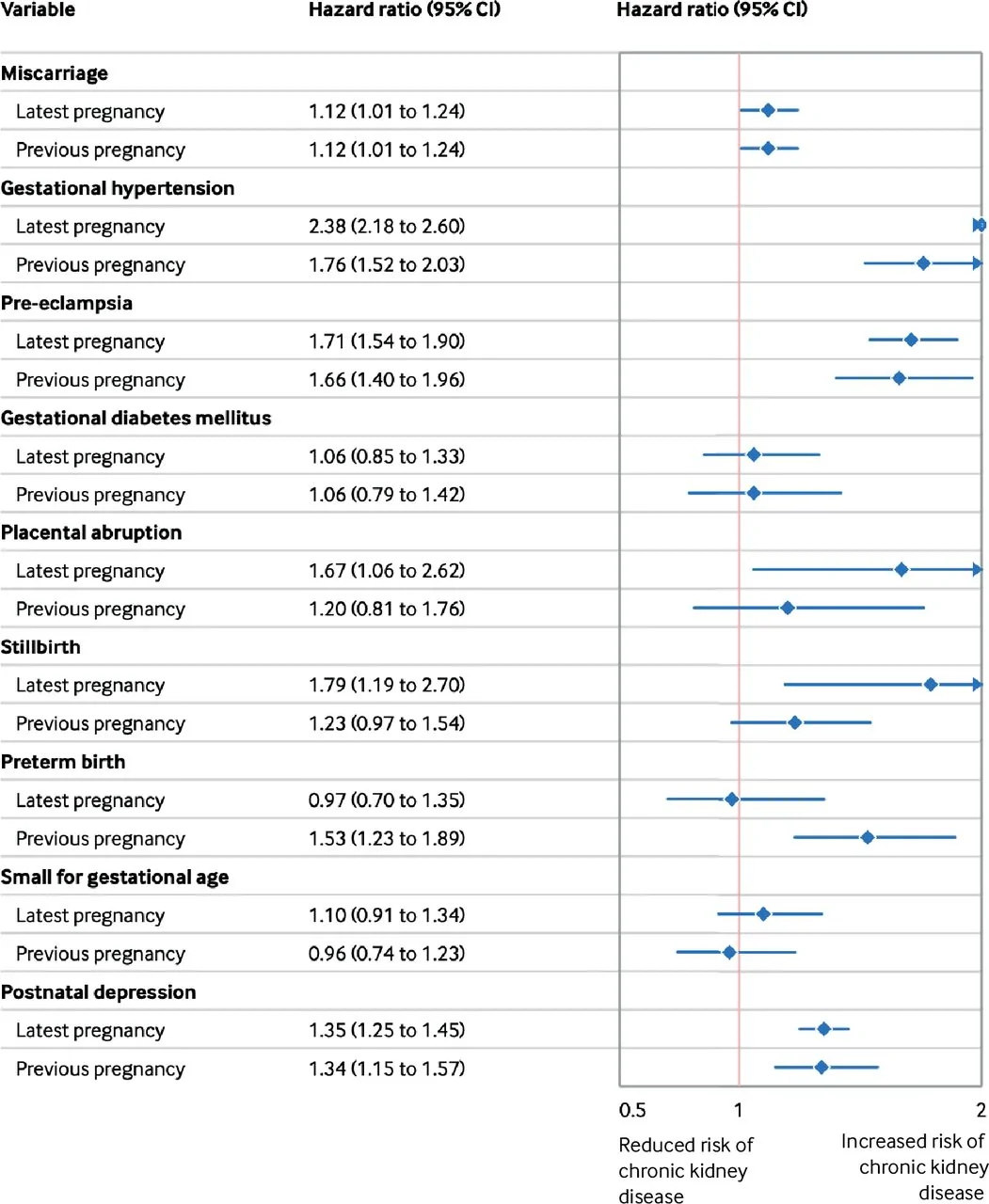
Adjusted hazard ratios of the association between pregnancy complications and chronic kidney disease in women with pregnancy complications in the latest pregnancy and with a previous history of the pregnancy complications, in comparison with women without a history of the complications

#### Well established associations

The rate of chronic kidney disease in women with gestational hypertension in the latest pregnancy was more than twofold higher (hazard ratio 2.38, 95% CI 2.18 to 2.60) and was still increased in women with a previous history of gestational hypertension (hazard ratio 1.76, 1.52 to 2.03) compared with women without a history of the complication (figure 10). The rate of chronic kidney disease was also higher in women with pre-eclampsia in the latest or in a previous pregnancy (pre-eclampsia in the latest pregnancy, hazard ratio 1.71, 1.54 to 1.90; previous pre-eclampsia, hazard ratio 1.66, 1.40 to 1.96). Although we found a positive association between gestational diabetes mellitus and chronic kidney disease, the association was not significant (gestational diabetes mellitus in the latest pregnancy, hazard ratio 1.06, 0.85 to 1.33; previous gestational diabetes mellitus, hazard ratio 1.06, 0.79 to 1.42).

#### Less well established associations

The rate of chronic kidney disease was higher in women with either postnatal depression or miscarriage in the latest or in a previous pregnancy (postnatal depression in the latest pregnancy, hazard ratio 1.35, 95% CI 1.25 to 1.45; previous postnatal depression, hazard ratio 1.34, 1.15 to 1.57; miscarriage in the latest pregnancy, hazard ratio 1.12, 1.01 to 1.24; previous miscarriage, hazard ratio 1.12, 1.01 to 1.24) than in those without a history of these complications, and the rate was similar in those with the complications in the latest or in a previous pregnancy (figure 10). Women with placental abruption or stillbirth in the latest pregnancy had a higher rate of chronic kidney disease than women without a history of these complications, although the association in women with previous placental abruption or stillbirth was not significant (placental abruption in the latest pregnancy, hazard ratio 1.67, 1.06 to 2.62; previous placental abruption, hazard ratio 1.20, 0.81 to 1.76; stillbirth in the latest pregnancy, hazard ratio 1.79, 1.19 to 2.70; previous stillbirth, hazard ratio 1.23, 0.97 to 1.54). In contrast, preterm birth was associated with a higher rate of chronic kidney disease in women with a previous history of the complication (hazard ratio 1.53, 1.23 to 1.89) but not in those with preterm birth in the latest pregnancy (hazard ratio 0.97, 0.70 to 1.35). Although we found a positive association between small for gestational age and chronic kidney disease, the association was not significant (small for gestational age in the latest pregnancy, hazard ratio 1.10, 0.91 to 1.34; previous small for gestational age, hazard ratio 0.96, 0.74 to 1.23). Online supplemental figure S5 shows the unadjusted hazard ratios.

### Adjustment for multiple testing

Of the 72 associations examined, 53 were significant before correction and 51 remained significant after Benjamini-Hochberg false discovery rate correction. Online supplemental table S8 shows the P values before and after adjusting for multiple testing in each association. We found no marked differences in >99% of the associations, with some differences for two associations (ie, between previous miscarriage and chronic kidney disease and between previous placental abruption and cardiovascular disease).

### Sensitivity analyses

The proportional hazards assumption was evaluated for all covariates and variables and was found to be fulfilled for all variables except for corticosteroid drug treatment and missing smoking status in the analysis of cardiovascular disease and chronic kidney disease outcomes. After introducing an interaction term for these variables with time in the regression, we found no significant difference in the estimates compared with models that assumed proportional hazards (online supplemental figure S6 and S7). In sensitivity analyses with the first recorded pregnancy and a randomly selected pregnancy as the index, online supplemental table S9 provides the corresponding cohort sizes, outcome counts, and median follow-up times. The main findings were reproduced under both definitions: no association reversed direction and the strong associations for hypertensive disorders of pregnancy, gestational diabetes mellitus, and postnatal depression remained substantial and significant. Differences were confined to a small number of complications with fewer events and effect estimates close to the null where borderline associations moved between significance and non-significance without changing direction (online supplemental figures S8a–d).

## Discussion

### Principal findings

The aim of the study was to assess the association between several pregnancy complications and the risk of developing cardiometabolic-renal disease in subsequent years after the pregnancy, accounting for a number of confounders, in women who were pregnant in the UK. Our study showed that women who had pregnancy complications in the latest (most recent) pregnancy or had a previous history of complications (before the most recent pregnancy) were at increased risk of developing cardiometabolic-renal conditions in subsequent years after the pregnancy. Gestational hypertension and pre-eclampsia in the most recent or a previous pregnancy were associated with increased rates for all of the four cardiometabolic-renal outcomes studied. Gestational diabetes mellitus was strongly associated with subsequent type 2 diabetes mellitus and was also associated with increased rates of cardiovascular disease and hypertension but not chronic kidney disease. Similarly, postnatal depression in either the latest or a previous pregnancy was associated with an increased rate of developing cardiometabolic-renal conditions, especially cardiovascular disease.

Women with a preterm birth in the latest or in a previous pregnancy also had an increased risk of each of the cardiometabolic-renal conditions but only women with a previous history of preterm birth had an increased risk of developing future chronic kidney disease. Stillbirth was associated with an increased risk of type 2 diabetes mellitus regardless of whether it occurred in the most recent or a previous pregnancy, whereas stillbirth was associated with higher rates of hypertension and cardiovascular disease, and stillbirth in the most recent pregnancy was associated with higher rates of chronic kidney disease. Compared with women without a history of placental abruption, women with placental abruption in the latest pregnancy had an increased rate of subsequent hypertension, chronic kidney disease, and type 2 diabetes mellitus, whereas those with a previous history had an increased rate of cardiovascular disease.

Women with a previous history of babies born small for gestational age had an increased rate of developing hypertension and cardiovascular disease and those with babies born small for gestational age in the latest pregnancy had a reduced rate of developing type 2 diabetes mellitus. Women with babies born large for gestational age in the latest or in a previous pregnancy (with and without gestational diabetes mellitus) had increased rates of type 2 diabetes mellitus. Women with a miscarriage in the latest pregnancy had a higher incidence of future hypertension and cardiovascular disease than those with a previous history of the complication, who had higher rates of developing chronic kidney disease. We found no significant association between miscarriage and type 2 diabetes mellitus.

These findings should be interpreted within a prognostic rather than a causal framework. Several pregnancy complications share pathophysiological pathways, such as metabolic dysfunction underlying both gestational diabetes mellitus and macrosomia, or vascular dysfunction underlying pre-eclampsia, placental abruption, and stillbirth. As such, the increased risk of cardiometabolic-renal disease seen after a complication does not imply that the complication itself is the causal mechanism. Instead, the complication functions as a clinical marker of underlying maternal susceptibility. Our hazard ratios should therefore not be viewed as additive or as representing the combined causal effect of multiple complications.

### Comparison with other studies and potential biological mechanism of association

#### Gestational diabetes mellitus, gestational hypertension, and pre-eclampsia

Our findings are consistent with previous studies on the association between adverse pregnancy outcomes and risk of future cardiometabolic-renal conditions. Previous reviews and a prospective cohort study from the US found that women with gestational diabetes mellitus had about a sevenfold higher risk of type 2 diabetes mellitus and a 1.5-2-fold increased risk of cardiovascular disease, hypertension, and chronic kidney disease.^9 34 35^ A cohort study based on a Swedish registry reported a 2-6-fold higher risk of chronic hypertension and a 1.5-2-fold increased risk of cardiovascular disease or chronic kidney disease in patients with a history of pre-eclampsia or gestational hypertension, similar to our findings.^10 36–38^ How gestational hypertension and pre-eclampsia increase the risk of future hypertension, type 2 diabetes, cardiovascular disease, and chronic kidney disease is not fully understood,^39 40^ but may be mediated through insulin resistance.^41 42^ Shared genetic factors between gestational diabetes mellitus and type 2 diabetes mellitus have been hypothesised to be the link between the two conditions.^37 38^ A history of gestational hypertension or pre-eclampsia could be indicative of underlying insulin resistance, worsened by heightened metabolic demands during pregnancy, as occurs in gestational diabetes mellitus. As well as insulin resistance, mutual risk factors exist for these adverse pregnancy outcomes and cardiometabolic disease, such as age, hypertension, and body mass index, and may explain the relation between pregnancy complications and long term conditions.^43 44^ Pregnancy could also act as a trigger and accelerate cardiometabolic-renal conditions.

#### Postpartum depression

Few studies have explored the association between postnatal depression and the risk of developing chronic conditions in subsequent years after pregnancy. Some studies have reported an increased risk of future cardiovascular disease (36%), chronic hypertension (32%), and chronic kidney disease (38%).^45–48^ Also, depression in general was associated with a 1.25-fold higher risk of chronic kidney disease.^49^ Most studies evaluated the association between type 2 diabetes and the risk of depression, but not the reverse association. A study on the bidirectional association between depression and type 2 diabetes, however, showed a 1.25-fold increase in the risk of type 2 diabetes mellitus in women with a history of depression.^50^ Postpartum depression often involves chronic inflammation and dysregulation of the hypothalamic-pituitary-adrenal axis, resulting in raised cortisol levels and insulin resistance.^51–53^ These factors contribute to metabolic syndrome, increasing the risk of type 2 diabetes mellitus. Chronic stress and depression can also exacerbate endothelial dysfunction and contribute to hypertension and cardiovascular disease by promoting inflammatory responses and altering vascular function.^47 54^ Also, the stress related hormonal imbalances and lifestyle changes associated with postpartum depression, such as poor diet and physical inactivity, can further increase the risk of cardiometabolic renal conditions.^55^

#### Pregnancy loss

Previous research indicated that women who had a miscarriage or stillbirth may have a 1-2-fold increased risk of cardiometabolic-renal conditions.^3 19 56–60^ Although we found an association between miscarriage and cardiovascular disease, hypertension, and chronic kidney disease, we found no significant association with type 2 diabetes. This lack of association between miscarriage and type 2 diabetes is supported by a recent meta-analysis.^61^ The increased risk of cardiometabolic conditions in subsequent years after a stillbirth is likely to be linked to other associated pregnancy complications linked to stillbirth, such as pre-eclampsia, intrauterine growth restriction, hypertensive disorders of pregnancy, and pre-existing diabetes, rather than stillbirth itself.^62^

#### Preterm birth

Previous studies showed that preterm birth was associated with a 1.4-fold higher risk of cardiovascular disease.^63^ Also, preterm birth was linked to a 1.6-fold increased risk of developing chronic hypertension, 1.5-fold increased risk of type 2 diabetes mellitus, and 1.7-fold higher risk of chronic kidney disease.^63–65^ Preterm birth is often linked to underlying maternal conditions, such as chronic inflammation, metabolic syndrome, and endothelial dysfunction, which are known risk factors for these chronic diseases.^66^ The complications leading to preterm birth may also trigger systemic inflammation which might contribute to long term hypertension and cardiovascular disease.^62^ The association between preterm birth and cardiometabolic conditions could also be explained by placental dysfunction, which is associated with long term cardiometabolic risks.

Preterm birth is a heterogeneous outcome comprising a wide range of gestational ages and underlying clinical pathways. Although we identified preterm birth in this study based on routinely recorded primary care medical codes and linked hospital ICD-10 (international classification of diseases, 10th revision) codes, these data sources do not consistently distinguish spontaneous from medically indicated preterm birth or allow grouping by more granular gestational age categories across all pregnancies. As a result, the observed associations should be interpreted as average effects across the spectrum of preterm deliveries rather than applying uniformly to all forms of preterm birth. Given the high incidence of preterm birth, future studies with more granular obstetric information are needed to examine whether cardiometabolic-renal risk differs by subtype or degree of prematurity.

#### Placental abruption

Previous research indicated that women who had placental abruption had a higher risk of developing chronic hypertension and cardiovascular disease.^67 68^ This association may be a result of underlying vascular dysfunction that increases the risk of both abruption and future cardiovascular risks. We have also found that women with a history of placental abruption may have an increased risk of type 2 diabetes mellitus and chronic kidney disease, which has not been reported before. Placental abruption has been linked to chronic inflammation, endothelial dysfunction, and hypercoagulability, all of which can persist after pregnancy, increasing the risk of future cardiometabolic-renal conditions.^69^ Also, placental abruption is strongly associated with pre-eclampsia, which increases the risk of future cardiometabolic-renal conditions.

#### Small and large for gestational age

Previous literature reported an association between small for gestational age and cardiovascular disease or hypertension.^70 71^ Also, similar to other studies, women who delivered babies born small for gestational age had an increased risk of developing hypertension, even after adjusting for pre-eclampsia, but the risk of type 2 diabetes only increased in women who delivered a baby born large for gestational age (with or without gestational diabetes mellitus). This finding could be because delivering a baby large for gestational age may imply pre-existing metabolic dysfunction, whereas delivering a baby small for gestational age may indicate pre-existing vascular dysfunction.^71^ These findings might also explain the lack of association between small for gestational age and chronic kidney disease in our study. The strong association between gestational diabetes mellitus and large for gestational age would also explain the subsequent higher rate of type 2 diabetes mellitus in women who delivered a baby large for gestational age.

In summary, as well as confirming well established associations, our study provides several new insights. We identified consistent associations between postnatal depression and an increased risk of all four cardiometabolic-renal outcomes, supporting a potential role for psychological and neuroendocrine pathways in long term vascular and metabolic health. We also found new associations between placental abruption and subsequent type 2 diabetes mellitus or chronic kidney disease. Furthermore, by evaluating complications in both the latest and previous pregnancies, we showed that the cardiometabolic risk persisted, even when the complication occurred years before the most recent pregnancy. This finding indicates that an increased cardiometabolic-renal risk is already evident in the subsequent years after pregnancy. Pregnancy complications were not conceptualised as hierarchical or causal intermediates, but as separate clinical signals that may co-occur. The adjusted models aimed to avoid confounding rather than deconstruct causal pathways. Hazard ratios should not be summed across complications, but instead each represents the incremental risk associated with that specific history relative to women without that history.

### Clinical implications and opportunities for prevention

As well as highlighting the need for closer postpartum monitoring, our findings have broader implications for clinical practice. Firstly, our findings support incorporating a comprehensive reproductive history, including complications occurring in earlier pregnancies, into routine cardiometabolic risk assessment. Secondly, several risk markers identified in this study, such as postnatal depression and placental abruption, are not currently included in the commonly used risk prediction tools (eg, QRISK-3 and QDiabetes algorithms), and our results suggest that their inclusion may improve early identification of women at increased risk. Thirdly, the occurrence of pregnancy complications represents an opportunity for targeted preventive interventions, including structured postpartum screening for hypertension, diabetes, and chronic kidney disease, lifestyle and weight management programmes, and enhanced mental health support. Fourthly, our findings highlight the need for integrated postpartum care pathways involving primary care, cardiology, nephrology, endocrinology, and mental health services. Collectively, these implications extend the importance of pregnancy complications beyond immediate obstetric outcomes, establishing these complications as early indicators of cardiometabolic-renal risk in the subsequent years after pregnancy.

Although the study period spanned more than two decades, median follow-up was about 4.3 years, reflecting the open cohort design of the CPRD Aurum and the staggered entry of participants over calendar time. Follow-up started six months post partum, and women were censored at the earliest of when the outcome occurred, transfer to another general practice, death, or the end of data collection. Consequently, many women contributed follow-up time that was more recent, resulting in a shorter median duration despite the overall calendar period being long. A median follow-up time of about four years is characteristic of large UK primary care electronic health record cohorts studying cardiometabolic outcomes*.*^23 72^ As a result, our findings mainly reflect cardiometabolic-renal risk in the postpartum period and subsequent years rather than lifetime risk. This timeframe is clinically important because several cardiometabolic conditions emerge within a few years after pregnancy, and this period represents an opportune time for early identification, risk stratification, and preventive intervention. Longer term follow-up will be important to understand whether these risks change later in life.

### Strengths and limitations of this study

Our study had many strengths. Firstly, we used the CPRD Aurum database, which contains electronic health records from >12 million patients, reflecting a nationally representative sample of the UK population in terms of geography, deprivation, and age.^73^ A key strength of this study was the use of a large population based primary care database rather than reliance on hospital registers. CPRD Aurum captures pregnancy complications and cardiometabolic-renal outcomes across the full severity spectrum, including conditions managed entirely in primary care that may not result in hospital admission. In contrast, hospital based datasets tend to over-represent more severe or acute presentations, which may inflate effect estimates and limit generalisability. By using routinely collected primary care data with linkage to secondary care records, our study provided a comprehensive and representative assessment of the risk of cardiometabolic-renal disease after pregnancy complications in the general population. Secondly, in contrast with previous studies, we adjusted for a broader range of relevant established risk factors, enabling us to assess whether pregnancy complications provide prognostic information beyond established risk factors. Thirdly, the cardiometabolic-renal outcomes considered in this study are included in an incentive scheme for the management of chronic conditions, which means that the outcomes are likely to be more accurately coded.^74^

The study had some limitations. Firstly, for the drug treatments used as confounders, we acknowledge that a prescription recorded in primary care records does not guarantee that the prescribed drug was consumed by the patient or reflect compliance with drug treatments, which could also change over time. Secondly, although we adjusted for several known and potential confounders, the possibility of unmeasured confounding remains (eg, we could not adjust for dietary habits and physical activity because this information is not well recorded in UK primary care data). Also, data for ethnic group were missing for >20% of the cohort, reflecting known limitations of routinely collected primary care records in the UK. We included ethnic group with a missing category approach to maximise cohort retention and reduce selection bias, but residual confounding related to incomplete ethnic group recording cannot be fully excluded, particularly if missingness was not random. Also, pregnancy related acute kidney injury, an established risk factor for subsequent chronic kidney disease, could not be reliably captured because of incomplete and inconsistent recording in routine primary care data and was therefore not included in the adjustment set.

Thirdly, although we used CPRD Aurum pregnancy register data together with primary care SNOMED CT codes and linked Hospital Episodes Statistics ICD-10 codes to identify pregnancy complications such as miscarriage, some patients were likely to have been missed. Recent methodological evaluations of both CPRD Aurum and CPRD GOLD showed that a proportion of pregnancies have an unknown outcome, which may reflect early pregnancy losses that were not coded in primary care or did not result in hospital attendance. Therefore, although our multi-source approach improved completeness, under-reporting of miscarriage, particularly early miscarriage or clinically unconfirmed losses, is possible.^75^

A further limitation was determining outcomes. Because hypertension and type 2 diabetes mellitus are under-diagnosed, women with a pregnancy complication may attend more often and be more likely to have blood pressure or blood glucose recorded, as evidenced in our data (online supplemental tables S10–13), and therefore differential detection could contribute to the observed associations. Although these outcomes are systematically recorded under the Quality and Outcomes Framework, higher detection in more closely monitored women cannot be excluded and should be considered when interpreting our findings. This observation seemed to be largely confined to gestational diabetes mellitus, gestational hypertension, and pre-eclampsia, consistent with guideline mandated follow-up rather than generic surveillance. Postnatal depression showed a similar, less guideline specific increase in measurement and consultations that persisted after excluding women with these complications, whereas differences for other complications were modest. The direction of interpretation of these differences is not straightforward. A greater frequency of measurement and consultation among women with a current or previous pregnancy complication may reflect closer monitoring by the clinician. This behaviour, however, is equally consistent with the early clinical course of the outcome itself, because women in whom hypertension or type 2 diabetes is developing are likely to consult more often and to have borderline values recorded in the months preceding a formal diagnosis. Contact with healthcare professionals during follow-up is thus partly a consequence of the disease process under study and both mechanisms would generate a similar pattern in these data. We therefore interpret the observed differences as outlining the scope for, rather than showing, ascertainment bias.

Fourthly, because pregnancy complications may cluster within the same individual, and some complications may lie on the same biological pathway, our estimates should not be interpreted as independent causal effects. Although we adjusted for key related complications, such as pre-eclampsia in regression models involving placental abruption, preterm birth, or small for gestational age, residual confounding by shared underlying determinants is possible. Nonetheless, from a prognostic perspective, the presence of each complication continues to provide clinically meaningful information on the future risk of cardiometabolic-renal disease. Furthermore, some of the pregnancy complications are closely linked (eg, pre-eclampsia is a cause of placental abruption, preterm birth, and small for gestational age) and although we adjusted for pre-eclampsia in regression models for these pregnancy complications, the results should be interpreted with caution.

Lastly, although we distinguished between complications occurring in the most recent pregnancy and those occurring in any previous pregnancy, we did not explicitly model recurrence of the same pregnancy complication across multiple pregnancies. History of pregnancy complications category reflected whether a woman had ever had a specific complication in her most recent pregnancy or in any previous pregnancy, rather than the number of times the complication occurred. As such, our estimates represent the prognostic association of having a history of a complication rather than the incremental risk associated with recurrent events. A further limitation arose from fixing the index date at the latest pregnancy and excluding women with the outcome before this date. Women with a higher underlying risk are on average more likely to have developed the outcome before their latest pregnancy and so be excluded, and therefore in the previous complication groups, women at lower risk could be over-represented. The associations for previous complications were therefore likely to be conservative rather than overstated. In the sensitivity analyses with the first and a randomly selected pregnancy as the index, most associations were unchanged, with differences confined to a few complications with sparse events and estimates close to the null. Future research could use a time varying inception cohort design, modelling the number of pregnancies and the accrual of complications as time dependent variables from first delivery, to make more complete use of available follow-up.

### Conclusions

Women with a pregnancy complication in the latest pregnancy or in a previous pregnancy had an increased risk of cardiometabolic-renal disease in the subsequent years after pregnancy. More research is needed to better understand the underlying mechanisms behind these associations and to assess whether incorporating the history of pregnancy complications into existing risk prediction models for cardiovascular disease, type 2 diabetes, hypertension, and chronic kidney disease would improve the prediction of these conditions in women who have been pregnant. Guidelines for preventing or reducing the risk of cardiometabolic-renal conditions could recommend that healthcare professionals record a detailed pregnancy history from women to better assess the risk of future cardiometabolic-renal conditions and reduce future morbidity and mortality from cardiometabolic-renal disease and improve maternal long term health.

10.1136/bmjmed-2026-003035.supp2 Supplementary data

10.1136/bmjmed-2026-003035.supp3 Supplementary data

## Data Availability

Data may be obtained from a third party and are not publicly available. The datasets used for this study are available from the Clinical Practice Research Datalink (CPRD) and can be accessed upon reasonable request.
