# Supplementary material for: Pregnancy complications and risk of cardiometabolic-renal conditions in 1.4 million women in England: longitudinal study
Source: BMJ Med. 2026 Oct 6;5(1):e003035. doi: 10.1136/bmjmed-2026-003035 (PMC13647177; doi:10.1136/bmjmed-2026-003035)
Supplement: Supplementary file 1 [file bmjmed-5-1-s001.pdf]

Table of Contents

Table 1: Definitions of covariates and outcomes **3**

Baseline characteristics by exposure status ***6***

Hypertension outcome cohort **6**

Table 2: Baseline Characteristics by exposure status in the Hypertension cohort 6
Table 2 (continued): Baseline Characteristics by exposure status in the Hypertension cohort....... 8
Table 2 (continued): Baseline Characteristics by exposure status in the Hypertension cohort....... 10

Type 2 diabetes outcome cohort **13**

Table 3: Baseline Characteristics by exposure status in the T2DM cohort 13
Table 3 (continued): Baseline Characteristics by exposure status in the T2DM cohort 15
Table 3 (continued): Baseline Characteristics by exposure status in the T2DM cohort 17

Cardiovascular disease outcome cohort **20**

Table 4: Baseline Characteristics by exposure status in the CVD cohort 20
Table 4 (continued): Baseline Characteristics by exposure status in the CVD cohort....... 23
Table 4 (continued): Baseline Characteristics by exposure status in the CVD cohort....... 25

Chronic Kidney Disease (CKD) outcome cohort **27**

Table 5: Baseline Characteristics by exposure status in the CKD cohort 27
Table 5 (continued): Baseline Characteristics by exposure status in the CKD cohort....... 30
Table 5 (continued): Baseline Characteristics by exposure status in the CKD cohort....... 32

Incidence rates ***36***

Table 6: Incidence rates for hypertension, type 2 diabetes, cardiovascular disease and chronic kidney disease in women with latest, previous and without a history of pregnancy complications **36**

Table 6 (continued): Incidence rates for hypertension, type 2 diabetes, cardiovascular disease and chronic kidney disease in women with latest, previous and without a history of pregnancy complications **37**

Table 7: Adjustment factors in each regression model ***38***

Figure 1a: DAG for the association between pregnancy complications and cardiovascular disease. ***44***

Figure 1b: DAG for association between pregnancy complications and type 2 diabetes (T2DM) ***45***

Figure 1c: DAG for association between pregnancy complications and hypertension ***46***

Figure 1d: DAG for association between pregnancy complications and chronic kidney disease ***47***

Unadjusted Hazard Ratios ***48***

Figure 2: Unadjusted hazard ratios for hypertension outcome **48**

Figure 3: Unadjusted hazard ratios for type 2 diabetes outcome **49**

Figure 4: Unadjusted hazard ratios for CVD outcome....... **50**

Figure 5: Unadjusted hazard ratios for CKD outcome **51**

Sensitivity analysis after adding time interaction to variables violating the Cox PH Assumption ... ***52***

Figure 6: Adjusted hazard ratios for CVD ... **52**

Figure 7: Adjusted hazard ratios for CKD ... **53**

Multiple testing adjustment ... ***54***

Table 8: P-values adjusted for multiple testing ... **54**

Sensitivity analyses ... ***57***

Table 9: Comparison of cohort size, outcome counts and median follow-up between the primary (latest-pregnancy) analysis and the random- and first-pregnancy sensitivity analyses. ... **57**

Pregnancy complications and long-term maternal cardiometabolic outcomes: adjusted estimates from primary (last-pregnancy) and sensitivity (random- and first-pregnancy) analyses ... **58**

Figure 8a: Risk of hypertension ... 58
Figure 8b: Risk of T2DM ... 59
Figure 8c: Risk of CVD ... 60
Figure 8d: Risk of CKD ... 61

Proportion of women with a recorded SBP, HbA1c and eGFR measurement, and number of consultations, within 1 year of the index date, by exposure status ... **62**

Table 10: Hypertension cohort ... 62
Table 11: T2DM cohort ... 63
Table 12: CVD cohort ... 64
Table 13: CKD cohort ... 65

Table 1: Definitions of covariates and outcomes

Variable		
Continuous variable	Age	Age at baseline
	Body Mass Index	Latest record before index date
	Systolic Blood Pressure	Latest record before index date
Categorical variables	Ethnicity	Last recorded code before index date. To obtain the nine categories of ethnicity in QRISK3, we used medical codes from a previous study that externally validated QRISK3 (52). We used these together with ethnicity categories provided by CPRD and linked to Hospital Episodes Statistics. The CPRD categories include Asian, Black, Mixed, Others, White and Unknown according to office for national statistics (ONS) ethnicity categories (53). The definitions of these categories is found on ONS website here (54).
	Smoking status	Latest recorded before index date. Smoking status was defined using medical codes as (non-smoker, ex-smoker, light smoker (1-9 cigarettes/day), moderate smoker (10-19 cigarettes/day), or heavy smoker (≥20 cigarettes/day)), similar to QRISK3.
	Index of Multiple Depression (IMD)	Latest recorded before index date. Categorised into 5 levels. IMD 1 (most deprived) to IMD 5 (least deprived)
Alcohol misuse (Yes/No)		Latest record before index date
Medications (Yes/No)	Antihypertensive medication (Treated hypertension)	Whether woman has had at least two prescriptions of antihypertensive agent, with the most recent one no more than 28 days before index date similar to QRISK3.
	Corticosteroids	Whether woman has had at least two prescriptions of Corticosteroids, with the most recent one no more than 28 days before index date similar to QRISK3.
	Atypical antipsychotics	Whether woman has had at least two prescriptions of atypical antipsychotic medication, with the most recent one no more than 28 days before index date similar to QRISK3.
	Statins	Whether woman has had at least two prescriptions of statins medication, with the most recent one no more than 28

		days before index date similar to QDiabetes.
Family history (Yes/No)	Family history of coronary artery disease in first degree relative <60 years	Present if ever recorded before index date
	Family history of diabetes	Present if ever recorded before index date
Health conditions (Yes/No)		
	Rheumatoid arthritis	Present if ever recorded before patient study entry date using medical codes.
	Atrial fibrillation	Present if ever recorded before patient study entry date using medical codes.
	Migraine	Present if ever recorded before patient study entry date using medical codes.
	Systemic lupus erythromatosus	Present if ever recorded before patient study entry date using medical codes.
	Severe mental illness (including psychosis, schizophrenia, or bipolar affective disease)	Present if ever recorded before patient study entry date using medical codes.
	Gravidity	Number of pregnancies recorded in the pregnancy register
Pregnancy complications (Yes/No)	Pre-eclampsia	Present if ever recorded before patient study entry date using medical codes.
	Gestational hypertension	Present if ever recorded before patient study entry date using medical codes.
	Small for gestational age	Present if ever recorded before patient study entry date using medical codes. We also used the INTERGROWTH-21st software to calculate weight centiles and SGA was defined as weight centile below than 10.
	Postnatal depression	Present if ever recorded before patient study entry date using medical codes.
	Gestational diabetes mellitus	Present if ever recorded before patient study entry date using medical codes.
	Stillbirth	Present if ever recorded before patient study entry date using medical codes. We also used the stillbirth outcome flag from pregnancy register.
	Miscarriage	Present if ever recorded before patient study entry date using medical codes. We also used the miscarriage outcome flag from pregnancy register.
	Preterm birth	Present if ever recorded before patient study entry date using medical codes. We also used the preterm birth outcome flag from pregnancy register.
	Placental abruption	Present if ever recorded before patient study entry date using medical codes.
**Outcomes**	Type 2 diabetes mellitus (T2DM)	Incident T2DM was defined using routinely recorded primary care medical codes (SNOMED Clinical Terms) and, where

		available, linked Hospital Episode Statistics ICD-10 codes. Women with a record of T2DM prior to the index date were excluded from the T2DM outcome cohort.
	CKD	Incident CKD was defined as the first recorded diagnosis of CKD stage 3–5 using routinely recorded primary care medical codes (SNOMED Clinical Terms) and, where available, linked Hospital Episode Statistics ICD-10 codes. In addition, CKD was identified using laboratory-based criteria, defined as two estimated glomerular filtration rate (eGFR) measurements <60 mL/min/1.73 m² at least 90 days apart, in line with established clinical guidelines. Women with a record of CKD prior to the index date were excluded from the CKD outcome cohort.
	Hypertension	Incident hypertension was defined using primary care medical codes indicating a diagnosis of hypertension and/or initiation of antihypertensive treatment, consistent with definitions used in established cardiovascular risk prediction models. Women with a record of hypertension prior to the index date were excluded from the hypertension outcome cohort.
	CVD	Incident cardiovascular disease was defined as the first recorded diagnosis of ischaemic heart disease or cerebrovascular disease, including stroke and transient ischaemic attack, identified using primary care medical codes and linked Hospital Episode Statistics ICD-10 codes. Women with a record of CVD prior to the index date were excluded from the CVD outcome cohort.

Github links to medical codes used within the MuM-Predict consortium where this study was carried out.

https://github.com/mumpredict/Exposure-multimorbidity-clinical-codes-
https://github.com/mumpredict/Read-codes-and-ICD-10-codes
https://github.com/mumpredict/Outcome-clinical-code-lists

Baseline characteristics by exposure status

Hypertension outcome cohort

Table 2: Baseline Characteristics by exposure status in the Hypertension cohort

	Miscarriage			Gestational hypertension			Pre-eclampsia		
	**No history**	**Previous**	**Latest**	**No history**	**Previous**	**Latest**	**No history**	**Previous**	**Latest**
n	1188362	124533	76565	1327774	27279	34407	1351129	16233	22098
Mean (sd) age, years	32.69 (6.55)	34.93 (5.72)	35.84 (7.15)	33.05 (6.61)	33.88 (5.64)	33.03 (5.96)	33.05 (6.59)	34.02 (5.67)	33.40 (5.98)
Index of Multiple Deprivation (IMD)									
1	259646 (21.9)	26432 (21.2)	17265 (22.6)	290026 (21.9)	5966 (21.9)	7351 (21.4)	295114 (21.9)	3547 (21.9)	4682 (21.2)
2	254833 (21.4)	24597 (19.8)	16608 (21.7)	283008 (21.3)	5552 (20.4)	7478 (21.7)	287828 (21.3)	3346 (20.6)	4864 (22.0)
3	224792 (18.9)	22940 (18.4)	14371 (18.8)	250436 (18.9)	5079 (18.6)	6588 (19.1)	254915 (18.9)	3011 (18.5)	4177 (18.9)
4	222217 (18.7)	24426 (19.6)	14154 (18.5)	249049 (18.8)	5209 (19.1)	6539 (19.0)	253603 (18.8)	3030 (18.7)	4164 (18.8)
5	224992 (18.9)	25983 (20.9)	13950 (18.2)	253083 (19.1)	5441 (19.9)	6401 (18.6)	257464 (19.1)	3283 (20.2)	4178 (18.9)
Missing IMD	1882 (0.2)	155 (0.1)	217 (0.3)	2172 (0.2)	32 (0.1)	50 (0.1)	2205 (0.2)	16 (0.1)	33 (0.1)
Median (IQR) BMI	24.60 [21.80, 28.73]	25.00 [22.04, 29.30]	24.80 [21.85, 29.20]	24.50 [21.72, 28.69]	27.15 [23.40, 32.30]	27.00 [23.30, 32.00]	24.60 [21.80, 28.70]	27.06 [23.30, 32.03]	27.04 [23.25, 32.00]
Missing BMI	180030 (15.1)	12899 (10.4)	10967 (14.3)	197142 (14.8)	2628 (9.6)	4126 (12.0)	199955 (14.8)	1459 (9.0)	2482 (11.2)
Median (IQR) SBP	115.00 [108.00, 123.00]	116.00 [109.00, 124.00]	118.00 [110.00, 126.00]	115.00 [108.00, 123.00]	120.00 [112.00, 130.00]	122.00 [115.00, 130.00]	115.00 [108.00, 123.00]	120.00 [112.00, 130.00]	122.50 [115.00, 131.00]
Missing SBP	37152 (3.1)	1093 (0.9)	2881 (3.8)	40510 (3.1)	162 (0.6)	454 (1.3)	40808 (3.0)	84 (0.5)	234 (1.1)
Ethnicity									
White	732612 (61.6)	82809 (66.5)	46439 (60.7)	821779 (61.9)	18177 (66.6)	21904 (63.7)	837215 (62.0)	10730 (66.1)	13915 (63.0)
Black	53292 (4.5)	5078 (4.1)	4770 (6.2)	60072 (4.5)	1108 (4.1)	1960 (5.7)	60859 (4.5)	797 (4.9)	1484 (6.7)
South Asian	99753 (8.4)	10211 (8.2)	6577 (8.6)	112066 (8.4)	1836 (6.7)	2639 (7.7)	113494 (8.4)	1211 (7.5)	1836 (8.3)
Mixed ethnicity	17817 (1.5)	1676 (1.3)	1424 (1.9)	20139 (1.5)	289 (1.1)	489 (1.4)	20369 (1.5)	204 (1.3)	344 (1.6)
Other ethnicity	13996 (1.2)	1304 (1.0)	974 (1.3)	15749 (1.2)	205 (0.8)	320 (0.9)	15933 (1.2)	133 (0.8)	208 (0.9)
Missing ethnicity	270892 (22.8)	23455 (18.8)	16381 (21.4)	297969 (22.4)	5664 (20.8)	7095 (20.6)	303259 (22.4)	3158 (19.5)	4311 (19.5)

Smoking status									
Non-smoker	634706 (53.4)	70626 (56.7)	39009 (51.0)	708095 (53.4)	16313 (59.8)	19933 (58.0)	721284 (53.4)	9949 (61.3)	13108 (59.3)
Current smoker	274152 (23.1)	26459 (21.3)	20361 (26.6)	310110 (23.4)	4951 (18.2)	5911 (17.2)	314475 (23.3)	2800 (17.3)	3697 (16.7)
Ex-smoker	191004 (16.1)	23305 (18.7)	12114 (15.8)	215075 (16.2)	4983 (18.3)	6365 (18.5)	219504 (16.3)	2898 (17.9)	4021 (18.2)
Missing smoking status	88067 (7.4)	4088 (3.3)	4947 (6.5)	93900 (7.1)	1021 (3.7)	2181 (6.3)	95259 (7.1)	582 (3.6)	1261 (5.7)
Alcohol misuse	20704 (1.7)	2173 (1.7)	1739 (2.3)	23748 (1.8)	403 (1.5)	465 (1.4)	24068 (1.8)	227 (1.4)	321 (1.5)
Family history of CVD	80379 (6.8)	9480 (7.6)	6060 (7.9)	90908 (6.8)	2273 (8.3)	2738 (8.0)	92778 (6.9)	1383 (8.5)	1758 (8.0)
Median (IQR) Gravidity	2.00 [2.00, 4.00]	4.00 [3.00, 6.00]	3.00 [2.00, 5.00]	3.00 [2.00, 4.00]	3.00 [2.00, 5.00]	2.00 [1.00, 3.00]	3.00 [2.00, 4.00]	3.00 [2.00, 5.00]	2.00 [1.00, 3.00]
Gravidity									
1	293494 (24.7)	0 (0.0)	16476 (21.5)	297797 (22.4)	0 (0.0)	12173 (35.4)	302276 (22.4)	0 (0.0)	7694 (34.8)
2	304945 (25.7)	13432 (10.8)	13844 (18.1)	316595 (23.8)	7252 (26.6)	8374 (24.3)	322519 (23.9)	4324 (26.6)	5378 (24.3)
3	227702 (19.2)	27564 (22.1)	13457 (17.6)	256399 (19.3)	6485 (23.8)	5839 (17.0)	261162 (19.3)	3835 (23.6)	3726 (16.9)
4	148856 (12.5)	25571 (20.5)	10782 (14.1)	176707 (13.3)	5049 (18.5)	3453 (10.0)	179928 (13.3)	3005 (18.5)	2276 (10.3)
5+	213365 (18.0)	57966 (46.5)	22006 (28.7)	280276 (21.1)	8493 (31.1)	4568 (13.3)	285244 (21.1)	5069 (31.2)	3024 (13.7)
Medications									
Statins	1762 (0.1)	157 (0.1)	121 (0.2)	1947 (0.1)	43 (0.2)	50 (0.1)	1970 (0.1)	32 (0.2)	38 (0.2)
Corticosteroids	3175 (0.3)	431 (0.3)	298 (0.4)	3699 (0.3)	94 (0.3)	111 (0.3)	3770 (0.3)	58 (0.4)	76 (0.3)
Antipsycotics	3855 (0.3)	424 (0.3)	342 (0.4)	4434 (0.3)	91 (0.3)	96 (0.3)	4502 (0.3)	53 (0.3)	66 (0.3)
Health conditions									
Type 1 diabetes	4517 (0.4)	558 (0.4)	359 (0.5)	4820 (0.4)	258 (0.9)	356 (1.0)	4942 (0.4)	201 (1.2)	291 (1.3)
Venous thromboembolism	7662 (0.6)	1069 (0.9)	581 (0.8)	8822 (0.7)	248 (0.9)	242 (0.7)	9004 (0.7)	154 (0.9)	154 (0.7)
Migraine	125390 (10.6)	16193 (13.0)	8954 (11.7)	142304 (10.7)	3931 (14.4)	4302 (12.5)	145264 (10.8)	2405 (14.8)	2868 (13.0)
Rheumatoid arthritis	4003 (0.3)	506 (0.4)	319 (0.4)	4567 (0.3)	116 (0.4)	145 (0.4)	4634 (0.3)	89 (0.5)	105 (0.5)
SLE	1676 (0.1)	272 (0.2)	155 (0.2)	1951 (0.1)	66 (0.2)	86 (0.2)	1984 (0.1)	49 (0.3)	70 (0.3)
Severe mental illness	8978 (0.8)	1108 (0.9)	788 (1.0)	10373 (0.8)	242 (0.9)	259 (0.8)	10557 (0.8)	143 (0.9)	174 (0.8)
Atrial fibrillation	929 (0.1)	148 (0.1)	97 (0.1)	1105 (0.1)	31 (0.1)	38 (0.1)	1128 (0.1)	17 (0.1)	29 (0.1)
Learning disability	3997 (0.3)	207 (0.2)	147 (0.2)	4226 (0.3)	45 (0.2)	80 (0.2)	4262 (0.3)	27 (0.2)	62 (0.3)
CVD	6981 (0.6)	845 (0.7)	570 (0.7)	7885 (0.6)	213 (0.8)	298 (0.9)	8041 (0.6)	139 (0.9)	216 (1.0)

T2DM	9409 (0.8)	1262 (1.0)	897 (1.2)	10666 (0.8)	437 (1.6)	465 (1.4)	10889 (0.8)	310 (1.9)	369 (1.7)
CKD	694 (0.1)	63 (0.1)	57 (0.1)	739 (0.1)	22 (0.1)	53 (0.2)	751 (0.1)	19 (0.1)	44 (0.2)
Hypertension outcomes									
Outcomes	41455 (3.5)	4084 (3.3)	3694 (4.8)	42925 (3.2)	2280 (8.4)	4028 (11.7)	44838 (3.3)	1458 (9.0)	2937 (13.3)

Baseline demographic, socioeconomic, lifestyle, clinical, reproductive, medication, and comorbidity characteristics of women included in the hypertension outcome cohort, stratified by history of pregnancy complications. Exposure categories are presented as *no history*, *previous history*, or *latest pregnancy* affected for each complication. Continuous variables are reported as mean (standard deviation) or median (interquartile range), and categorical variables as number (percentage). Outcomes represent incident hypertension during follow-up. **Abbreviations:** BMI, body mass index; CKD, chronic kidney disease; CVD, cardiovascular disease; GDM, gestational diabetes mellitus; IMD, Index of Multiple Deprivation; IQR, interquartile range; SBP, systolic blood pressure; SGA, small for gestational age; SLE, systemic lupus erythematosus; T2DM, type 2 diabetes mellitus.

Table 2 (continued): Baseline Characteristics by exposure status in the Hypertension cohort

	GDM			Placental abruption			Stillbirth		
	**No history**	**Previous**	**Latest**	**No history**	**Previous**	**Latest**	**No history**	**Previous**	**Latest**
n	1323427	17578	32975	1380102	5416	3942	1375223	11101	3136
Mean (sd) age, years	32.96 (6.59)	35.81 (5.38)	34.38 (5.22)	33.07 (6.58)	34.40 (5.71)	31.52 (6.18)	33.05 (6.58)	35.05 (5.82)	31.80 (6.82)
Index of Multiple Deprivation (IMD)									
1	285934 (21.6)	4491 (25.6)	8171 (24.8)	300621 (21.8)	1620 (29.9)	1102 (28.0)	299119 (21.8)	3226 (29.1)	998 (31.8)
2	280342 (21.2)	4074 (23.2)	7707 (23.4)	294057 (21.3)	1140 (21.0)	841 (21.3)	292671 (21.3)	2565 (23.1)	802 (25.6)
3	250244 (18.9)	3163 (18.0)	6109 (18.5)	260475 (18.9)	916 (16.9)	712 (18.1)	259696 (18.9)	1885 (17.0)	522 (16.6)
4	250023 (18.9)	2965 (16.9)	5557 (16.9)	259238 (18.8)	904 (16.7)	655 (16.6)	258608 (18.8)	1770 (15.9)	419 (13.4)
5	254714 (19.2)	2860 (16.3)	5391 (16.3)	263470 (19.1)	830 (15.3)	625 (15.9)	262893 (19.1)	1642 (14.8)	390 (12.4)
Missing IMD	2170 (0.2)	25 (0.1)	40 (0.1)	2241 (0.2)	6 (0.1)	7 (0.2)	2236 (0.2)	13 (0.1)	5 (0.2)
Median (IQR) BMI	24.50 [21.70, 28.50]	28.40 [24.20, 33.70]	28.66 [24.40, 33.79]	24.60 [21.80, 28.80]	24.98 [21.90, 29.30]	24.30 [21.50, 28.40]	24.60 [21.80, 28.80]	26.19 [22.70, 30.80]	25.46 [22.10, 30.10]
Missing BMI	199561 (15.1)	1152 (6.6)	2640 (8.0)	202531 (14.7)	746 (13.8)	619 (15.7)	202012 (14.7)	1357 (12.2)	527 (16.8)
Median (IQR) SBP	115.00 [108.00, 123.00]	118.00 [110.00, 126.00]	118.00 [110.00, 126.00]	116.00 [108.00, 123.00]	116.00 [108.00, 124.00]	115.00 [108.00, 122.00]	116.00 [108.00, 123.00]	117.00 [109.00, 125.00]	116.00 [109.00, 124.00]

Missing SBP	40558 (3.1)	96 (0.5)	385 (1.2)	40919 (3.0)	89 (1.6)	118 (3.0)	40751 (3.0)	229 (2.1)	146 (4.7)
Ethnicity									
White	825175 (62.4)	9796 (55.7)	18164 (55.1)	856140 (62.0)	3422 (63.2)	2298 (58.3)	853946 (62.1)	6389 (57.6)	1525 (48.6)
Black	58761 (4.4)	1037 (5.9)	2043 (6.2)	62661 (4.5)	280 (5.2)	199 (5.0)	61974 (4.5)	870 (7.8)	296 (9.4)
South Asian	102458 (7.7)	3787 (21.5)	7175 (21.8)	115704 (8.4)	513 (9.5)	324 (8.2)	114780 (8.3)	1409 (12.7)	352 (11.2)
Mixed ethnicity	19682 (1.5)	313 (1.8)	630 (1.9)	20767 (1.5)	87 (1.6)	63 (1.6)	20662 (1.5)	204 (1.8)	51 (1.6)
Other ethnicity	15100 (1.1)	327 (1.9)	642 (1.9)	16170 (1.2)	65 (1.2)	39 (1.0)	16065 (1.2)	177 (1.6)	32 (1.0)
Missing ethnicity	302251 (22.8)	2318 (13.2)	4321 (13.1)	308660 (22.4)	1049 (19.4)	1019 (25.8)	307796 (22.4)	2052 (18.5)	880 (28.1)
Smoking status									
Non-smoker	702786 (53.1)	11386 (64.8)	21050 (63.9)	739843 (53.6)	2625 (48.5)	1873 (47.5)	736922 (53.6)	5852 (52.7)	1567 (50.0)
Current smoker	309002 (23.4)	2953 (16.8)	5472 (16.6)	318108 (23.1)	1678 (31.0)	1186 (30.1)	317201 (23.1)	2856 (25.7)	915 (29.2)
Ex-smoker	215515 (16.3)	2933 (16.7)	5612 (17.0)	224943 (16.3)	868 (16.0)	612 (15.5)	224291 (16.3)	1777 (16.0)	355 (11.3)
Missing smoking status	95533 (7.2)	290 (1.7)	830 (2.5)	96589 (7.0)	243 (4.5)	270 (6.9)	96194 (7.0)	610 (5.5)	298 (9.5)
Alcohol misuse	23147 (1.7)	351 (2.0)	601 (1.8)	24428 (1.8)	131 (2.4)	57 (1.4)	24346 (1.8)	213 (1.9)	57 (1.8)
Family history of CVD	89994 (6.8)	1552 (8.8)	2797 (8.5)	95259 (6.9)	393 (7.3)	267 (6.8)	94840 (6.9)	870 (7.8)	209 (6.7)
Median (IQR) Gravidity	3.00 [2.00, 4.00]	4.00 [3.00, 6.00]	3.00 [2.00, 4.00]	3.00 [2.00, 4.00]	4.00 [3.00, 6.00]	3.00 [2.00, 4.00]	3.00 [2.00, 4.00]	5.00 [4.00, 7.00]	3.00 [2.00, 5.00]
Gravidity									
1	300491 (22.7)	0 (0.0)	6521 (19.8)	308976 (22.4)	95 (1.8)	899 (22.8)	309222 (22.5)	80 (0.7)	668 (21.3)
2	318446 (24.1)	2650 (15.1)	8257 (25.0)	330467 (23.9)	815 (15.0)	939 (23.8)	330685 (24.0)	890 (8.0)	646 (20.6)
3	255555 (19.3)	3669 (20.9)	6785 (20.6)	266996 (19.3)	1006 (18.6)	721 (18.3)	266452 (19.4)	1698 (15.3)	573 (18.3)
4	175203 (13.2)	3481 (19.8)	4397 (13.3)	183802 (13.3)	913 (16.9)	494 (12.5)	182812 (13.3)	2008 (18.1)	389 (12.4)
5+	273732 (20.7)	7778 (44.2)	7015 (21.3)	289861 (21.0)	2587 (47.8)	889 (22.6)	286052 (20.8)	6425 (57.9)	860 (27.4)
Medications									
Statins	907 (0.1)	18 (0.1)	33 (0.1)	2024 (0.1)	15 (0.3)	1 (0.0)	1982 (0.1)	52 (0.5)	6 (0.2)
Corticosteroids	3581 (0.3)	61 (0.3)	136 (0.4)	3866 (0.3)	28 (0.5)	10 (0.3)	3838 (0.3)	50 (0.5)	16 (0.5)
Antipsycotics	4152 (0.3)	90 (0.5)	151 (0.5)	4584 (0.3)	23 (0.4)	14 (0.4)	4560 (0.3)	48 (0.4)	13 (0.4)
Health conditions									
Type 1 diabetes	1323427 (100.0)	17578 (100.0)	32975 (100.0)	5375 (0.4)	32 (0.6)	27 (0.7)	5253 (0.4)	143 (1.3)	38 (1.2)

Venous thromboembolism	8680 (0.7)	151 (0.9)	275 (0.8)	9197 (0.7)	75 (1.4)	40 (1.0)	9114 (0.7)	163 (1.5)	35 (1.1)
Migraine	142071 (10.7)	2451 (13.9)	4055 (12.3)	149374 (10.8)	730 (13.5)	433 (11.0)	148944 (10.8)	1275 (11.5)	318 (10.1)
Rheumatoid arthritis	4410 (0.3)	74 (0.4)	182 (0.6)	4794 (0.3)	22 (0.4)	12 (0.3)	4761 (0.3)	59 (0.5)	8 (0.3)
SLE	1993 (0.2)	24 (0.1)	45 (0.1)	2081 (0.2)	13 (0.2)	9 (0.2)	2042 (0.1)	52 (0.5)	9 (0.3)
Severe mental illness	9987 (0.8)	202 (1.1)	338 (1.0)	10778 (0.8)	71 (1.3)	25 (0.6)	10724 (0.8)	117 (1.1)	33 (1.1)
Atrial fibrillation	1090 (0.1)	15 (0.1)	30 (0.1)	1164 (0.1)	6 (0.1)	4 (0.1)	1158 (0.1)	13 (0.1)	3 (0.1)
Learning disability	4064 (0.3)	50 (0.3)	78 (0.2)	4317 (0.3)	24 (0.4)	10 (0.3)	4297 (0.3)	40 (0.4)	14 (0.4)
CVD	7756 (0.6)	128 (0.7)	230 (0.7)	8331 (0.6)	49 (0.9)	16 (0.4)	8282 (0.6)	96 (0.9)	18 (0.6)
T2DM	1323427 (100.0)	17578 (100.0)	32975 (100.0)	11476 (0.8)	65 (1.2)	27 (0.7)	11202 (0.8)	309 (2.8)	57 (1.8)
CKD	714 (0.1)	12 (0.1)	16 (0.0)	808 (0.1)	5 (0.1)	1 (0.0)	803 (0.1)	6 (0.1)	5 (0.2)
Hypertension outcomes									
Outcomes	44824 (3.4)	793 (4.5)	1394 (4.2)	48872 (3.5)	226 (4.2)	135 (3.4)	48470 (3.5)	645 (5.8)	118 (3.8)

Baseline demographic, socioeconomic, lifestyle, clinical, reproductive, medication, and comorbidity characteristics of women included in the hypertension outcome cohort, stratified by history of pregnancy complications. Exposure categories are presented as *no history*, *previous history*, or *latest pregnancy* affected for each complication. Continuous variables are reported as mean (standard deviation) or median (interquartile range), and categorical variables as number (percentage). Outcomes represent incident hypertension during follow-up. **Abbreviations:** BMI, body mass index; CKD, chronic kidney disease; CVD, cardiovascular disease; GDM, gestational diabetes mellitus; IMD, Index of Multiple Deprivation; IQR, interquartile range; SBP, systolic blood pressure; SGA, small for gestational age; SLE, systemic lupus erythematosus; T2DM, type 2 diabetes mellitus.

Table 2 (continued): Baseline Characteristics by exposure status in the Hypertension cohort

	Preterm birth			SGA			Postnatal depression		
	**No history**	**Previous**	**Latest**	**No history**	**Previous**	**Latest**	**No history**	**Previous**	**Latest**
n	1342122	24633	22705	1334625	21211	33624	1163203	44731	181526
Mean (sd) age, years	33.11 (6.58)	32.52 (6.25)	31.33 (6.22)	33.11 (6.59)	33.56 (5.67)	31.07 (6.25)	33.04 (6.52)	33.43 (6.12)	33.17 (7.05)
Index of Multiple Deprivation (IMD)									
1	289921 (21.6)	6954 (28.2)	6468 (28.5)	286177 (21.5)	6893 (32.5)	10273 (30.6)	240745 (20.7)	12985 (29.0)	49613 (27.3)
2	285464 (21.3)	5456 (22.1)	5118 (22.5)	283188 (21.2)	4851 (22.9)	7999 (23.8)	246668 (21.2)	9924 (22.2)	39446 (21.7)
3	253572 (18.9)	4348 (17.7)	4183 (18.4)	252697 (18.9)	3492 (16.5)	5914 (17.6)	221372 (19.0)	7973 (17.8)	32758 (18.0)

4	253142 (18.9)	4024 (16.3)	3631 (16.0)	252898 (18.9)	3064 (14.4)	4835 (14.4)	222516 (19.1)	7260 (16.2)	31021 (17.1)
5	257843 (19.2)	3822 (15.5)	3260 (14.4)	257498 (19.3)	2885 (13.6)	4542 (13.5)	230039 (19.8)	6524 (14.6)	28362 (15.6)
Missing IMD	2180 (0.2)	29 (0.1)	45 (0.2)	2167 (0.2)	26 (0.1)	61 (0.2)	1863 (0.2)	65 (0.1)	326 (0.2)
Median (IQR) BMI	24.60 [21.80, 28.80]	25.00 [21.87, 29.40]	24.60 [21.51, 28.96]	24.70 [21.80, 28.90]	24.50 [21.50, 28.70]	23.62 [20.90, 27.50]	24.50 [21.70, 28.50]	25.70 [22.30, 30.40]	25.50 [22.10, 30.50]
Missing BMI	198938 (14.8)	2367 (9.6)	2591 (11.4)	196222 (14.7)	2254 (10.6)	5420 (16.1)	177040 (15.2)	4412 (9.9)	22444 (12.4)
Median (IQR) SBP	116.00 [108.00, 123.00]	116.00 [108.00, 124.00]	115.00 [107.00, 122.00]	116.00 [108.00, 123.00]	115.00 [108.00, 123.00]	114.00 [107.00, 122.00]	115.00 [108.00, 123.00]	116.00 [109.00, 124.00]	117.00 [110.00, 124.00]
Missing SBP	40338 (3.0)	368 (1.5)	420 (1.8)	39830 (3.0)	196 (0.9)	1100 (3.3)	37554 (3.2)	271 (0.6)	3301 (1.8)
Ethnicity									
White	831516 (62.0)	16271 (66.1)	14073 (62.0)	832741 (62.4)	11920 (56.2)	17199 (51.2)	701661 (60.3)	30651 (68.5)	129548 (71.4)
Black	60748 (4.5)	1229 (5.0)	1163 (5.1)	60300 (4.5)	982 (4.6)	1858 (5.5)	58076 (5.0)	1312 (2.9)	3752 (2.1)
South Asian	111975 (8.3)	2397 (9.7)	2169 (9.6)	107776 (8.1)	3622 (17.1)	5143 (15.3)	107317 (9.2)	2469 (5.5)	6755 (3.7)
Mixed ethnicity	20084 (1.5)	434 (1.8)	399 (1.8)	20059 (1.5)	310 (1.5)	548 (1.6)	18020 (1.5)	567 (1.3)	2330 (1.3)
Other ethnicity	15704 (1.2)	289 (1.2)	281 (1.2)	15656 (1.2)	241 (1.1)	377 (1.1)	14778 (1.3)	314 (0.7)	1182 (0.7)
Missing ethnicity	302095 (22.5)	4013 (16.3)	4620 (20.3)	298093 (22.3)	4136 (19.5)	8499 (25.3)	263351 (22.6)	9418 (21.1)	37959 (20.9)
Smoking status									
Non-smoker	719205 (53.6)	13151 (53.4)	11985 (52.8)	717559 (53.8)	10768 (50.8)	16014 (47.7)	653522 (56.2)	18830 (42.1)	71989 (39.7)
Current smoker	307849 (22.9)	6773 (27.5)	6350 (28.0)	303060 (22.7)	6845 (32.3)	11067 (32.9)	238411 (20.5)	15849 (35.5)	66712 (36.8)
Ex-smoker	218920 (16.3)	3943 (16.0)	3560 (15.7)	219407 (16.4)	2945 (13.9)	4071 (12.1)	183147 (15.8)	9063 (20.3)	34213 (18.9)
Missing smoking status	95556 (7.1)	753 (3.1)	793 (3.5)	94007 (7.0)	642 (3.0)	2453 (7.3)	87608 (7.5)	963 (2.2)	8531 (4.7)
Alcohol misuse	23457 (1.7)	631 (2.6)	528 (2.3)	23409 (1.8)	497 (2.3)	710 (2.1)	15267 (1.3)	1292 (2.9)	8057 (4.4)
Family history of CVD	92449 (6.9)	1840 (7.5)	1630 (7.2)	92165 (6.9)	1552 (7.3)	2202 (6.5)	77388 (6.7)	3510 (7.8)	15021 (8.3)
Median (IQR) Gravidity	3.00 [2.00, 4.00]	4.00 [2.00, 5.00]	3.00 [2.00, 4.00]	3.00 [2.00, 4.00]	4.00 [3.00, 6.00]	2.00 [1.00, 4.00]	3.00 [2.00, 4.00]	5.00 [3.00, 7.00]	3.00 [2.00, 5.00]
Gravidity									
1	302470 (22.5)	1977 (8.0)	5523 (24.3)	299953 (22.5)	0 (0.0)	10017 (29.8)	275418 (23.7)	0 (0.0)	34552 (19.0)
2	322279 (24.0)	4741 (19.2)	5201 (22.9)	320045 (24.0)	4159 (19.6)	8017 (23.8)	291796 (25.1)	5490 (12.3)	34935 (19.2)
3	259662 (19.3)	4830 (19.6)	4231 (18.6)	258324 (19.4)	4421 (20.8)	5978 (17.8)	228300 (19.6)	7712 (17.2)	32711 (18.0)
4	178574 (13.3)	3858 (15.7)	2777 (12.2)	177550 (13.3)	3931 (18.5)	3728 (11.1)	151031 (13.0)	7959 (17.8)	26219 (14.4)

5+	279137 (20.8)	9227 (37.5)	4973 (21.9)	278753 (20.9)	8700 (41.0)	5884 (17.5)	216658 (18.6)	23570 (52.7)	53109 (29.3)
Medications									
Statins	1971 (0.1)	51 (0.2)	18 (0.1)	1987 (0.1)	35 (0.2)	18 (0.1)	1217 (0.1)	83 (0.2)	740 (0.4)
Corticosteroids	3724 (0.3)	91 (0.4)	89 (0.4)	3684 (0.3)	104 (0.5)	116 (0.3)	2659 (0.2)	168 (0.4)	1077 (0.6)
Antipsycotics	4370 (0.3)	155 (0.6)	96 (0.4)	4438 (0.3)	95 (0.4)	88 (0.3)	1027 (0.1)	172 (0.4)	3422 (1.9)
Health conditions									
Type 1 diabetes	5032 (0.4)	180 (0.7)	222 (1.0)	5355 (0.4)	44 (0.2)	35 (0.1)	4075 (0.4)	251 (0.6)	1108 (0.6)
Venous thromboembolism	8869 (0.7)	267 (1.1)	176 (0.8)	8864 (0.7)	223 (1.1)	225 (0.7)	6943 (0.6)	487 (1.1)	1882 (1.0)
Migraine	144370 (10.8)	3436 (13.9)	2731 (12.0)	144471 (10.8)	2742 (12.9)	3324 (9.9)	111041 (9.5)	8097 (18.1)	31399 (17.3)
Rheumatoid arthritis	4615 (0.3)	116 (0.5)	97 (0.4)	4591 (0.3)	110 (0.5)	127 (0.4)	3669 (0.3)	206 (0.5)	953 (0.5)
SLE	1999 (0.1)	50 (0.2)	54 (0.2)	1990 (0.1)	60 (0.3)	53 (0.2)	1612 (0.1)	102 (0.2)	389 (0.2)
Severe mental illness	10399 (0.8)	289 (1.2)	186 (0.8)	10370 (0.8)	226 (1.1)	278 (0.8)	4504 (0.4)	894 (2.0)	5476 (3.0)
Atrial fibrillation	1135 (0.1)	25 (0.1)	14 (0.1)	1131 (0.1)	22 (0.1)	21 (0.1)	834 (0.1)	61 (0.1)	279 (0.2)
Learning disability	4075 (0.3)	191 (0.8)	85 (0.4)	4200 (0.3)	43 (0.2)	108 (0.3)	2735 (0.2)	205 (0.5)	1411 (0.8)
CVD	8000 (0.6)	243 (1.0)	153 (0.7)	8040 (0.6)	160 (0.8)	196 (0.6)	6063 (0.5)	408 (0.9)	1925 (1.1)
T2DM	10888 (0.8)	399 (1.6)	281 (1.2)	11199 (0.8)	193 (0.9)	176 (0.5)	8443 (0.7)	559 (1.2)	2566 (1.4)
CKD	765 (0.1)	29 (0.1)	20 (0.1)	787 (0.1)	12 (0.1)	15 (0.0)	582 (0.1)	32 (0.1)	200 (0.1)
Hypertension outcomes									
Outcomes	48098 (3.6)	674 (2.7)	461 (2.0)	47430 (3.6)	778 (3.7)	1025 (3.0)	39042 (3.4)	1687 (3.8)	8504 (4.7)

Baseline demographic, socioeconomic, lifestyle, clinical, reproductive, medication, and comorbidity characteristics of women included in the hypertension outcome cohort, stratified by history of pregnancy complications. Exposure categories are presented as *no history*, *previous history*, or *latest pregnancy* affected for each complication. Continuous variables are reported as mean (standard deviation) or median (interquartile range), and categorical variables as number (percentage). Outcomes represent incident hypertension during follow-up. **Abbreviations:** BMI, body mass index; CKD, chronic kidney disease; CVD, cardiovascular disease; GDM, gestational diabetes mellitus; IMD, Index of Multiple Deprivation; IQR, interquartile range; SBP, systolic blood pressure; SGA, small for gestational age; SLE, systemic lupus erythematosus; T2DM, type 2 diabetes mellitus.

Type 2 diabetes outcome cohort

Table 3: Baseline Characteristics by exposure status in the T2DM cohort

	Miscarriage			Gestational hypertension			Pre-eclampsia		
	No history	Previous	Latest	No history	Previous	Latest	No history	Previous	Latest
n	1206838	126825	78220	1332890	30810	48183	1358997	18744	34142
Mean (sd) age, years	32.78 (6.57)	35 (5.73)	35.96 (7.15)	33.09 (6.62)	34.23 (5.72)	34.39 (6.30)	33.09 (6.61)	34.47 (5.75)	35.05 (6.28)
Index of Multiple Deprivation (IMD)									
1	264217 (21.9)	26968 (21.3)	17685 (22.6)	290839 (21.8)	6816 (22.1)	11215 (23.3)	296580 (21.8)	4163 (22.2)	8127 (23.8)
2	259015 (21.5)	25122 (19.8)	17017 (21.8)	283859 (21.3)	6365 (20.7)	10930 (22.7)	289311 (21.3)	3920 (20.9)	7923 (23.2)
3	228312 (18.9)	23373 (18.4)	14658 (18.7)	251555 (18.9)	5734 (18.6)	9054 (18.8)	256570 (18.9)	3464 (18.5)	6309 (18.5)
4	225424 (18.7)	24825 (19.6)	14432 (18.5)	250205 (18.8)	5827 (18.9)	8649 (18.0)	255247 (18.8)	3479 (18.6)	5955 (17.4)
5	227961 (18.9)	26380 (20.8)	14208 (18.2)	254253 (19.1)	6030 (19.6)	8266 (17.2)	259076 (19.1)	3694 (19.7)	5779 (16.9)
Missing IMD	1909 (0.2)	157 (0.1)	220 (0.3)	2179 (0.2)	38 (0.1)	69 (0.1)	2213 (0.2)	24 (0.1)	49 (0.1)
Median (IQR) BMI	24.60 [21.80, 28.90]	25 [22.10, 29.38]	24.80 [21.90, 29.30]	24.50 [21.73, 28.70]	27.40 [23.50, 32.60]	27.80 [23.80, 32.90]	24.60 [21.80, 28.70]	27.40 [23.50, 32.50]	28.09 [24, 33.20]
Missing BMI	182546 (15.1)	13115 (10.3)	11143 (14.2)	198615 (14.9)	2910 (9.4)	5279 (11.0)	201713 (14.8)	1658 (8.8)	3433 (10.1)
Median (IQR) SBP	116 [108, 124]	116 [109, 124]	118 [110, 126]	115 [108, 123]	122 [114, 130]	126 [118, 135]	115 [108, 123]	122 [114, 131]	127 [119, 136]
Missing SBP	37299 (3.1)	1099 (0.9)	2892 (3.7)	40606 (3.0)	176 (0.6)	508 (1.1)	40919 (3.0)	95 (0.5)	276 (0.8)
Ethnicity									
White	744159 (61.7)	84278 (66.5)	47309 (60.5)	826089 (62.0)	20434 (66.3)	29223 (60.7)	843376 (62.1)	12278 (65.5)	20092 (58.8)
Black	56150 (4.7)	5472 (4.3)	5194 (6.6)	60620 (4.5)	1467 (4.8)	4729 (9.8)	61591 (4.5)	1100 (5.9)	4125 (12.1)
South Asian	99820 (8.3)	10206 (8.0)	6626 (8.5)	110631 (8.3)	2097 (6.8)	3924 (8.1)	112237 (8.3)	1407 (7.5)	3008 (8.8)
Mixed ethnicity	18203 (1.5)	1716 (1.4)	1447 (1.8)	20217 (1.5)	329 (1.1)	820 (1.7)	20477 (1.5)	233 (1.2)	656 (1.9)
Other ethnicity	14143 (1.2)	1327 (1.0)	997 (1.3)	15746 (1.2)	240 (0.8)	481 (1.0)	15952 (1.2)	162 (0.9)	353 (1.0)
Missing ethnicity	274363 (22.7)	23826 (18.8)	16647 (21.3)	299587 (22.5)	6243 (20.3)	9006 (18.7)	305364 (22.5)	3564 (19.0)	5908 (17.3)
Smoking status									
Non-smoker	644949 (53.5)	72095 (56.9)	40013 (51.2)	709992 (53.3)	18400 (59.7)	28665 (59.5)	724634 (53.3)	11507 (61.4)	20916 (61.3)
Current smoker	277874 (23.0)	26848 (21.2)	20665 (26.5)	311497 (23.4)	5623 (18.3)	8267 (17.2)	316487 (23.3)	3243 (17.3)	5657 (16.6)

Ex-smoker	194277 (16.1)	23672 (18.7)	12366 (15.8)	216212 (16.2)	5617 (18.2)	8486 (17.6)	221178 (16.3)	3311 (17.7)	5826 (17.1)
Missing smoking status	89296 (7.4)	4152 (3.3)	5040 (6.5)	94592 (7.1)	1157 (3.8)	2739 (5.7)	96087 (7.1)	677 (3.6)	1724 (5.1)
Alcohol misuse	21414 (1.8)	2259 (1.8)	1800 (2.3)	24132 (1.8)	501 (1.6)	840 (1.7)	24540 (1.8)	292 (1.6)	641 (1.9)
Family history of CVD	83191 (6.9)	9780 (7.7)	6314 (8.1)	92037 (6.9)	2765 (9.0)	4483 (9.3)	94262 (6.9)	1734 (9.3)	3289 (9.6)
Median (IQR) Gravidity	2 [2, 4]	4 [3, 6]	3 [2, 5]	3 [2, 4]	4 [2, 5]	2 [1, 4]	3 [2, 4]	4 [3, 5]	2 [1, 4]
Gravidity									
1	296671 (24.6)	0 (0.0)	16716 (21.4)	298636 (22.4)	0 (0.0)	14751 (30.6)	303545 (22.3)	0 (0.0)	9842 (28.8)
2	309149 (25.6)	13630 (10.7)	14133 (18.1)	317693 (23.8)	7825 (25.4)	11394 (23.6)	324308 (23.9)	4666 (24.9)	7938 (23.2)
3	231526 (19.2)	27978 (22.1)	13732 (17.6)	257578 (19.3)	7249 (23.5)	8409 (17.5)	262882 (19.3)	4371 (23.3)	5983 (17.5)
4	151573 (12.6)	25968 (20.5)	11053 (14.1)	177581 (13.3)	5680 (18.4)	5333 (11.1)	181195 (13.3)	3456 (18.4)	3943 (11.5)
5+	217919 (18.1)	59249 (46.7)	22586 (28.9)	281402 (21.1)	10056 (32.6)	8296 (17.2)	287067 (21.1)	6251 (33.3)	6436 (18.9)
Medications									
Statins	1477 (0.1)	101 (0.1)	104 (0.1)	1354 (0.1)	74 (0.2)	254 (0.5)	1405 (0.1)	51 (0.3)	226 (0.7)
Treated hypertension	7961 (0.7)	842 (0.7)	673 (0.9)	4641 (0.3)	693 (2.2)	4142 (8.6)	4978 (0.4)	582 (3.1)	3916 (11.5)
Corticosteroids	3438 (0.3)	466 (0.4)	318 (0.4)	3853 (0.3)	115 (0.4)	254 (0.5)	3938 (0.3)	73 (0.4)	211 (0.6)
Antipsycotics	3964 (0.3)	433 (0.3)	354 (0.5)	4445 (0.3)	118 (0.4)	188 (0.4)	4535 (0.3)	69 (0.4)	147 (0.4)
Health conditions									
Venous thromboembolism	7962 (0.7)	1097 (0.9)	606 (0.8)	8948 (0.7)	296 (1.0)	421 (0.9)	9181 (0.7)	186 (1.0)	298 (0.9)
Migraine	128663 (10.7)	16640 (13.1)	9237 (11.8)	143694 (10.8)	4545 (14.8)	6301 (13.1)	147100 (10.8)	2839 (15.1)	4601 (13.5)
Rheumatoid arthritis	4187 (0.3)	521 (0.4)	330 (0.4)	4613 (0.3)	146 (0.5)	279 (0.6)	4702 (0.3)	111 (0.6)	225 (0.7)
SLE	1882 (0.2)	300 (0.2)	180 (0.2)	2067 (0.2)	86 (0.3)	209 (0.4)	2117 (0.2)	62 (0.3)	183 (0.5)
Severe mental illness	9172 (0.8)	1131 (0.9)	808 (1.0)	10398 (0.8)	292 (0.9)	421 (0.9)	10610 (0.8)	182 (1.0)	319 (0.9)
Atrial fibrillation	1129 (0.1)	164 (0.1)	121 (0.2)	1256 (0.1)	51 (0.2)	107 (0.2)	1298 (0.1)	29 (0.2)	87 (0.3)
Learning disability	4089 (0.3)	215 (0.2)	153 (0.2)	4254 (0.3)	63 (0.2)	140 (0.3)	4302 (0.3)	39 (0.2)	116 (0.3)
Hypertension	29089 (2.4)	3730 (2.9)	2651 (3.4)	17353 (1.3)	4047 (13.1)	14070 (29.2)	20360 (1.5)	2884 (15.4)	12226 (35.8)
CVD	8047 (0.7)	964 (0.8)	684 (0.9)	8578 (0.6)	337 (1.1)	780 (1.6)	8827 (0.6)	230 (1.2)	638 (1.9)
CKD	1226 (0.1)	126 (0.1)	116 (0.1)	987 (0.1)	97 (0.3)	384 (0.8)	1022 (0.1)	88 (0.5)	358 (1.0)
T2DM outcomes and follow-up									

Outcomes	18276 (1.5)	1872 (1.5)	1606 (2.1)	18812 (1.4)	958 (3.1)	1984 (4.1)	19510 (1.4)	628 (3.4)	1616 (4.7)
Median (IQR) Follow-up time, years	4.20 [1.48, 9.72]	4.43 [1.62, 9.21]	4.02 [1.39, 9.32]	4.19 [1.48, 9.65]	4.90 [1.84, 9.82]	4.20 [1.53, 9.47]	4.21 [1.48, 9.66]	4.70 [1.74, 9.43]	4.13 [1.51, 9.20]

Baseline demographic, socioeconomic, lifestyle, clinical, reproductive, medication, and comorbidity characteristics of women included in the type 2 diabetes outcome cohort, stratified by history of pregnancy complications. Exposure categories are presented as *no history*, *previous history*, or *latest pregnancy* affected for each complication. Continuous variables are reported as mean (standard deviation) or median (interquartile range), and categorical variables as number (percentage). Outcomes represent incident type 2 diabetes during follow-up. **Abbreviations:** BMI, body mass index; CKD, chronic kidney disease; CVD, cardiovascular disease; GDM, gestational diabetes mellitus; IMD, Index of Multiple Deprivation; IQR, interquartile range; SBP, systolic blood pressure; SGA, small for gestational age; SLE, systemic lupus erythematosus; T2DM, type 2 diabetes mellitus.

Table 3 (continued): Baseline Characteristics by exposure status in the T2DM cohort

	GDM			Placental abruption			Stillbirth		
	No history	Previous	Latest	No history	Previous	Latest	No history	Previous	Latest
n	1358544	18626	34713	1402208	5636	4039	1397320	11386	3177
Mean (sd) age, years	33.08 (6.63)	35.95 (5.40)	34.51 (5.25)	33.15 (6.60)	34.57 (5.76)	31.64 (6.18)	33.14 (6.60)	35.16 (5.85)	31.95 (6.84)
Index of Multiple Deprivation (IMD)									
1	295346 (21.7)	4824 (25.9)	8700 (25.1)	306075 (21.8)	1675 (29.7)	1120 (27.7)	304560 (21.8)	3307 (29.1)	1003 (31.6)
2	288662 (21.2)	4355 (23.4)	8137 (23.4)	299092 (21.3)	1188 (21.1)	874 (21.6)	297686 (21.3)	2646 (23.2)	822 (25.9)
3	256605 (18.9)	3331 (17.9)	6407 (18.5)	264658 (18.9)	960 (17.0)	725 (17.9)	263880 (18.9)	1934 (17.0)	529 (16.7)
4	255759 (18.8)	3103 (16.7)	5819 (16.8)	263075 (18.8)	938 (16.6)	668 (16.5)	262442 (18.8)	1812 (15.9)	427 (13.4)
5	259953 (19.1)	2986 (16.0)	5610 (16.2)	267036 (19.0)	868 (15.4)	645 (16.0)	266486 (19.1)	1672 (14.7)	391 (12.3)
Missing IMD	2219 (0.2)	27 (0.1)	40 (0.1)	2272 (0.2)	7 (0.1)	7 (0.2)	2266 (0.2)	15 (0.1)	5 (0.2)
Median (IQR) BMI	24.60 [21.79, 28.70]	28.70 [24.40, 34]	28.90 [24.50, 34]	24.70 [21.80, 28.91]	25.04 [22, 29.47]	24.40 [21.50, 28.50]	24.70 [21.80, 28.90]	26.20 [22.70, 30.90]	25.50 [22.10, 30.10]
Missing BMI	202875 (14.9)	1195 (6.4)	2734 (7.9)	205398 (14.6)	777 (13.8)	629 (15.6)	204856 (14.7)	1406 (12.3)	542 (17.1)
Median (IQR) SBP	116 [108, 124]	119 [110, 128]	118 [110, 126]	116 [109, 124]	116 [109, 125]	115 [108, 123]	116 [109, 124]	118 [110, 126]	117 [109, 125]
Missing SBP	40801 (3.0)	97 (0.5)	392 (1.1)	41084 (2.9)	88 (1.6)	118 (2.9)	40912 (2.9)	231 (2.0)	147 (4.6)
Ethnicity									
White	846310 (62.3)	10333 (55.5)	19103 (55.0)	869851 (62.0)	3545 (62.9)	2350 (58.2)	867701 (62.1)	6507 (57.1)	1538 (48.4)

Black	63340 (4.7)	1203 (6.5)	2273 (6.5)	66286 (4.7)	318 (5.6)	212 (5.2)	65530 (4.7)	976 (8.6)	310 (9.8)
South Asian	105170 (7.7)	3993 (21.4)	7489 (21.6)	115792 (8.3)	527 (9.4)	333 (8.2)	114889 (8.2)	1410 (12.4)	353 (11.1)
Mixed ethnicity	20362 (1.5)	334 (1.8)	670 (1.9)	21209 (1.5)	93 (1.7)	64 (1.6)	21101 (1.5)	213 (1.9)	52 (1.6)
Other ethnicity	15450 (1.1)	341 (1.8)	676 (1.9)	16360 (1.2)	67 (1.2)	40 (1.0)	16255 (1.2)	179 (1.6)	33 (1.0)
Missing ethnicity	307912 (22.7)	2422 (13.0)	4502 (13.0)	312710 (22.3)	1086 (19.3)	1040 (25.7)	311844 (22.3)	2101 (18.5)	891 (28.0)
Smoking status									
Non-smoker	722834 (53.2)	12051 (64.8)	22172 (63.9)	752373 (53.7)	2748 (48.8)	1936 (47.9)	749415 (53.7)	6053 (53.2)	1589 (50.0)
Current smoker	316515 (23.3)	3139 (16.9)	5733 (16.5)	322446 (23.0)	1736 (30.8)	1205 (29.8)	321596 (23.0)	2873 (25.2)	918 (28.9)
Ex-smoker	221272 (16.3)	3112 (16.7)	5931 (17.1)	228797 (16.3)	900 (16.0)	618 (15.3)	228131 (16.3)	1823 (16.0)	361 (11.4)
Missing smoking status	97315 (7.2)	307 (1.6)	866 (2.5)	97960 (7.0)	249 (4.4)	279 (6.9)	97550 (7.0)	630 (5.5)	308 (9.7)
Alcohol misuse	24450 (1.8)	379 (2.0)	644 (1.9)	25272 (1.8)	140 (2.5)	61 (1.5)	25195 (1.8)	219 (1.9)	59 (1.9)
Family history of CVD	94583 (7.0)	1703 (9.1)	2999 (8.6)	98585 (7.0)	423 (7.5)	277 (6.9)	98161 (7.0)	905 (7.9)	219 (6.9)
Median (IQR) Gravidity	3 [2, 4]	4 [3, 6]	3 [2, 4]	3 [2, 4]	4 [3, 6]	3 [2, 4]	3 [2, 4]	5 [4, 7]	3 [2, 5]
Gravidity									
1	306568 (22.6)	0 (0.0)	6819 (19.6)	312375 (22.3)	101 (1.8)	911 (22.6)	312631 (22.4)	85 (0.7)	671 (21.1)
2	325497 (24.0)	2767 (14.9)	8648 (24.9)	335117 (23.9)	831 (14.7)	964 (23.9)	335346 (24.0)	902 (7.9)	664 (20.9)
3	262272 (19.3)	3856 (20.7)	7108 (20.5)	271463 (19.4)	1035 (18.4)	738 (18.3)	270897 (19.4)	1759 (15.4)	580 (18.3)
4	180288 (13.3)	3667 (19.7)	4639 (13.4)	187133 (13.3)	955 (16.9)	506 (12.5)	186154 (13.3)	2056 (18.1)	384 (12.1)
5+	283919 (20.9)	8336 (44.8)	7499 (21.6)	296120 (21.1)	2714 (48.2)	920 (22.8)	292292 (20.9)	6584 (57.8)	878 (27.6)
Medications									
Statins	1601 (0.1)	29 (0.2)	52 (0.1)	1671 (0.1)	10 (0.2)	1 (0.0)	1653 (0.1)	22 (0.2)	7 (0.2)
Treated hypertension	8834 (0.7)	247 (1.3)	395 (1.1)	9392 (0.7)	57 (1.0)	27 (0.7)	9297 (0.7)	149 (1.3)	30 (0.9)
Corticosteroids	4001 (0.3)	69 (0.4)	152 (0.4)	4178 (0.3)	32 (0.6)	12 (0.3)	4153 (0.3)	52 (0.5)	17 (0.5)
Antipsycotics	4489 (0.3)	99 (0.5)	163 (0.5)	4714 (0.3)	23 (0.4)	14 (0.3)	4688 (0.3)	49 (0.4)	14 (0.4)
Health conditions									
Venous thromboembolism	9205 (0.7)	160 (0.9)	300 (0.9)	9541 (0.7)	83 (1.5)	41 (1.0)	9462 (0.7)	167 (1.5)	36 (1.1)
Migraine	147563 (10.9)	2638 (14.2)	4339 (12.5)	153327 (10.9)	768 (13.6)	445 (11.0)	152893 (10.9)	1317 (11.6)	330 (10.4)
Rheumatoid arthritis	4761 (0.4)	81 (0.4)	196 (0.6)	5000 (0.4)	24 (0.4)	14 (0.3)	4970 (0.4)	60 (0.5)	8 (0.3)
SLE	2283 (0.2)	25 (0.1)	54 (0.2)	2332 (0.2)	18 (0.3)	12 (0.3)	2299 (0.2)	52 (0.5)	11 (0.3)

Severe mental illness	10530 (0.8)	217 (1.2)	364 (1.0)	11009 (0.8)	75 (1.3)	27 (0.7)	10961 (0.8)	112 (1.0)	38 (1.2)
Atrial fibrillation	1356 (0.1)	20 (0.1)	38 (0.1)	1403 (0.1)	7 (0.1)	4 (0.1)	1394 (0.1)	16 (0.1)	4 (0.1)
Learning disability	4321 (0.3)	54 (0.3)	82 (0.2)	4421 (0.3)	26 (0.5)	10 (0.2)	4402 (0.3)	41 (0.4)	14 (0.4)
Hypertension	32827 (2.4)	1012 (5.4)	1631 (4.7)	35042 (2.5)	294 (5.2)	134 (3.3)	34693 (2.5)	658 (5.8)	119 (3.7)
CVD	9264 (0.7)	149 (0.8)	282 (0.8)	9618 (0.7)	62 (1.1)	15 (0.4)	9541 (0.7)	125 (1.1)	29 (0.9)
CKD	1416 (0.1)	17 (0.1)	35 (0.1)	1455 (0.1)	11 (0.2)	2 (0.0)	1438 (0.1)	22 (0.2)	8 (0.3)
T2DM outcomes and follow-up									
Outcomes	17375 (1.3)	1484 (8.0)	2895 (8.3)	21565 (1.5)	130 (2.3)	59 (1.5)	21300 (1.5)	373 (3.3)	81 (2.5)
Median (IQR) Follow-up time, years	4.31 [1.51, 9.84]	2.66 [1.10, 5.64]	2.58 [1.03, 5.37]	4.21 [1.49, 9.65]	4.40 [1.61, 9.45]	3.13 [1.16, 7.96]	4.21 [1.49, 9.65]	4.46 [1.57, 9.76]	2.56 [0.85, 7.23]

Baseline demographic, socioeconomic, lifestyle, clinical, reproductive, medication, and comorbidity characteristics of women included in the type 2 diabetes outcome cohort, stratified by history of pregnancy complications. Exposure categories are presented as *no history*, *previous history*, or *latest pregnancy* affected for each complication. Continuous variables are reported as mean (standard deviation) or median (interquartile range), and categorical variables as number (percentage). Outcomes represent incident type 2 diabetes during follow-up. **Abbreviations:** BMI, body mass index; CKD, chronic kidney disease; CVD, cardiovascular disease; GDM, gestational diabetes mellitus; IMD, Index of Multiple Deprivation; IQR, interquartile range; SBP, systolic blood pressure; SGA, small for gestational age; SLE, systemic lupus erythematosus; T2DM, type 2 diabetes mellitus.

Table 3 (continued): Baseline Characteristics by exposure status in the T2DM cohort

	Preterm birth			SGA			LGA			Postnatal depression		
	**No history**	**Previous**	**Latest**	**No history**	**Previous**	**Latest**	**No history**	**Previous**	**Latest**	**No history**	**Previous**	**Latest**
n	1363953	25024	22906	1355544	21881	34458	1215416	51660	144807	1180321	45555	186007
Mean (sd) age, years	33.20 (6.61)	32.61 (6.28)	31.41 (6.23)	33.20 (6.61)	33.67 (5.70)	31.17 (6.26)	33.21 (6.73)	34.72 (5.44)	32.15 (5.65)	33.12 (6.53)	33.50 (6.14)	33.32 (7.09)
Index of Multiple Deprivation (IMD)												
1	295232 (21.7)	7094 (28.4)	6544 (28.6)	291277 (21.5)	7092 (32.4)	10501 (30.5)	269022 (22.1)	11474 (22.2)	28374 (19.6)	244731 (20.7)	13227 (29.1)	50912 (27.4)
2	290448 (21.3)	5523 (22.1)	5183 (22.6)	287894 (21.2)	5054 (23.1)	8206 (23.8)	261816 (21.5)	10547 (20.4)	28791 (19.9)	250697 (21.2)	10113 (22.2)	40344 (21.7)
3	257710 (18.9)	4418 (17.7)	4215 (18.4)	256661 (18.9)	3593 (16.4)	6089 (17.7)	229614 (18.9)	9334 (18.1)	27395 (18.9)	224589 (19.0)	8116 (17.8)	33638 (18.1)

4	256969 (18.8)	4077 (16.3)	3635 (15.9)	256565 (18.9)	3160 (14.4)	4956 (14.4)	225819 (18.6)	9894 (19.2)	28968 (20.0)	225520 (19.1)	7394 (16.2)	31767 (17.1)
5	261383 (19.2)	3883 (15.5)	3283 (14.3)	260948 (19.3)	2957 (13.5)	4644 (13.5)	227193 (18.7)	10337 (20.0)	31019 (21.4)	232895 (19.7)	6638 (14.6)	29016 (15.6)
Missing IMD	2211 (0.2)	29 (0.1)	46 (0.2)	2199 (0.2)	25 (0.1)	62 (0.2)	1952 (0.2)	74 (0.1)	260 (0.2)	1889 (0.2)	67 (0.1)	330 (0.2)
Median (IQR) BMI	24.70 [21.80, 28.90]	25 [21.90, 29.60]	24.60 [21.55, 29]	24.70 [21.88, 29]	24.60 [21.50, 28.90]	23.70 [20.94, 27.64]	24.50 [21.70, 28.60]	26.50 [23.10, 31.30]	25.70 [22.60, 30.30]	24.50 [21.79, 28.60]	25.70 [22.30, 30.48]	25.60 [22.20, 30.70]
Missing BMI	201764 (14.8)	2421 (9.7)	2619 (11.4)	198993 (14.7)	2312 (10.6)	5499 (16.0)	181677 (14.9)	5125 (9.9)	20002 (13.8)	179323 (15.2)	4518 (9.9)	22963 (12.3)
Median (IQR) SBP	116 [109, 124]	117 [109, 124]	115 [108, 123]	116 [109, 124]	116 [108, 124]	114 [107, 123]	116 [108, 124]	118 [110, 125]	116 [110, 124]	116 [108, 124]	117 [110, 124]	118 [110, 125]
Missing SBP	40501 (3.0)	367 (1.5)	422 (1.8)	39983 (2.9)	197 (0.9)	1110 (3.2)	37794 (3.1)	446 (0.9)	3050 (2.1)	37695 (3.2)	272 (0.6)	3323 (1.8)
Ethnicity												
White	845071 (62.0)	16517 (66.0)	14158 (61.8)	845791 (62.4)	12286 (56.1)	17669 (51.3)	743910 (61.2)	35715 (69.1)	96121 (66.4)	711712 (60.3)	31219 (68.5)	132815 (71.4)
Black	64264 (4.7)	1326 (5.3)	1226 (5.4)	63761 (4.7)	1080 (4.9)	1975 (5.7)	59938 (4.9)	2006 (3.9)	4872 (3.4)	61316 (5.2)	1415 (3.1)	4085 (2.2)
South Asian	112105 (8.2)	2379 (9.5)	2168 (9.5)	107756 (7.9)	3702 (16.9)	5194 (15.1)	107289 (8.8)	2754 (5.3)	6609 (4.6)	107425 (9.1)	2440 (5.4)	6787 (3.6)
Mixed ethnicity	20534 (1.5)	431 (1.7)	401 (1.8)	20486 (1.5)	318 (1.5)	562 (1.6)	19167 (1.6)	574 (1.1)	1625 (1.1)	18407 (1.6)	589 (1.3)	2370 (1.3)
Other ethnicity	15878 (1.2)	298 (1.2)	291 (1.3)	15826 (1.2)	254 (1.2)	387 (1.1)	14423 (1.2)	571 (1.1)	1473 (1.0)	14952 (1.3)	315 (0.7)	1200 (0.6)
Missing ethnicity	306101 (22.4)	4073 (16.3)	4662 (20.4)	301924 (22.3)	4241 (19.4)	8671 (25.2)	270689 (22.3)	10040 (19.4)	34107 (23.6)	266509 (22.6)	9577 (21.0)	38750 (20.8)
Smoking status												
Non-smoker	731536 (53.7)	13402 (53.6)	12119 (52.9)	729426 (53.8)	11158 (51.0)	16473 (47.8)	645128 (53.1)	29401 (56.9)	82528 (57.0)	663734 (56.3)	19262 (42.3)	74061 (39.8)
Current smoker	312149 (22.9)	6859 (27.4)	6379 (27.9)	307113 (22.7)	7007 (32.0)	11267 (32.7)	291287 (24.0)	9777 (18.9)	24323 (16.8)	241262 (20.4)	16053 (35.3)	68072 (36.6)
Ex-smoker	222747 (16.3)	3982 (15.9)	3586 (15.7)	223065 (16.5)	3055 (14.0)	4195 (12.2)	191432 (15.8)	10436 (20.2)	28447 (19.7)	186004 (15.8)	9234 (20.3)	35077 (18.9)
Missing smoking status	96916 (7.1)	768 (3.1)	804 (3.5)	95334 (7.0)	650 (3.0)	2504 (7.3)	87043 (7.2)	2021 (3.9)	9424 (6.5)	88797 (7.5)	979 (2.2)	8712 (4.7)
Alcohol misuse	24297 (1.8)	636 (2.5)	540 (2.4)	24226 (1.8)	514 (2.3)	733 (2.1)	22885 (1.9)	775 (1.5)	1813 (1.3)	15623 (1.3)	1338 (2.9)	8512 (4.6)
Family history of CVD	95745 (7.0)	1892 (7.6)	1648 (7.2)	95329 (7.0)	1657 (7.6)	2299 (6.7)	85990 (7.1)	3744 (7.2)	9551 (6.6)	79846 (6.8)	3653 (8.0)	15786 (8.5)
Median (IQR) Gravidity	3 [2, 4]	4 [2, 6]	3 [2, 4]	3 [2, 4]	4 [3, 6]	2 [1, 4]	3 [2, 4]	4 [3, 6]	3 [2, 4]	3 [2, 4]	5 [3, 7]	3 [2, 5]

Gravidity												
1	305836 (22.4)	1995 (8.0)	5556 (24.3)	303186 (22.4)	0 (0.0)	10201 (29.6)	286169 (23.5)	0 (0.0)	27218 (18.8)	278220 (23.6)	0 (0.0)	35167 (18.9)
2	326895 (24.0)	4779 (19.1)	5238 (22.9)	324466 (23.9)	4257 (19.5)	8189 (23.8)	289093 (23.8)	7352 (14.2)	40467 (27.9)	295600 (25.0)	5550 (12.2)	35762 (19.2)
3	264050 (19.4)	4925 (19.7)	4261 (18.6)	262539 (19.4)	4566 (20.9)	6131 (17.8)	231280 (19.0)	10444 (20.2)	31512 (21.8)	231838 (19.6)	7850 (17.2)	33548 (18.0)
4	181881 (13.3)	3910 (15.6)	2803 (12.2)	180723 (13.3)	4047 (18.5)	3824 (11.1)	158754 (13.1)	10572 (20.5)	19268 (13.3)	153609 (13.0)	8074 (17.7)	26911 (14.5)
5+	285291 (20.9)	9415 (37.6)	5048 (22.0)	284630 (21.0)	9011 (41.2)	6113 (17.7)	250120 (20.6)	23292 (45.1)	26342 (18.2)	221054 (18.7)	24081 (52.9)	54619 (29.4)
Medications												
Statins	1638 (0.1)	29 (0.1)	15 (0.1)	1632 (0.1)	33 (0.2)	17 (0.0)	1600 (0.1)	37 (0.1)	45 (0.0)	995 (0.1)	59 (0.1)	628 (0.3)
Treated hypertension	9173 (0.7)	196 (0.8)	107 (0.5)	9013 (0.7)	207 (0.9)	256 (0.7)	8707 (0.7)	335 (0.6)	434 (0.3)	6494 (0.6)	355 (0.8)	2627 (1.4)
Corticosteroids	4025 (0.3)	102 (0.4)	95 (0.4)	3974 (0.3)	114 (0.5)	134 (0.4)	3761 (0.3)	176 (0.3)	285 (0.2)	2862 (0.2)	184 (0.4)	1176 (0.6)
Antipsycotics	4498 (0.3)	157 (0.6)	96 (0.4)	4563 (0.3)	96 (0.4)	92 (0.3)	4301 (0.4)	178 (0.3)	272 (0.2)	1045 (0.1)	175 (0.4)	3531 (1.9)
Health conditions												
Venous thromboembolism	9213 (0.7)	277 (1.1)	175 (0.8)	9191 (0.7)	234 (1.1)	240 (0.7)	8305 (0.7)	443 (0.9)	917 (0.6)	7144 (0.6)	501 (1.1)	2020 (1.1)
Migraine	148227 (10.9)	3523 (14.1)	2790 (12.2)	148233 (10.9)	2863 (13.1)	3444 (10.0)	131590 (10.8)	7237 (14.0)	15713 (10.9)	113585 (9.6)	8318 (18.3)	32637 (17.5)
Rheumatoid arthritis	4819 (0.4)	123 (0.5)	96 (0.4)	4792 (0.4)	112 (0.5)	134 (0.4)	4505 (0.4)	174 (0.3)	359 (0.2)	3795 (0.3)	218 (0.5)	1025 (0.6)
SLE	2240 (0.2)	57 (0.2)	65 (0.3)	2228 (0.2)	64 (0.3)	70 (0.2)	2151 (0.2)	71 (0.1)	140 (0.1)	1799 (0.2)	116 (0.3)	447 (0.2)
Severe mental illness	10623 (0.8)	299 (1.2)	189 (0.8)	10592 (0.8)	232 (1.1)	287 (0.8)	9885 (0.8)	473 (0.9)	753 (0.5)	4564 (0.4)	913 (2.0)	5634 (3.0)
Atrial fibrillation	1371 (0.1)	27 (0.1)	16 (0.1)	1355 (0.1)	24 (0.1)	35 (0.1)	1210 (0.1)	68 (0.1)	136 (0.1)	985 (0.1)	70 (0.2)	359 (0.2)
Learning disability	4187 (0.3)	189 (0.8)	81 (0.4)	4305 (0.3)	45 (0.2)	107 (0.3)	4196 (0.3)	67 (0.1)	194 (0.1)	2785 (0.2)	214 (0.5)	1458 (0.8)
Hypertension	34010 (2.5)	854 (3.4)	606 (2.6)	33670 (2.5)	844 (3.9)	956 (2.8)	31668 (2.6)	1408 (2.7)	2394 (1.7)	27063 (2.3)	1438 (3.2)	6969 (3.7)
CVD	9261 (0.7)	270 (1.1)	164 (0.7)	9273 (0.7)	197 (0.9)	225 (0.7)	8559 (0.7)	366 (0.7)	770 (0.5)	6842 (0.6)	469 (1.0)	2384 (1.3)
CKD	1368 (0.1)	59 (0.2)	41 (0.2)	1396 (0.1)	37 (0.2)	35 (0.1)	1388 (0.1)	40 (0.1)	40 (0.0)	1073 (0.1)	64 (0.1)	331 (0.2)
T2DM outcomes and follow-up												

T2DM outcome	21114 (1.5)	383 (1.5)	257 (1.1)	21049 (1.6)	336 (1.5)	369 (1.1)	18134 (1.5)	1300 (2.5)	2320 (1.6)	16848 (1.4)	794 (1.7)	4112 (2.2)
Median (IQR) Follow-up time, years	4.28 [1.50, 9.80]	2.73 [1.08, 5.88]	2.88 [1.14, 5.94]	4.22 [1.49, 9.69]	4.46 [1.69, 8.88]	3.59 [1.33, 8.54]	4.25 [1.49, 9.81]	4.58 [1.69, 9.30]	3.85 [1.40, 8.59]	4.31 [1.51, 9.89]	4.19 [1.55, 8.69]	3.66 [1.32, 8.43]

Baseline demographic, socioeconomic, lifestyle, clinical, reproductive, medication, and comorbidity characteristics of women included in the type 2 diabetes outcome cohort, stratified by history of pregnancy complications. Exposure categories are presented as *no history*, *previous history*, or *latest pregnancy* affected for each complication. Continuous variables are reported as mean (standard deviation) or median (interquartile range), and categorical variables as number (percentage). Outcomes represent incident type 2 diabetes during follow-up. **Abbreviations:** BMI, body mass index; CKD, chronic kidney disease; CVD, cardiovascular disease; GDM, gestational diabetes mellitus; IMD, Index of Multiple Deprivation; IQR, interquartile range; SBP, systolic blood pressure; SGA, small for gestational age; SLE, systemic lupus erythematosus; T2DM, type 2 diabetes mellitus.

Cardiovascular disease outcome cohort

Table 4: Baseline Characteristics by exposure status in the CVD cohort

	Miscarriage	Gestational hypertension	Pre-eclampsia

	No history	Previous	Latest	No history	Previous	Latest	No history	Previous	Latest
n	1215276	127916	79095	1340875	31474	49938	1367284	19256	35747
Mean (sd) age, years	32.82 (6.60)	35.02 (5.73)	35.98 (7.14)	33.12 (6.64)	34.28 (5.74)	34.54 (6.36)	33.13 (6.63)	34.53 (5.77)	35.22 (6.35)
Index of Multiple Deprivation (IMD)									
1	267120 (22.0)	27383 (21.4)	18014 (22.8)	293642 (21.9)	7032 (22.4)	11843 (23.7)	299488 (21.9)	4318 (22.4)	8711 (24.4)
2	261050 (21.5)	25405 (19.9)	17237 (21.8)	285775 (21.3)	6504 (20.7)	11413 (22.9)	291291 (21.3)	4039 (21.0)	8362 (23.4)
3	229653 (18.9)	23512 (18.4)	14833 (18.8)	252821 (18.9)	5862 (18.6)	9315 (18.7)	257882 (18.9)	3562 (18.5)	6554 (18.3)
4	226570 (18.6)	24963 (19.5)	14513 (18.3)	251229 (18.7)	5935 (18.9)	8882 (17.8)	256331 (18.7)	3562 (18.5)	6153 (17.2)
5	228966 (18.8)	26498 (20.7)	14276 (18.0)	255223 (19.0)	6100 (19.4)	8417 (16.9)	260070 (19.0)	3751 (19.5)	5919 (16.6)
Missing IMD	1917 (0.2)	155 (0.1)	222 (0.3)	2185 (0.2)	41 (0.1)	68 (0.1)	2222 (0.2)	24 (0.1)	48 (0.1)
Median (IQR) BMI	24.70 [21.80, 29]	25.10 [22.10, 29.41]	24.90 [21.90, 29.40]	24.60 [21.80, 28.73]	27.50 [23.60, 32.70]	28 [24, 33.20]	24.60 [21.80, 28.81]	27.50 [23.57, 32.70]	28.37 [24.20, 33.50]
Missing BMI	181148 (14.9)	12954 (10.1)	11054 (14.0)	197065 (14.7)	2883 (9.2)	5208 (10.4)	200140 (14.6)	1635 (8.5)	3381 (9.5)
Median (IQR) SBP	116 [109, 124]	116 [109, 124]	118 [110, 127]	115 [108, 123]	122 [114, 130]	126 [118, 135]	116 [108, 123]	122 [114, 131]	127 [119, 136]
Missing SBP	37300 (3.1)	1098 (0.9)	2893 (3.7)	40610 (3.0)	175 (0.6)	506 (1.0)	40922 (3.0)	94 (0.5)	275 (0.8)
Ethnicity									
White	748471 (61.6)	84784 (66.3)	47722 (60.3)	830119 (61.9)	20826 (66.2)	30032 (60.1)	847568 (62.0)	12584 (65.4)	20825 (58.3)
Black	57278 (4.7)	5640 (4.4)	5306 (6.7)	61658 (4.6)	1522 (4.8)	5044 (10.1)	62670 (4.6)	1140 (5.9)	4414 (12.3)
South Asian	102595 (8.4)	10596 (8.3)	6930 (8.8)	113464 (8.5)	2246 (7.1)	4411 (8.8)	115141 (8.4)	1520 (7.9)	3460 (9.7)
Mixed ethnicity	18402 (1.5)	1760 (1.4)	1481 (1.9)	20420 (1.5)	342 (1.1)	881 (1.8)	20692 (1.5)	243 (1.3)	708 (2.0)
Other ethnicity	14282 (1.2)	1341 (1.0)	1004 (1.3)	15889 (1.2)	246 (0.8)	492 (1.0)	16098 (1.2)	167 (0.9)	362 (1.0)
Missing ethnicity	274248 (22.6)	23795 (18.6)	16652 (21.1)	299325 (22.3)	6292 (20.0)	9078 (18.2)	305115 (22.3)	3602 (18.7)	5978 (16.7)
Smoking status									
Non-smoker	650988 (53.6)	72801 (56.9)	40605 (51.4)	715658 (53.4)	18827 (59.8)	29909 (59.9)	730513 (53.5)	11827 (61.4)	22054 (61.7)
Current smoker	279067 (23.0)	27032 (21.1)	20832 (26.4)	312696 (23.3)	5745 (18.3)	8490 (17.0)	317714 (23.2)	3347 (17.4)	5870 (16.4)
Ex-smoker	195250 (16.1)	23866 (18.7)	12478 (15.8)	217126 (16.2)	5722 (18.2)	8746 (17.5)	222142 (16.3)	3394 (17.6)	6058 (17.0)
Missing smoking status	89527 (7.4)	4159 (3.3)	5043 (6.4)	94796 (7.1)	1166 (3.7)	2767 (5.5)	96301 (7.0)	682 (3.5)	1746 (4.9)
Alcohol misuse	21710 (1.8)	2313 (1.8)	1829 (2.3)	24438 (1.8)	524 (1.7)	890 (1.8)	24861 (1.8)	307 (1.6)	684 (1.9)
Family history of CVD	84099 (6.9)	9908 (7.7)	6391 (8.1)	92893 (6.9)	2826 (9.0)	4679 (9.4)	95140 (7.0)	1783 (9.3)	3475 (9.7)

Median (IQR) Gravidity	2 [2, 4]	4 [3, 6]	3 [2, 5]	3 [2, 4]	4 [2, 5]	2 [1, 4]	3 [2, 4]	4 [3, 5]	2 [1, 4]
Gravidity									
1	298497 (24.6)	0 (0.0)	16872 (21.3)	300220 (22.4)	0 (0.0)	15149 (30.3)	305173 (22.3)	0 (0.0)	10196 (28.5)
2	310517 (25.6)	13715 (10.7)	14262 (18.0)	318871 (23.8)	7917 (25.2)	11706 (23.4)	325513 (23.8)	4743 (24.6)	8238 (23.0)
3	232864 (19.2)	28133 (22.0)	13890 (17.6)	258818 (19.3)	7368 (23.4)	8701 (17.4)	264173 (19.3)	4466 (23.2)	6248 (17.5)
4	152755 (12.6)	26150 (20.4)	11151 (14.1)	178689 (13.3)	5804 (18.4)	5563 (11.1)	182364 (13.3)	3550 (18.4)	4142 (11.6)
5+	220643 (18.2)	59918 (46.8)	22920 (29.0)	284277 (21.2)	10385 (33.0)	8819 (17.7)	290061 (21.2)	6497 (33.7)	6923 (19.4)
Medications									
Statins	2906 (0.2)	243 (0.2)	209 (0.3)	2655 (0.2)	154 (0.5)	549 (1.1)	2735 (0.2)	114 (0.6)	509 (1.4)
Treated hypertension	9201 (0.8)	951 (0.7)	777 (1.0)	5416 (0.4)	781 (2.5)	4732 (9.5)	5801 (0.4)	643 (3.3)	4485 (12.5)
Corticosteroids	3523 (0.3)	473 (0.4)	327 (0.4)	3925 (0.3)	117 (0.4)	281 (0.6)	4015 (0.3)	72 (0.4)	236 (0.7)
Antipsycotics	4144 (0.3)	447 (0.3)	364 (0.5)	4628 (0.3)	127 (0.4)	200 (0.4)	4722 (0.3)	77 (0.4)	156 (0.4)
Health conditions									
Type 1 diabetes	5279 (0.4)	662 (0.5)	446 (0.6)	5323 (0.4)	350 (1.1)	714 (1.4)	5490 (0.4)	273 (1.4)	624 (1.7)
Venous thromboembolism	7932 (0.7)	1090 (0.9)	596 (0.8)	8906 (0.7)	288 (0.9)	424 (0.8)	9133 (0.7)	180 (0.9)	305 (0.9)
Migraine	129226 (10.6)	16738 (13.1)	9284 (11.7)	144157 (10.8)	4632 (14.7)	6459 (12.9)	147580 (10.8)	2909 (15.1)	4759 (13.3)
Rheumatoid arthritis	4314 (0.4)	531 (0.4)	337 (0.4)	4732 (0.4)	153 (0.5)	297 (0.6)	4821 (0.4)	118 (0.6)	243 (0.7)
SLE	1865 (0.2)	299 (0.2)	176 (0.2)	2043 (0.2)	86 (0.3)	211 (0.4)	2095 (0.2)	64 (0.3)	181 (0.5)
Severe mental illness	9428 (0.8)	1157 (0.9)	822 (1.0)	10648 (0.8)	300 (1.0)	459 (0.9)	10866 (0.8)	189 (1.0)	352 (1.0)
Atrial fibrillation	1042 (0.1)	157 (0.1)	114 (0.1)	1166 (0.1)	51 (0.2)	96 (0.2)	1205 (0.1)	28 (0.1)	80 (0.2)
Learning disability	4206 (0.3)	211 (0.2)	161 (0.2)	4357 (0.3)	65 (0.2)	156 (0.3)	4406 (0.3)	42 (0.2)	130 (0.4)
Hypertension	31600 (2.6)	4007 (3.1)	2947 (3.7)	18929 (1.4)	4284 (13.6)	15341 (30.7)	22069 (1.6)	3068 (15.9)	13417 (37.5)
T2DM	12783 (1.1)	1643 (1.3)	1264 (1.6)	12845 (1.0)	758 (2.4)	2087 (4.2)	13267 (1.0)	558 (2.9)	1865 (5.2)
CKD	1386 (0.1)	148 (0.1)	141 (0.2)	1095 (0.1)	109 (0.3)	471 (0.9)	1135 (0.1)	98 (0.5)	442 (1.2)
CVD outcomes and follow-up									
Outcomes	13550 (1.1)	1334 (1.0)	1185 (1.5)	14327 (1.1)	478 (1.5)	1264 (2.5)	14721 (1.1)	318 (1.7)	1030 (2.9)
Median (IQR) Follow-up time, years	4.22 [1.48, 9.78]	4.45 [1.62, 9.23]	4.05 [1.40, 9.37]	4.22 [1.48, 9.69]	4.95 [1.85, 9.91]	4.28 [1.55, 9.62]	4.23 [1.49, 9.70]	4.77 [1.76, 9.54]	4.22 [1.53, 9.37]

Baseline demographic, socioeconomic, lifestyle, clinical, reproductive, medication, and comorbidity characteristics of women included in the CVD outcome cohort, stratified by history of pregnancy complications. Exposure categories are presented as *no history*, *previous history*, or *latest pregnancy* affected for each complication. Continuous variables are reported as mean (standard deviation) or median (interquartile range), and categorical variables as number (percentage). Outcomes represent incident CVD during follow-up. **Abbreviations:** BMI, body mass index; CKD, chronic kidney disease; CVD, cardiovascular disease; GDM, gestational diabetes mellitus; IMD, Index of Multiple Deprivation; IQR, interquartile range; SBP, systolic blood pressure; SGA, small for gestational age; SLE, systemic lupus erythematosus; T2DM, type 2 diabetes mellitus.

Table 4 (continued): Baseline Characteristics by exposure status in the CVD cohort

	GDM			Placental abruption			Stillbirth		
	**No history**	**Previous**	**Latest**	**No history**	**Previous**	**Latest**	**No history**	**Previous**	**Latest**
n	1349280	18477	34431	1412520	5687	4080	1407269	11762	3256
Mean (sd) age, years	33.06 (6.62)	35.94 (5.39)	34.50 (5.25)	33.20 (6.62)	34.58 (5.76)	31.65 (6.20)	33.18 (6.62)	35.25 (5.87)	31.99 (6.84)
Index of Multiple Deprivation (IMD)									
1	292827 (21.7)	4777 (25.9)	8618 (25.0)	309674 (21.9)	1701 (29.9)	1142 (28.0)	308036 (21.9)	3448 (29.3)	1033 (31.7)
2	286140 (21.2)	4319 (23.4)	8051 (23.4)	301595 (21.4)	1213 (21.3)	884 (21.7)	300103 (21.3)	2748 (23.4)	841 (25.8)
3	254973 (18.9)	3303 (17.9)	6350 (18.4)	266310 (18.9)	961 (16.9)	727 (17.8)	265466 (18.9)	1988 (16.9)	544 (16.7)
4	254316 (18.8)	3085 (16.7)	5792 (16.8)	264434 (18.7)	941 (16.5)	671 (16.4)	263753 (18.7)	1862 (15.8)	431 (13.2)
5	258816 (19.2)	2966 (16.1)	5580 (16.2)	268227 (19.0)	864 (15.2)	649 (15.9)	267637 (19.0)	1701 (14.5)	402 (12.3)
Missing IMD	2208 (0.2)	27 (0.1)	40 (0.1)	2280 (0.2)	7 (0.1)	7 (0.2)	2274 (0.2)	15 (0.1)	5 (0.2)
Median (IQR) BMI	24.56 [21.78, 28.70]	28.67 [24.39, 34]	28.87 [24.50, 34]	24.75 [21.88, 29]	25.16 [22, 29.60]	24.46 [21.50, 28.65]	24.70 [21.84, 29]	26.40 [22.80, 31.20]	25.70 [22.27, 30.30]
Missing BMI	200662 (14.9)	1175 (6.4)	2678 (7.8)	203755 (14.4)	770 (13.5)	631 (15.5)	203219 (14.4)	1401 (11.9)	536 (16.5)
Median (IQR) SBP	116 [108, 124]	119 [110, 128]	118 [110, 126]	116 [109, 124]	116 [109, 125]	115 [108, 123]	116 [109, 124]	118 [110, 126]	117 [110, 125]
Missing SBP	40707 (3.0)	97 (0.5)	391 (1.1)	41084 (2.9)	89 (1.6)	118 (2.9)	40912 (2.9)	232 (2.0)	147 (4.5)
Ethnicity									
White	840611 (62.3)	10240 (55.4)	18961 (55.1)	875037 (61.9)	3563 (62.7)	2377 (58.3)	872709 (62.0)	6692 (56.9)	1576 (48.4)
Black	62847 (4.7)	1187 (6.4)	2249 (6.5)	67680 (4.8)	328 (5.8)	216 (5.3)	66875 (4.8)	1024 (8.7)	325 (10.0)
South Asian	104599 (7.8)	3975 (21.5)	7443 (21.6)	119233 (8.4)	551 (9.7)	337 (8.3)	118244 (8.4)	1509 (12.8)	368 (11.3)
Mixed ethnicity	20249 (1.5)	332 (1.8)	666 (1.9)	21482 (1.5)	95 (1.7)	66 (1.6)	21369 (1.5)	221 (1.9)	53 (1.6)

Other ethnicity	15374 (1.1)	339 (1.8)	668 (1.9)	16520 (1.2)	67 (1.2)	40 (1.0)	16411 (1.2)	183 (1.6)	33 (1.0)
Missing ethnicity	305600 (22.6)	2404 (13.0)	4444 (12.9)	312568 (22.1)	1083 (19.0)	1044 (25.6)	311661 (22.1)	2133 (18.1)	901 (27.7)
Smoking status									
Non-smoker	718391 (53.3)	11973 (64.9)	21999 (63.9)	759653 (53.8)	2788 (49.0)	1953 (47.9)	756484 (53.8)	6269 (53.3)	1641 (50.4)
Current smoker	313654 (23.3)	3105 (16.8)	5677 (16.5)	323967 (22.9)	1745 (30.7)	1219 (29.9)	323024 (23.0)	2971 (25.3)	936 (28.8)
Ex-smoker	219600 (16.3)	3075 (16.7)	5881 (17.1)	230064 (16.3)	902 (15.9)	628 (15.4)	229353 (16.3)	1871 (15.9)	370 (11.4)
Missing smoking status	97028 (7.2)	307 (1.7)	863 (2.5)	98201 (7.0)	249 (4.4)	279 (6.8)	97777 (7.0)	644 (5.5)	308 (9.5)
Alcohol misuse	24098 (1.8)	374 (2.0)	640 (1.9)	25650 (1.8)	140 (2.5)	62 (1.5)	25565 (1.8)	227 (1.9)	60 (1.8)
Family history of CVD	93496 (6.9)	1681 (9.1)	2966 (8.6)	99693 (7.1)	423 (7.4)	282 (6.9)	99226 (7.1)	950 (8.1)	222 (6.8)
Median (IQR) Gravidity	3 [2, 4]	4 [3, 6]	3 [2, 4]	3 [2, 4]	4 [3, 6]	3 [2, 4]	3 [2, 4]	5 [4, 7]	3 [2, 5]
Gravidity									
1	304737 (22.6)	0 (0.0)	6776 (19.7)	314350 (22.3)	103 (1.8)	916 (22.5)	314596 (22.4)	90 (0.8)	683 (21.0)
2	323510 (24.0)	2748 (14.9)	8572 (24.9)	336688 (23.8)	836 (14.7)	970 (23.8)	336888 (23.9)	936 (8.0)	670 (20.6)
3	260518 (19.3)	3824 (20.7)	7054 (20.5)	273100 (19.3)	1041 (18.3)	746 (18.3)	272506 (19.4)	1794 (15.3)	587 (18.0)
4	179090 (13.3)	3646 (19.7)	4598 (13.4)	188587 (13.4)	956 (16.8)	513 (12.6)	187549 (13.3)	2106 (17.9)	401 (12.3)
5+	281425 (20.9)	8259 (44.7)	7431 (21.6)	299795 (21.2)	2751 (48.4)	935 (22.9)	295730 (21.0)	6836 (58.1)	915 (28.1)
Medications									
Statins	1167 (0.1)	24 (0.1)	43 (0.1)	3334 (0.2)	23 (0.4)	1 (0.0)	3281 (0.2)	69 (0.6)	8 (0.2)
Treated hypertension	8261 (0.6)	243 (1.3)	379 (1.1)	10833 (0.8)	65 (1.1)	31 (0.8)	10718 (0.8)	175 (1.5)	36 (1.1)
Corticosteroids	3889 (0.3)	66 (0.4)	147 (0.4)	4280 (0.3)	31 (0.5)	12 (0.3)	4251 (0.3)	54 (0.5)	18 (0.6)
Antipsycotics	4383 (0.3)	97 (0.5)	158 (0.5)	4917 (0.3)	24 (0.4)	14 (0.3)	4892 (0.3)	50 (0.4)	13 (0.4)
Health conditions									
Type 1 diabetes				6318 (0.4)	39 (0.7)	30 (0.7)	6178 (0.4)	165 (1.4)	44 (1.4)
Venous thromboembolism	8880 (0.7)	154 (0.8)	288 (0.8)	9496 (0.7)	79 (1.4)	43 (1.1)	9410 (0.7)	173 (1.5)	35 (1.1)
Migraine	145804 (10.8)	2593 (14.0)	4281 (12.4)	154027 (10.9)	773 (13.6)	448 (11.0)	153553 (10.9)	1355 (11.5)	340 (10.4)
Rheumatoid arthritis	4659 (0.3)	80 (0.4)	195 (0.6)	5144 (0.4)	24 (0.4)	14 (0.3)	5103 (0.4)	70 (0.6)	9 (0.3)
SLE	2195 (0.2)	24 (0.1)	53 (0.2)	2312 (0.2)	16 (0.3)	12 (0.3)	2277 (0.2)	52 (0.4)	11 (0.3)
Severe mental illness	10361 (0.8)	213 (1.2)	361 (1.0)	11302 (0.8)	77 (1.4)	28 (0.7)	11247 (0.8)	124 (1.1)	36 (1.1)
Atrial fibrillation	1190 (0.1)	18 (0.1)	37 (0.1)	1303 (0.1)	6 (0.1)	4 (0.1)	1295 (0.1)	15 (0.1)	3 (0.1)

Learning disability	4221 (0.3)	53 (0.3)	82 (0.2)	4542 (0.3)	26 (0.5)	10 (0.2)	4516 (0.3)	48 (0.4)	14 (0.4)
Hypertension	31499 (2.3)	991 (5.4)	1585 (4.6)	38099 (2.7)	308 (5.4)	147 (3.6)	37697 (2.7)	728 (6.2)	129 (4.0)
T2DM				15564 (1.1)	90 (1.6)	36 (0.9)	15210 (1.1)	401 (3.4)	79 (2.4)
CKD	1333 (0.1)	17 (0.1)	33 (0.1)	1663 (0.1)	10 (0.2)	2 (0.0)	1641 (0.1)	24 (0.2)	10 (0.3)
CVD outcomes and follow-up									
Outcomes	14416 (1.1)	181 (1.0)	316 (0.9)	15939 (1.1)	93 (1.6)	37 (0.9)	15790 (1.1)	246 (2.1)	33 (1.0)
Median (IQR) Follow-up time, years	4.32 [1.51, 9.88]	2.86 [1.17, 6.17]	2.78 [1.09, 5.96]	4.24 [1.49, 9.70]	4.41 [1.62, 9.45]	3.15 [1.16, 8.07]	4.24 [1.49, 9.69]	4.57 [1.61, 9.87]	2.63 [0.87, 7.41]

Baseline demographic, socioeconomic, lifestyle, clinical, reproductive, medication, and comorbidity characteristics of women included in the CVD outcome cohort, stratified by history of pregnancy complications. Exposure categories are presented as *no history*, *previous history*, or *latest pregnancy* affected for each complication. Continuous variables are reported as mean (standard deviation) or median (interquartile range), and categorical variables as number (percentage). Outcomes represent incident CVD during follow-up. **Abbreviations:** BMI, body mass index; CKD, chronic kidney disease; CVD, cardiovascular disease; GDM, gestational diabetes mellitus; IMD, Index of Multiple Deprivation; IQR, interquartile range; SBP, systolic blood pressure; SGA, small for gestational age; SLE, systemic lupus erythematosus; T2DM, type 2 diabetes mellitus.

Table 4 (continued): Baseline Characteristics by exposure status in the CVD cohort

	**Preterm birth**			**SGA**			**Postnatal depression**		
	**No history**	**Previous**	**Latest**	**No history**	**Previous**	**Latest**	**No history**	**Previous**	**Latest**
n	1373643	25383	23261	1365831	21968	34488	1188078	46018	188191
Mean (sd) age, years	33.24 (6.63)	32.67 (6.29)	31.43 (6.23)	33.24 (6.63)	33.69 (5.70)	31.18 (6.27)	33.15 (6.55)	33.56 (6.17)	33.39 (7.12)
Index of Multiple Deprivation (IMD)									
1	298652 (21.8)	7211 (28.4)	6654 (28.6)	294833 (21.6)	7157 (32.6)	10527 (30.5)	247406 (20.8)	13394 (29.1)	51717 (27.5)
2	292794 (21.3)	5644 (22.2)	5254 (22.6)	290407 (21.3)	5075 (23.1)	8210 (23.8)	252590 (21.3)	10244 (22.3)	40858 (21.7)
3	259241 (18.9)	4475 (17.6)	4282 (18.4)	258328 (18.9)	3591 (16.3)	6079 (17.6)	225855 (19.0)	8185 (17.8)	33958 (18.0)
4	258238 (18.8)	4114 (16.2)	3694 (15.9)	257937 (18.9)	3155 (14.4)	4954 (14.4)	226510 (19.1)	7446 (16.2)	32090 (17.1)
5	262499 (19.1)	3911 (15.4)	3330 (14.3)	262120 (19.2)	2964 (13.5)	4656 (13.5)	233824 (19.7)	6681 (14.5)	29235 (15.5)
Missing IMD	2219 (0.2)	28 (0.1)	47 (0.2)	2206 (0.2)	26 (0.1)	62 (0.2)	1893 (0.2)	68 (0.1)	333 (0.2)
Median (IQR) BMI	24.71 [21.89, 29]	25.10 [21.90, 29.70]	24.70 [21.60, 29.10]	24.80 [21.90, 29.03]	24.60 [21.60, 29]	23.70 [20.99, 27.70]	24.60 [21.80, 28.70]	25.80 [22.40, 30.65]	25.70 [22.20, 30.90]

Missing BMI	200161 (14.6)	2396 (9.4)	2599 (11.2)	197413 (14.5)	2287 (10.4)	5456 (15.8)	178017 (15.0)	4467 (9.7)	22672 (12.0)
Median (IQR) SBP	116 [109, 124]	117 [109, 124]	115 [108, 123]	116 [109, 124]	116 [108, 124]	115 [107, 123]	116 [108, 124]	117 [110, 124]	118 [110, 125]
Missing SBP	40501 (2.9)	369 (1.5)	421 (1.8)	39983 (2.9)	197 (0.9)	1111 (3.2)	37705 (3.2)	273 (0.6)	3313 (1.8)
Ethnicity									
White	849933 (61.9)	16687 (65.7)	14357 (61.7)	851044 (62.3)	12294 (56.0)	17639 (51.1)	715108 (60.2)	31487 (68.4)	134382 (71.4)
Black	65583 (4.8)	1379 (5.4)	1262 (5.4)	65137 (4.8)	1101 (5.0)	1986 (5.8)	62546 (5.3)	1471 (3.2)	4207 (2.2)
South Asian	115381 (8.4)	2502 (9.9)	2238 (9.6)	111062 (8.1)	3779 (17.2)	5280 (15.3)	110388 (9.3)	2566 (5.6)	7167 (3.8)
Mixed ethnicity	20786 (1.5)	443 (1.7)	414 (1.8)	20755 (1.5)	321 (1.5)	567 (1.6)	18622 (1.6)	592 (1.3)	2429 (1.3)
Other ethnicity	16032 (1.2)	301 (1.2)	294 (1.3)	15983 (1.2)	254 (1.2)	390 (1.1)	15073 (1.3)	321 (0.7)	1233 (0.7)
Missing ethnicity	305928 (22.3)	4071 (16.0)	4696 (20.2)	301850 (22.1)	4219 (19.2)	8626 (25.0)	266341 (22.4)	9581 (20.8)	38773 (20.6)
Smoking status									
Non-smoker	738438 (53.8)	13632 (53.7)	12324 (53.0)	736596 (54.0)	11259 (51.3)	16539 (48.0)	669656 (56.4)	19523 (42.4)	75215 (40.0)
Current smoker	313535 (22.8)	6928 (27.3)	6468 (27.8)	308706 (22.6)	6989 (31.8)	11236 (32.6)	242135 (20.4)	16167 (35.2)	68629 (36.5)
Ex-smoker	223914 (16.3)	4037 (15.9)	3643 (15.7)	224346 (16.4)	3057 (13.9)	4191 (12.2)	186741 (15.7)	9319 (20.3)	35534 (18.9)
Missing smoking status	97148 (7.1)	773 (3.0)	808 (3.5)	95574 (7.0)	652 (3.0)	2503 (7.3)	89019 (7.5)	982 (2.1)	8728 (4.6)
Alcohol misuse	24642 (1.8)	656 (2.6)	554 (2.4)	24592 (1.8)	526 (2.4)	734 (2.1)	15869 (1.3)	1351 (2.9)	8632 (4.6)
Family history of CVD	96768 (7.0)	1939 (7.6)	1691 (7.3)	96433 (7.1)	1657 (7.5)	2308 (6.7)	80615 (6.8)	3704 (8.0)	16079 (8.5)
Median (IQR) Gravidity	3 [2, 4]	4 [2, 6]	3 [2, 4]	3 [2, 4]	4 [3, 6]	2 [1, 4]	3 [2, 4]	5 [3, 7]	3 [2, 5]
Gravidity									
1	307773 (22.4)	1997 (7.9)	5599 (24.1)	305168 (22.3)	0 (0.0)	10201 (29.6)	279748 (23.5)	0 (0.0)	35621 (18.9)
2	328355 (23.9)	4823 (19.0)	5316 (22.9)	326023 (23.9)	4267 (19.4)	8204 (23.8)	296848 (25.0)	5572 (12.1)	36074 (19.2)
3	265593 (19.3)	4959 (19.5)	4335 (18.6)	264187 (19.3)	4574 (20.8)	6126 (17.8)	233128 (19.6)	7910 (17.2)	33849 (18.0)
4	183229 (13.3)	3973 (15.7)	2854 (12.3)	182155 (13.3)	4057 (18.5)	3844 (11.1)	154674 (13.0)	8153 (17.7)	27229 (14.5)
5+	288693 (21.0)	9631 (37.9)	5157 (22.2)	288298 (21.1)	9070 (41.3)	6113 (17.7)	223680 (18.8)	24383 (53.0)	55418 (29.4)
Medications									
Statins	3264 (0.2)	75 (0.3)	19 (0.1)	3284 (0.2)	43 (0.2)	31 (0.1)	2037 (0.2)	133 (0.3)	1188 (0.6)
Treated hypertension	10564 (0.8)	241 (0.9)	124 (0.5)	10452 (0.8)	211 (1.0)	266 (0.8)	7489 (0.6)	433 (0.9)	3007 (1.6)
Corticosteroids	4122 (0.3)	99 (0.4)	102 (0.4)	4076 (0.3)	116 (0.5)	131 (0.4)	2915 (0.2)	191 (0.4)	1217 (0.6)
Antipsycotics	4691 (0.3)	165 (0.7)	99 (0.4)	4762 (0.3)	99 (0.5)	94 (0.3)	1083 (0.1)	186 (0.4)	3686 (2.0)

Health conditions									
Type 1 diabetes	5917 (0.4)	205 (0.8)	265 (1.1)	6285 (0.5)	51 (0.2)	51 (0.1)	4706 (0.4)	289 (0.6)	1392 (0.7)
Venous thromboembolism	9160 (0.7)	278 (1.1)	180 (0.8)	9157 (0.7)	228 (1.0)	233 (0.7)	7122 (0.6)	497 (1.1)	1999 (1.1)
Migraine	148874 (10.8)	3562 (14.0)	2812 (12.1)	148942 (10.9)	2872 (13.1)	3434 (10.0)	113938 (9.6)	8392 (18.2)	32918 (17.5)
Rheumatoid arthritis	4953 (0.4)	128 (0.5)	101 (0.4)	4931 (0.4)	117 (0.5)	134 (0.4)	3888 (0.3)	222 (0.5)	1072 (0.6)
SLE	2217 (0.2)	59 (0.2)	64 (0.3)	2204 (0.2)	68 (0.3)	68 (0.2)	1788 (0.2)	116 (0.3)	436 (0.2)
Severe mental illness	10907 (0.8)	309 (1.2)	191 (0.8)	10889 (0.8)	232 (1.1)	286 (0.8)	4683 (0.4)	923 (2.0)	5801 (3.1)
Atrial fibrillation	1273 (0.1)	25 (0.1)	15 (0.1)	1259 (0.1)	23 (0.1)	31 (0.1)	929 (0.1)	64 (0.1)	320 (0.2)
Learning disability	4292 (0.3)	200 (0.8)	86 (0.4)	4423 (0.3)	45 (0.2)	110 (0.3)	2840 (0.2)	226 (0.5)	1512 (0.8)
Hypertension	36941 (2.7)	944 (3.7)	669 (2.9)	36702 (2.7)	867 (3.9)	985 (2.9)	29235 (2.5)	1585 (3.4)	7734 (4.1)
T2DM	14840 (1.1)	507 (2.0)	343 (1.5)	15218 (1.1)	248 (1.1)	224 (0.6)	11222 (0.9)	760 (1.7)	3708 (2.0)
CKD	1548 (0.1)	73 (0.3)	54 (0.2)	1595 (0.1)	42 (0.2)	38 (0.1)	1225 (0.1)	70 (0.2)	380 (0.2)
CVD outcomes and follow-up									
Outcomes	15628 (1.1)	243 (1.0)	198 (0.9)	15475 (1.1)	239 (1.1)	355 (1.0)	11912 (1.0)	691 (1.5)	3466 (1.8)
Median (IQR) Follow-up time, years	4.31 [1.51, 9.85]	2.74 [1.09, 5.91]	2.90 [1.15, 5.93]	4.25 [1.49, 9.74]	4.48 [1.70, 8.91]	3.59 [1.33, 8.55]	4.33 [1.52, 9.94]	4.22 [1.56, 8.72]	3.69 [1.33, 8.49]

Baseline demographic, socioeconomic, lifestyle, clinical, reproductive, medication, and comorbidity characteristics of women included in the CVD outcome cohort, stratified by history of pregnancy complications. Exposure categories are presented as *no history*, *previous history*, or *latest pregnancy* affected for each complication. Continuous variables are reported as mean (standard deviation) or median (interquartile range), and categorical variables as number (percentage). Outcomes represent incident CVD during follow-up. **Abbreviations:** BMI, body mass index; CKD, chronic kidney disease; CVD, cardiovascular disease; GDM, gestational diabetes mellitus; IMD, Index of Multiple Deprivation; IQR, interquartile range; SBP, systolic blood pressure; SGA, small for gestational age; SLE, systemic lupus erythematosus; T2DM, type 2 diabetes mellitus.

Chronic Kidney Disease (CKD) outcome cohort

Table 5: Baseline Characteristics by exposure status in the CKD cohort

	Miscarriage			Gestational hypertension			Pre-eclampsia		
	**No history**	**Previous**	**Latest**	**No history**	**Previous**	**Latest**	**No history**	**Previous**	**Latest**

n	1222489	128792	79684	1348849	31735	50381	1375497	19408	36060
Mean (sd) age, years	32.84 (6.60)	35.03 (5.73)	35.99 (7.14)	33.14 (6.65)	34.30 (5.75)	34.60 (6.39)	33.14 (6.63)	34.54 (5.78)	35.29 (6.38)
Index of Multiple Deprivation (IMD)									
1	269114 (22.0)	27633 (21.5)	18187 (22.9)	295848 (21.9)	7117 (22.4)	11969 (23.8)	301772 (21.9)	4370 (22.5)	8792 (24.4)
2	263075 (21.5)	25645 (19.9)	17399 (21.8)	287971 (21.3)	6578 (20.7)	11570 (23.0)	293547 (21.3)	4087 (21.1)	8485 (23.5)
3	230952 (18.9)	23677 (18.4)	14934 (18.7)	254236 (18.8)	5911 (18.6)	9416 (18.7)	259340 (18.9)	3592 (18.5)	6631 (18.4)
4	227631 (18.6)	25083 (19.5)	14604 (18.3)	252442 (18.7)	5961 (18.8)	8915 (17.7)	257570 (18.7)	3572 (18.4)	6176 (17.1)
5	229795 (18.8)	26595 (20.6)	14338 (18.0)	256158 (19.0)	6127 (19.3)	8443 (16.8)	261037 (19.0)	3763 (19.4)	5928 (16.4)
Missing IMD	1922 (0.2)	159 (0.1)	222 (0.3)	2194 (0.2)	41 (0.1)	68 (0.1)	2231 (0.2)	24 (0.1)	48 (0.1)
Median (IQR) BMI	24.70 [21.80, 29]	25.10 [22.10, 29.49]	24.90 [21.90, 29.45]	24.60 [21.80, 28.76]	27.50 [23.60, 32.76]	28.04 [24, 33.20]	24.60 [21.80, 28.87]	27.50 [23.60, 32.79]	28.40 [24.20, 33.60]
Missing BMI	183031 (15.0)	13149 (10.2)	11181 (14.0)	199140 (14.8)	2926 (9.2)	5295 (10.5)	202251 (14.7)	1668 (8.6)	3442 (9.5)
Median (IQR) SBP	116 [109, 124]	116 [109, 124]	118 [110, 127]	115 [108, 123]	122 [114, 130]	126 [118, 135]	116 [108, 123]	122 [114, 132]	127 [119, 136]
Missing SBP	37381 (3.1)	1100 (0.9)	2900 (3.6)	40693 (3.0)	177 (0.6)	511 (1.0)	41005 (3.0)	96 (0.5)	280 (0.8)
Ethnicity									
White	752854 (61.6)	85303 (66.2)	48055 (60.3)	834952 (61.9)	20990 (66.1)	30270 (60.1)	852555 (62.0)	12674 (65.3)	20983 (58.2)
Black	57619 (4.7)	5681 (4.4)	5346 (6.7)	62012 (4.6)	1542 (4.9)	5092 (10.1)	63032 (4.6)	1156 (6.0)	4458 (12.4)
South Asian	103052 (8.4)	10671 (8.3)	6983 (8.8)	113978 (8.5)	2263 (7.1)	4465 (8.9)	115678 (8.4)	1527 (7.9)	3501 (9.7)
Mixed ethnicity	18485 (1.5)	1770 (1.4)	1489 (1.9)	20515 (1.5)	344 (1.1)	885 (1.8)	20789 (1.5)	244 (1.3)	711 (2.0)
Other ethnicity	14343 (1.2)	1351 (1.0)	1015 (1.3)	15965 (1.2)	249 (0.8)	495 (1.0)	16175 (1.2)	169 (0.9)	365 (1.0)
Missing ethnicity	276136 (22.6)	24016 (18.6)	16796 (21.1)	301427 (22.3)	6347 (20.0)	9174 (18.2)	307268 (22.3)	3638 (18.7)	6042 (16.8)
Smoking status									
Non-smoker	654323 (53.5)	73251 (56.9)	40837 (51.3)	719359 (53.4)	18957 (59.8)	30095 (59.8)	734325 (53.4)	11906 (61.4)	22180 (61.5)
Current smoker	281370 (23.0)	27303 (21.2)	21049 (26.5)	315257 (23.4)	5819 (18.3)	8646 (17.2)	320349 (23.3)	3389 (17.5)	5984 (16.6)
Ex-smoker	196577 (16.1)	24010 (18.7)	12593 (15.8)	218576 (16.2)	5773 (18.2)	8831 (17.5)	223641 (16.3)	3419 (17.6)	6120 (17.0)
Missing smoking status	89775 (7.3)	4170 (3.2)	5068 (6.4)	95057 (7.1)	1172 (3.7)	2784 (5.5)	96567 (7.0)	688 (3.5)	1758 (4.9)
Alcohol misuse	21998 (1.8)	2333 (1.8)	1853 (2.3)	24742 (1.8)	528 (1.7)	914 (1.8)	25170 (1.8)	307 (1.6)	707 (2.0)
Family history of CVD	85027 (7.0)	10011 (7.8)	6476 (8.1)	93841 (7.0)	2875 (9.1)	4798 (9.5)	96138 (7.0)	1809 (9.3)	3567 (9.9)
Median (IQR) Gravidity	2 [2, 4]	4 [3, 6]	3 [2, 5]	3 [2, 4]	4 [2, 5]	2 [1, 4]	3 [2, 4]	4 [3, 5]	2 [1, 4]

Gravidity									
1	300000 (24.5)	0 (0.0)	16977 (21.3)	301712 (22.4)	0 (0.0)	15265 (30.3)	306695 (22.3)	0 (0.0)	10282 (28.5)
2	312218 (25.5)	13791 (10.7)	14348 (18.0)	320590 (23.8)	7963 (25.1)	11804 (23.4)	327286 (23.8)	4767 (24.6)	8304 (23.0)
3	234279 (19.2)	28282 (22.0)	13977 (17.5)	260369 (19.3)	7420 (23.4)	8749 (17.4)	265765 (19.3)	4497 (23.2)	6276 (17.4)
4	153663 (12.6)	26295 (20.4)	11238 (14.1)	179755 (13.3)	5848 (18.4)	5593 (11.1)	183454 (13.3)	3582 (18.5)	4160 (11.5)
5+	222329 (18.2)	60424 (46.9)	23144 (29.0)	286423 (21.2)	10504 (33.1)	8970 (17.8)	292297 (21.3)	6562 (33.8)	7038 (19.5)
Medications									
Statins	3389 (0.3)	276 (0.2)	240 (0.3)	3094 (0.2)	174 (0.5)	637 (1.3)	3197 (0.2)	127 (0.7)	581 (1.6)
Treated hypertension	9593 (0.8)	985 (0.8)	809 (1.0)	5765 (0.4)	811 (2.6)	4811 (9.5)	6179 (0.4)	663 (3.4)	4545 (12.6)
Corticosteroids	3564 (0.3)	475 (0.4)	330 (0.4)	3995 (0.3)	121 (0.4)	253 (0.5)	4085 (0.3)	75 (0.4)	209 (0.6)
Antipsycotics	4235 (0.3)	451 (0.4)	373 (0.5)	4718 (0.3)	132 (0.4)	209 (0.4)	4815 (0.4)	80 (0.4)	164 (0.5)
Health conditions									
Type 1 diabetes	5297 (0.4)	652 (0.5)	433 (0.5)	5364 (0.4)	352 (1.1)	666 (1.3)	5533 (0.4)	274 (1.4)	575 (1.6)
Venous thromboembolism	8184 (0.7)	1131 (0.9)	620 (0.8)	9182 (0.7)	308 (1.0)	445 (0.9)	9421 (0.7)	195 (1.0)	319 (0.9)
Migraine	130642 (10.7)	16950 (13.2)	9410 (11.8)	145712 (10.8)	4699 (14.8)	6591 (13.1)	149200 (10.8)	2946 (15.2)	4856 (13.5)
Rheumatoid arthritis	4379 (0.4)	546 (0.4)	340 (0.4)	4812 (0.4)	154 (0.5)	299 (0.6)	4902 (0.4)	120 (0.6)	243 (0.7)
SLE	1894 (0.2)	301 (0.2)	178 (0.2)	2092 (0.2)	87 (0.3)	194 (0.4)	2145 (0.2)	65 (0.3)	163 (0.5)
Severe mental illness	9559 (0.8)	1171 (0.9)	838 (1.1)	10787 (0.8)	305 (1.0)	476 (0.9)	11016 (0.8)	189 (1.0)	363 (1.0)
Atrial fibrillation	1194 (0.1)	170 (0.1)	128 (0.2)	1321 (0.1)	55 (0.2)	116 (0.2)	1367 (0.1)	30 (0.2)	95 (0.3)
Learning disability	4252 (0.3)	218 (0.2)	164 (0.2)	4418 (0.3)	66 (0.2)	150 (0.3)	4468 (0.3)	42 (0.2)	124 (0.3)
Hypertension	32396 (2.7)	4088 (3.2)	3005 (3.8)	19608 (1.5)	4350 (13.7)	15531 (30.8)	22839 (1.7)	3098 (16.0)	13552 (37.6)
CVD	8599 (0.7)	1024 (0.8)	730 (0.9)	9069 (0.7)	370 (1.2)	914 (1.8)	9348 (0.7)	250 (1.3)	755 (2.1)
T2DM	13168 (1.1)	1683 (1.3)	1294 (1.6)	13209 (1.0)	779 (2.5)	2157 (4.3)	13656 (1.0)	568 (2.9)	1921 (5.3)
CKD outcomes and follow-up									
Outcomes	4462 (0.4)	417 (0.3)	409 (0.5)	4430 (0.3)	201 (0.6)	657 (1.3)	4559 (0.3)	146 (0.8)	583 (1.6)
Median (IQR) Follow-up time, years	4.24 [1.49, 9.84]	4.46 [1.62, 9.27]	4.07 [1.41, 9.43]	4.24 [1.49, 9.74]	4.96 [1.85, 9.93]	4.31 [1.56, 9.69]	4.25 [1.49, 9.76]	4.78 [1.77, 9.59]	4.25 [1.54, 9.43]

Baseline demographic, socioeconomic, lifestyle, clinical, reproductive, medication, and comorbidity characteristics of women included in the CKD outcome cohort, stratified by history of pregnancy complications. Exposure categories are presented as *no history*, *previous history*, or *latest pregnancy* affected for each complication. Continuous

variables are reported as mean (standard deviation) or median (interquartile range), and categorical variables as number (percentage). Outcomes represent incident CKD during follow-up. **Abbreviations:** BMI, body mass index; CKD, chronic kidney disease; CVD, cardiovascular disease; GDM, gestational diabetes mellitus; IMD, Index of Multiple Deprivation; IQR, interquartile range; SBP, systolic blood pressure; SGA, small for gestational age; SLE, systemic lupus erythematosus; T2DM, type 2 diabetes mellitus.

Table 5 (continued): Baseline Characteristics by exposure status in the CKD cohort

	GDM			Placental abruption			Stillbirth		
	**No history**	**Previous**	**Latest**	**No history**	**Previous**	**Latest**	**No history**	**Previous**	**Latest**
n	1357128	18609	34678	1421121	5741	4103	1415805	11872	3288
Mean (sd) age, years	33.08 (6.63)	35.95 (5.40)	34.51 (5.25)	33.21 (6.63)	34.61 (5.78)	31.66 (6.20)	33.20 (6.63)	35.26 (5.87)	32.03 (6.87)
Index of Multiple Deprivation (IMD)									
1	294921 (21.7)	4820 (25.9)	8691 (25.1)	312060 (22.0)	1723 (30.0)	1151 (28.1)	310394 (21.9)	3490 (29.4)	1050 (31.9)
2	288325 (21.2)	4350 (23.4)	8130 (23.4)	304008 (21.4)	1225 (21.3)	886 (21.6)	302494 (21.4)	2779 (23.4)	846 (25.7)
3	256392 (18.9)	3325 (17.9)	6399 (18.5)	267858 (18.8)	970 (16.9)	735 (17.9)	267010 (18.9)	2004 (16.9)	549 (16.7)
4	255522 (18.8)	3102 (16.7)	5813 (16.8)	265698 (18.7)	946 (16.5)	674 (16.4)	265008 (18.7)	1874 (15.8)	436 (13.3)
5	259751 (19.1)	2986 (16.0)	5605 (16.2)	269208 (18.9)	870 (15.2)	650 (15.8)	268616 (19.0)	1710 (14.4)	402 (12.2)
Missing IMD	2217 (0.2)	26 (0.1)	40 (0.1)	2289 (0.2)	7 (0.1)	7 (0.2)	2283 (0.2)	15 (0.1)	5 (0.2)
Median (IQR) BMI	24.58 [21.79, 28.70]	28.68 [24.39, 33.98]	28.90 [24.50, 34]	24.77 [21.90, 29]	25.20 [22, 29.60]	24.46 [21.50, 28.66]	24.70 [21.87, 29]	26.40 [22.81, 31.20]	25.70 [22.20, 30.30]
Missing BMI	202778 (14.9)	1194 (6.4)	2731 (7.9)	205952 (14.5)	777 (13.5)	632 (15.4)	205404 (14.5)	1414 (11.9)	543 (16.5)
Median (IQR) SBP	116 [108, 124]	119 [110, 128]	118 [110, 126]	116 [109, 124]	117 [109, 125]	115 [108, 123]	116 [109, 124]	118 [110, 126]	117 [110, 125]
Missing SBP	40795 (3.0)	97 (0.5)	392 (1.1)	41174 (2.9)	89 (1.6)	118 (2.9)	41002 (2.9)	232 (2.0)	147 (4.5)
Ethnicity									
White	845408 (62.3)	10324 (55.5)	19079 (55.0)	880230 (61.9)	3598 (62.7)	2384 (58.1)	877864 (62.0)	6754 (56.9)	1594 (48.5)
Black	63201 (4.7)	1202 (6.5)	2273 (6.6)	68095 (4.8)	332 (5.8)	219 (5.3)	67285 (4.8)	1034 (8.7)	327 (9.9)
South Asian	105033 (7.7)	3992 (21.5)	7484 (21.6)	119812 (8.4)	555 (9.7)	339 (8.3)	118818 (8.4)	1519 (12.8)	369 (11.2)

Mixed ethnicity	20332 (1.5)	334 (1.8)	670 (1.9)	21581 (1.5)	94 (1.6)	69 (1.7)	21467 (1.5)	223 (1.9)	54 (1.6)
Other ethnicity	15438 (1.1)	341 (1.8)	676 (1.9)	16600 (1.2)	69 (1.2)	40 (1.0)	16488 (1.2)	187 (1.6)	34 (1.0)
Missing ethnicity	307716 (22.7)	2416 (13.0)	4496 (13.0)	314803 (22.2)	1093 (19.0)	1052 (25.6)	313883 (22.2)	2155 (18.2)	910 (27.7)
Smoking status									
Non-smoker	721998 (53.2)	12043 (64.8)	22146 (63.9)	763643 (53.8)	2807 (48.9)	1961 (47.8)	760438 (53.7)	6320 (53.3)	1653 (50.3)
Current smoker	316211 (23.3)	3133 (16.9)	5726 (16.5)	326723 (23.0)	1769 (30.8)	1230 (30.0)	325758 (23.0)	3012 (25.4)	952 (29.0)
Ex-smoker	221011 (16.3)	3109 (16.7)	5929 (17.1)	231637 (16.3)	911 (15.9)	632 (15.4)	230918 (16.3)	1889 (15.9)	373 (11.3)
Missing smoking status	97301 (7.2)	307 (1.7)	866 (2.5)	98483 (6.9)	251 (4.4)	279 (6.8)	98060 (6.9)	644 (5.4)	309 (9.4)
Alcohol misuse	24383 (1.8)	379 (2.0)	644 (1.9)	25976 (1.8)	145 (2.5)	63 (1.5)	25890 (1.8)	232 (2.0)	62 (1.9)
Family history of CVD	94428 (7.0)	1703 (9.2)	2993 (8.6)	100795 (7.1)	434 (7.6)	285 (6.9)	100320 (7.1)	967 (8.1)	227 (6.9)
Median (IQR) Gravidity	3 [2, 4]	4 [3, 6]	3 [2, 4]	3 [2, 4]	4 [3, 6]	3 [2, 4]	3 [2, 4]	5 [4, 7]	3 [2, 5]
Gravidity									
1	306243 (22.6)	0 (0.0)	6810 (19.6)	315954 (22.2)	104 (1.8)	919 (22.4)	316201 (22.3)	90 (0.8)	686 (20.9)
2	325218 (24.0)	2763 (14.8)	8641 (24.9)	338538 (23.8)	842 (14.7)	977 (23.8)	338736 (23.9)	938 (7.9)	683 (20.8)
3	262007 (19.3)	3854 (20.7)	7105 (20.5)	274736 (19.3)	1050 (18.3)	752 (18.3)	274137 (19.4)	1808 (15.2)	593 (18.0)
4	180111 (13.3)	3665 (19.7)	4631 (13.4)	189717 (13.3)	966 (16.8)	513 (12.5)	188666 (13.3)	2128 (17.9)	402 (12.2)
5+	283549 (20.9)	8327 (44.7)	7491 (21.6)	302176 (21.3)	2779 (48.4)	942 (23.0)	298065 (21.1)	6908 (58.2)	924 (28.1)
Medications									
Statins	1527 (0.1)	28 (0.2)	50 (0.1)	3879 (0.3)	25 (0.4)	1 (0.0)	3813 (0.3)	78 (0.7)	14 (0.4)
Treated hypertension	8567 (0.6)	244 (1.3)	390 (1.1)	11290 (0.8)	67 (1.2)	30 (0.7)	11161 (0.8)	186 (1.6)	40 (1.2)
Corticosteroids	3929 (0.3)	68 (0.4)	150 (0.4)	4322 (0.3)	34 (0.6)	13 (0.3)	4297 (0.3)	54 (0.5)	18 (0.5)
Antipsycotics	4460 (0.3)	99 (0.5)	162 (0.5)	5021 (0.4)	24 (0.4)	14 (0.3)	4994 (0.4)	51 (0.4)	14 (0.4)
Health conditions									
Type 1 diabetes				6311 (0.4)	39 (0.7)	32 (0.8)	6171 (0.4)	166 (1.4)	45 (1.4)
Venous thromboembolism	9145 (0.7)	159 (0.9)	300 (0.9)	9799 (0.7)	85 (1.5)	51 (1.2)	9707 (0.7)	181 (1.5)	47 (1.4)
Migraine	147339 (10.9)	2636 (14.2)	4330 (12.5)	155767 (11.0)	784 (13.7)	451 (11.0)	155278 (11.0)	1383 (11.6)	341 (10.4)
Rheumatoid arthritis	4728 (0.3)	81 (0.4)	194 (0.6)	5222 (0.4)	27 (0.5)	16 (0.4)	5184 (0.4)	69 (0.6)	12 (0.4)
SLE	2214 (0.2)	25 (0.1)	52 (0.1)	2337 (0.2)	18 (0.3)	18 (0.4)	2301 (0.2)	57 (0.5)	15 (0.5)
Severe mental illness	10484 (0.8)	216 (1.2)	364 (1.0)	11462 (0.8)	78 (1.4)	28 (0.7)	11403 (0.8)	125 (1.1)	40 (1.2)

Atrial fibrillation	1338 (0.1)	20 (0.1)	38 (0.1)	1471 (0.1)	9 (0.2)	12 (0.3)	1460 (0.1)	18 (0.2)	14 (0.4)
Learning disability	4276 (0.3)	54 (0.3)	82 (0.2)	4597 (0.3)	27 (0.5)	10 (0.2)	4571 (0.3)	48 (0.4)	15 (0.5)
Hypertension	32170 (2.4)	1007 (5.4)	1613 (4.7)	39016 (2.7)	318 (5.5)	155 (3.8)	38597 (2.7)	745 (6.3)	147 (4.5)
CVD	9181 (0.7)	149 (0.8)	280 (0.8)	10264 (0.7)	64 (1.1)	25 (0.6)	10177 (0.7)	134 (1.1)	42 (1.3)
T2DM				16007 (1.1)	93 (1.6)	45 (1.1)	15648 (1.1)	407 (3.4)	90 (2.7)
CKD outcomes and follow-up									
Outcomes	4553 (0.3)	46 (0.2)	78 (0.2)	5243 (0.4)	26 (0.5)	19 (0.5)	5190 (0.4)	75 (0.6)	23 (0.7)
Median (IQR) Follow-up time, years	4.34 [1.51, 9.93]	2.88 [1.18, 6.23]	2.79 [1.09, 5.99]	4.26 [1.49, 9.75]	4.42 [1.62, 9.52]	3.16 [1.16, 8.04]	4.26 [1.49, 9.75]	4.60 [1.61, 9.97]	2.63 [0.87, 7.44]

Baseline demographic, socioeconomic, lifestyle, clinical, reproductive, medication, and comorbidity characteristics of women included in the CKD outcome cohort, stratified by history of pregnancy complications. Exposure categories are presented as *no history*, *previous history*, or *latest pregnancy* affected for each complication. Continuous variables are reported as mean (standard deviation) or median (interquartile range), and categorical variables as number (percentage). Outcomes represent incident CKD during follow-up. **Abbreviations:** BMI, body mass index; CKD, chronic kidney disease; CVD, cardiovascular disease; GDM, gestational diabetes mellitus; IMD, Index of Multiple Deprivation; IQR, interquartile range; SBP, systolic blood pressure; SGA, small for gestational age; SLE, systemic lupus erythematosus; T2DM, type 2 diabetes mellitus.

Table 5 (continued): Baseline Characteristics by exposure status in the CKD cohort

	Preterm birth			SGA			Postnatal depression		
	**No history**	**Previous**	**Latest**	**No history**	**Previous**	**Latest**	**No history**	**Previous**	**Latest**

n	1381965	25609	23391	1374160	22129	34676	1194076	46444	190445
Mean (sd) age, years	33.25 (6.63)	32.67 (6.29)	31.43 (6.23)	33.26 (6.64)	33.71 (5.71)	31.19 (6.27)	33.16 (6.56)	33.58 (6.17)	33.43 (7.14)
Index of Multiple Deprivation (IMD)									
1	300950 (21.8)	7288 (28.5)	6696 (28.6)	297152 (21.6)	7196 (32.5)	10586 (30.5)	248931 (20.9)	13533 (29.2)	52470 (27.6)
2	295119 (21.4)	5706 (22.3)	5294 (22.6)	292735 (21.3)	5117 (23.1)	8267 (23.8)	254332 (21.3)	10352 (22.3)	41435 (21.8)
3	260739 (18.9)	4518 (17.6)	4306 (18.4)	259814 (18.9)	3627 (16.4)	6122 (17.7)	226956 (19.0)	8264 (17.8)	34343 (18.0)
4	259467 (18.8)	4139 (16.2)	3712 (15.9)	259157 (18.9)	3188 (14.4)	4973 (14.3)	227391 (19.0)	7517 (16.2)	32410 (17.0)
5	263463 (19.1)	3929 (15.3)	3336 (14.3)	263087 (19.1)	2975 (13.4)	4666 (13.5)	234569 (19.6)	6709 (14.4)	29450 (15.5)
Missing IMD	2227 (0.2)	29 (0.1)	47 (0.2)	2215 (0.2)	26 (0.1)	62 (0.2)	1897 (0.2)	69 (0.1)	337 (0.2)
Median (IQR) BMI	24.75 [21.90, 29]	25.10 [21.90, 29.70]	24.70 [21.60, 29.10]	24.80 [21.90, 29.06]	24.62 [21.60, 29]	23.72 [21, 27.70]	24.60 [21.80, 28.70]	25.80 [22.40, 30.69]	25.70 [22.21, 30.90]
Missing BMI	202305 (14.6)	2430 (9.5)	2626 (11.2)	199533 (14.5)	2317 (10.5)	5511 (15.9)	179746 (15.1)	4535 (9.8)	23080 (12.1)
Median (IQR) SBP	116 [109, 124]	117 [109, 124]	115 [108, 123]	116 [109, 124]	116 [108, 124]	115 [107, 123]	116 [108, 124]	117 [110, 125]	118 [110, 125]
Missing SBP	40586 (2.9)	371 (1.4)	424 (1.8)	40071 (2.9)	197 (0.9)	1113 (3.2)	37775 (3.2)	273 (0.6)	3333 (1.8)
Ethnicity									
White	854942 (61.9)	16827 (65.7)	14443 (61.7)	856101 (62.3)	12379 (55.9)	17732 (51.1)	718483 (60.2)	31763 (68.4)	135966 (71.4)
Black	65994 (4.8)	1387 (5.4)	1265 (5.4)	65537 (4.8)	1111 (5.0)	1998 (5.8)	62880 (5.3)	1489 (3.2)	4277 (2.2)
South Asian	115931 (8.4)	2521 (9.8)	2254 (9.6)	111617 (8.1)	3799 (17.2)	5290 (15.3)	110841 (9.3)	2594 (5.6)	7271 (3.8)
Mixed ethnicity	20881 (1.5)	447 (1.7)	416 (1.8)	20851 (1.5)	323 (1.5)	570 (1.6)	18696 (1.6)	601 (1.3)	2447 (1.3)
Other ethnicity	16112 (1.2)	303 (1.2)	294 (1.3)	16060 (1.2)	257 (1.2)	392 (1.1)	15139 (1.3)	323 (0.7)	1247 (0.7)
Missing ethnicity	308105 (22.3)	4124 (16.1)	4719 (20.2)	303994 (22.1)	4260 (19.3)	8694 (25.1)	268037 (22.4)	9674 (20.8)	39237 (20.6)
Smoking status									
Non-smoker	742279 (53.7)	13740 (53.7)	12392 (53.0)	740485 (53.9)	11317 (51.2)	16609 (47.9)	672664 (56.4)	19707 (42.5)	76040 (39.9)
Current smoker	316206 (22.9)	7014 (27.4)	6502 (27.8)	311331 (22.7)	7073 (32.0)	11318 (32.7)	243817 (20.4)	16326 (35.2)	69579 (36.6)
Ex-smoker	225443 (16.3)	4068 (15.9)	3669 (15.7)	225886 (16.4)	3075 (13.9)	4219 (12.2)	187825 (15.7)	9396 (20.2)	35959 (18.9)
Missing smoking status	97429 (7.1)	774 (3.0)	810 (3.5)	95849 (7.0)	653 (3.0)	2511 (7.2)	89243 (7.5)	988 (2.1)	8782 (4.6)
Alcohol misuse	24964 (1.8)	662 (2.6)	558 (2.4)	24912 (1.8)	531 (2.4)	741 (2.1)	15987 (1.3)	1380 (3.0)	8817 (4.6)
Family history of CVD	97841 (7.1)	1969 (7.7)	1704 (7.3)	97500 (7.1)	1685 (7.6)	2329 (6.7)	81312 (6.8)	3763 (8.1)	16439 (8.6)
Median (IQR) Gravidity	3 [2, 4]	4 [2, 6]	3 [2, 4]	3 [2, 4]	4 [3, 6]	2 [1, 4]	3 [2, 4]	5 [3, 7]	3 [2, 5]

Gravidity									
1	309317 (22.4)	2021 (7.9)	5639 (24.1)	306732 (22.3)	0 (0.0)	10245 (29.5)	281015 (23.5)	0 (0.0)	35962 (18.9)
2	330152 (23.9)	4861 (19.0)	5344 (22.8)	327832 (23.9)	4283 (19.4)	8242 (23.8)	298256 (25.0)	5626 (12.1)	36475 (19.2)
3	267181 (19.3)	5010 (19.6)	4347 (18.6)	265775 (19.3)	4599 (20.8)	6164 (17.8)	234306 (19.6)	7965 (17.1)	34267 (18.0)
4	184325 (13.3)	3997 (15.6)	2874 (12.3)	183251 (13.3)	4089 (18.5)	3856 (11.1)	155448 (13.0)	8212 (17.7)	27536 (14.5)
5+	290990 (21.1)	9720 (38.0)	5187 (22.2)	290570 (21.1)	9158 (41.4)	6169 (17.8)	225051 (18.8)	24641 (53.1)	56205 (29.5)
Medications									
Statins	3793 (0.3)	83 (0.3)	29 (0.1)	3819 (0.3)	55 (0.2)	31 (0.1)	2308 (0.2)	151 (0.3)	1446 (0.8)
Treated hypertension	11024 (0.8)	238 (0.9)	125 (0.5)	10903 (0.8)	215 (1.0)	269 (0.8)	7688 (0.6)	445 (1.0)	3254 (1.7)
Corticosteroids	4166 (0.3)	102 (0.4)	101 (0.4)	4125 (0.3)	114 (0.5)	130 (0.4)	2923 (0.2)	191 (0.4)	1255 (0.7)
Antipsycotics	4792 (0.3)	168 (0.7)	99 (0.4)	4864 (0.4)	99 (0.4)	96 (0.3)	1108 (0.1)	188 (0.4)	3763 (2.0)
Health conditions									
Type 1 diabetes	5921 (0.4)	201 (0.8)	260 (1.1)	6285 (0.5)	49 (0.2)	48 (0.1)	4688 (0.4)	292 (0.6)	1402 (0.7)
Venous thromboembolism	9460 (0.7)	289 (1.1)	186 (0.8)	9459 (0.7)	237 (1.1)	239 (0.7)	7296 (0.6)	516 (1.1)	2123 (1.1)
Migraine	150536 (10.9)	3622 (14.1)	2844 (12.2)	150623 (11.0)	2912 (13.2)	3467 (10.0)	114984 (9.6)	8505 (18.3)	33513 (17.6)
Rheumatoid arthritis	5035 (0.4)	131 (0.5)	99 (0.4)	5014 (0.4)	118 (0.5)	133 (0.4)	3926 (0.3)	226 (0.5)	1113 (0.6)
SLE	2244 (0.2)	67 (0.3)	62 (0.3)	2241 (0.2)	65 (0.3)	67 (0.2)	1799 (0.2)	114 (0.2)	460 (0.2)
Severe mental illness	11061 (0.8)	313 (1.2)	194 (0.8)	11041 (0.8)	237 (1.1)	290 (0.8)	4733 (0.4)	936 (2.0)	5899 (3.1)
Atrial fibrillation	1437 (0.1)	36 (0.1)	19 (0.1)	1432 (0.1)	24 (0.1)	36 (0.1)	1029 (0.1)	75 (0.2)	388 (0.2)
Learning disability	4346 (0.3)	202 (0.8)	86 (0.4)	4478 (0.3)	46 (0.2)	110 (0.3)	2875 (0.2)	229 (0.5)	1530 (0.8)
Hypertension	37870 (2.7)	955 (3.7)	664 (2.8)	37621 (2.7)	877 (4.0)	991 (2.9)	29675 (2.5)	1630 (3.5)	8184 (4.3)
CVD	9870 (0.7)	299 (1.2)	184 (0.8)	9924 (0.7)	203 (0.9)	226 (0.7)	7223 (0.6)	496 (1.1)	2634 (1.4)
T2DM	15271 (1.1)	523 (2.0)	351 (1.5)	15671 (1.1)	251 (1.1)	223 (0.6)	11459 (1.0)	782 (1.7)	3904 (2.0)
CKD outcomes and follow-up									
CKD outcome	5163 (0.4)	88 (0.3)	37 (0.2)	5123 (0.4)	61 (0.3)	104 (0.3)	4087 (0.3)	172 (0.4)	1029 (0.5)
Median (IQR) Follow-up time, years	4.33 [1.51, 9.91]	2.74 [1.09, 5.92]	2.91 [1.15, 5.96]	4.27 [1.50, 9.80]	4.50 [1.71, 8.93]	3.60 [1.33, 8.59]	4.35 [1.52, 9.98]	4.25 [1.57, 8.78]	3.72 [1.34, 8.56]

Baseline demographic, socioeconomic, lifestyle, clinical, reproductive, medication, and comorbidity characteristics of women included in the CKD outcome cohort, stratified by history of pregnancy complications. Exposure categories are presented as *no history*, *previous history*, or *latest pregnancy* affected for each complication. Continuous

BMJ Publishing Group Limited (BMJ) disclaims all liability and responsibility arising from any reliance placed on this supplemental material which has been supplied by the author(s)

variables are reported as mean (standard deviation) or median (interquartile range), and categorical variables as number (percentage). Outcomes represent incident CKD during follow-up. **Abbreviations:** BMI, body mass index; CKD, chronic kidney disease; CVD, cardiovascular disease; GDM, gestational diabetes mellitus; IMD, Index of Multiple Deprivation; IQR, interquartile range; SBP, systolic blood pressure; SGA, small for gestational age; SLE, systemic lupus erythematosus; T2DM, type 2 diabetes mellitus.

Incidence rates

Table 6: Incidence rates for hypertension, type 2 diabetes, cardiovascular disease and chronic kidney disease in women with latest, previous and without a history of pregnancy complications

	Miscarriage			Gestational hypertension			Pre-eclampsia			GDM			Placental abruption		
Variable	**No history**	**Previous**	**Latest**	**No history**	**Previous**	**Latest**	**No history**	**Previous**	**Latest**	**No history**	**Previous**	**Latest**	**No history**	**Previous**	**Latest**
	**Hypertension**														
n	1188362	124533	76565	1327774	27279	34407	1351129	16233	22098	1323427	17578	32975	1380102	5416	3942
Events	41455	4084	3694	42925	2280	4028	44838	1458	2937	44824	793	1394	48872	226	135
Person years	7189483.35	720428.3	437816	7997213.07	163306.17	187208.41	8141949.09	92665.97	113112.58	8068421.51	71849.18	131691.25	8295715.68	32168.59	19843.36
Incidence rate/1000 person years	5.77	5.67	8.44	5.37	13.96	21.52	5.51	15.73	25.97	5.56	11.04	10.59	5.89	7.03	6.8
	**Type 2 diabetes**														
n	1206838	126825	78220	1332890	30810	48183	1358997	18744	34142	1358544	18626	34713	1402208	5636	4039
Events	18276	1872	1606	18812	958	1984	19510	628	1616	17375	1484	2895	21565	130	59
Person years	7467660	746088.1	461975.7	8189689	194493.2	291541.4	8359628	114370.6	201724.6	8469777	73149.23	132797.5	8620463	34183.46	21077.03
Incidence rate/1000 person years	2.45	2.51	3.48	2.3	4.93	6.81	2.33	5.49	8.01	2.05	20.29	21.8	2.5	3.8	2.8
	**Cardiovascular disease (CVD)**														
n	1215276	127916	79095	1340875	31474	49938	1367284	19256	35747	1349280	18477	34431	1412520	5687	4080
Events	13550	1334	1185	14327	478	1264	14721	318	1030	14416	181	316	15939	93	37
Person years	7548979	754128.2	469248.8	8266378	200379.4	305598.1	8439611	118611.4	214133.2	8435736	78728.52	144315	8716289	34640.38	21426.49
Incidence rate/1000 person years	1.79	1.77	2.53	1.73	2.39	4.14	1.74	2.68	4.81	1.71	2.3	2.19	1.83	2.68	1.73
	**Chronic Kidney Disease (CKD)**														
n	1222489	128792	79684	1348849	31735	50381	1375497	19408	36060	1357128	18609	34678	1421121	5741	4103
Events	4462	417	409	4430	201	657	4559	146	583	4553	46	78	5243	26	19
Person years	7626976	761992.5	475389.1	8351469	202589.2	310299.4	8527162	119909.5	217286.5	8520251	79738.98	145975.4	8807637	35147.96	21573.37

Incidence rate/1000 person years	0.59	0.55	0.86	0.53	0.99	2.12	0.53	1.22	2.68	0.53	0.58	0.53	0.6	0.74	0.88

Incidence rates of hypertension, type 2 diabetes mellitus, cardiovascular disease, and chronic kidney disease among women stratified by pregnancy complication history (no history, previous history, or latest pregnancy affected). Rates are presented per 1000 person-years of follow-up. **Abbreviations:** CKD, chronic kidney disease; CVD, cardiovascular disease; T2DM, type 2 diabetes mellitus.

Table 6 (continued): Incidence rates for hypertension, type 2 diabetes, cardiovascular disease and chronic kidney disease in women with latest, previous and without a history of pregnancy complications

	**Stillbirth**			**Preterm birth**			**SGA**			**Postnatal depression**		
Variable	**No history**	**Previous**	**Latest**	**No history**	**Previous**	**Latest**	**No history**	**Previous**	**Latest**	**No history**	**Previous**	**Latest**
	**Hypertension**											
n	1375223	11101	3136	1342122	24633	22705	1334625	21211	33624	1163203	44731	181526
Events	48470	645	118	48098	674	461	47430	778	1025	39042	1687	8504
Person years	8266167.74	67209.68	14350.22	8151701.03	104540.52	91486.09	8044673.46	122122.91	180931.27	7120064.06	246821.94	980841.64
Incidence rate/1000 person years	5.86	9.6	8.22	5.9	6.45	5.04	5.9	6.37	5.67	5.48	6.83	8.67
	**Type 2 diabetes**											
n	1397320	11386	3177	1363953	25024	22906	1355544	21881	34458	1180321	45555	186007
Events	21300	373	81	21114	383	257	21049	336	369	16848	794	4112
Person years	8589962.6	70823.61	14937.39	8473396.3	108368.7	93958.61	8357102.3	128474.09	190147.3	7383066.09	256618.25	1036039.3
Incidence rate/1000 person years	2.48	5.27	5.42	2.49	3.53	2.74	2.52	2.62	1.94	2.28	3.09	3.97
	**Cardiovascular disease (CVD)**											
n	1407269	11762	3256	1373643	25383	23261	1365831	21968	34488	1188078	46018	188191
Events	15790	246	33	15628	243	198	15475	239	355	11912	691	3466
Person years	8682616.5	74168.21	15570.79	8566316.9	110530.51	95508.11	8452663.9	129196.31	190495.3	7458792.49	260102.66	1053460.4

Incidence rate/1000 person years	1.82	3.32	2.12	1.82	2.2	2.07	1.83	1.85	1.86	1.6	2.66	3.29
	**Chronic Kidney Disease (CKD)**											
n	1415805	11872	3288	1381965	25609	23391	1374160	22129	34676	1194076	46444	190445
Events	5190	75	23	5163	88	37	5123	61	104	4087	172	1029
Person years	8773214.1	75333.3	15810.54	8656089.1	111769.74	96499.11	8541273.4	130735.71	192348.88	7525937.78	263902	1074518.2
Incidence rate/1000 person years	0.59	1	1.45	0.6	0.79	0.38	0.6	0.47	0.54	0.54	0.65	0.96

Incidence rates of hypertension, type 2 diabetes mellitus, cardiovascular disease, and chronic kidney disease among women stratified by pregnancy complication history (no history, previous history, or latest pregnancy affected). Rates are presented per 1000 person-years of follow-up. **Abbreviations:** CKD, chronic kidney disease; CVD, cardiovascular disease; T2DM, type 2 diabetes mellitus.

Table 6 (continued): Incidence rates for type 2 diabetes in women with latest, previous and without a history of LGA

	**LGA**		
	**No history**	**Previous**	**Latest**
n	1215416	51660	144807
Events	18134	1300	2320
Person years	7548401.57	316396.35	810925.73
Incidence rate/1000 person years	2.4	4.11	2.86

Incidence rates of hypertension, type 2 diabetes mellitus, cardiovascular disease, and chronic kidney disease among women stratified by pregnancy complication history (no history, previous history, or latest pregnancy affected). Rates are presented per 1000 person-years of follow-up. **Abbreviations:** CKD, chronic kidney disease; CVD, cardiovascular disease; T2DM, type 2 diabetes mellitus.

Table 7: Adjustment factors in each regression model

	**Hypertension outcome**	**T2DM outcome**	**CVD outcome**	**CKD outcome**
Miscarriage	Age, Ethnicity, social deprivation, smoking status, BMI, Atypical antipsychotic medications, corticosteroids medication, Atrial fibrillation, Migraine, chronic kidney disease (CKD), Rheumatoid arthritis, severe mental illness, pregnancy year, gravidity, family history of CVD, CVD, diabetes mellitus	Age, Ethnicity, social deprivation, smoking status, BMI, SBP, Atypical antipsychotic medications, corticosteroids medication, Antihypertensives, statins, Atrial fibrillation, Migraine, learning disability, chronic kidney disease (CKD), Rheumatoid arthritis, severe mental illness, pregnancy year, gravidity, family history of CVD, CVD, hypertension	Age, Ethnicity, social deprivation, smoking status, BMI, SBP, Atypical antipsychotic medications, corticosteroids medication, Antihypertensives, Atrial fibrillation, Migraine, chronic kidney disease (CKD), Rheumatoid arthritis, severe mental illness, pregnancy year, gravidity, family history of CVD, hypertension, diabetes mellitus	Age, Ethnicity, social deprivation, smoking status, alcohol misuse, BMI, SBP, Atypical antipsychotic medications, corticosteroids medication, Antihypertensives, Atrial fibrillation, Migraine, Rheumatoid arthritis, severe mental illness, pregnancy year, gravidity, family history of CVD, CVD, diabetes, hypertension
Gestational hypertension	Age, Ethnicity, social deprivation, smoking status, BMI, Atypical antipsychotic medications, corticosteroids medication, Atrial fibrillation, Migraine, chronic kidney disease (CKD), Rheumatoid arthritis, severe mental illness, pregnancy year, gravidity, family history of CVD, CVD, diabetes mellitus	Age, Ethnicity, social deprivation, smoking status, BMI, SBP, Atypical antipsychotic medications, corticosteroids medication, statins, Atrial fibrillation, Migraine, learning disability, chronic kidney disease (CKD), Rheumatoid arthritis, severe mental illness, pregnancy year, gravidity, family history of CVD, CVD	Age, Ethnicity, social deprivation, smoking status, BMI, SBP, Atypical antipsychotic medications, corticosteroids medication, Atrial fibrillation, Migraine, chronic kidney disease (CKD), Rheumatoid arthritis, severe mental illness, pregnancy year,	Age, Ethnicity, social deprivation, smoking status, alcohol misuse, BMI, SBP, Atypical antipsychotic medications, corticosteroids medication, Atrial fibrillation, Migraine, Rheumatoid arthritis, severe mental illness, pregnancy year, gravidity, family history of CVD, CVD, diabetes

			gravidity, family history of CVD, diabetes mellitus	
Pre-eclampsia	Age, Ethnicity, social deprivation, smoking status, BMI, Atypical antipsychotic medications, corticosteroids medication, Atrial fibrillation, Migraine, chronic kidney disease (CKD), Rheumatoid arthritis, severe mental illness, pregnancy year, gravidity, family history of CVD, CVD, diabetes mellitus	Age, Ethnicity, social deprivation, smoking status, BMI, SBP, Atypical antipsychotic medications, corticosteroids medication, statins, Atrial fibrillation, Migraine, learning disability, chronic kidney disease (CKD), Rheumatoid arthritis, severe mental illness, pregnancy year, gravidity, family history of CVD, CVD	Age, Ethnicity, social deprivation, smoking status, BMI, SBP, Atypical antipsychotic medications, corticosteroids medication, Atrial fibrillation, Migraine, chronic kidney disease (CKD), Rheumatoid arthritis, severe mental illness, pregnancy year, gravidity, family history of CVD, diabetes mellitus	Age, Ethnicity, social deprivation, smoking status, alcohol misuse, BMI, SBP, Atypical antipsychotic medications, corticosteroids medication, Atrial fibrillation, Migraine, Rheumatoid arthritis, severe mental illness, pregnancy year, gravidity, family history of CVD, CVD, diabetes
GDM	Age, Ethnicity, social deprivation, smoking status, BMI, Atypical antipsychotic medications, corticosteroids medication, Atrial fibrillation, Migraine, chronic kidney disease (CKD), Rheumatoid arthritis, severe mental illness, pregnancy year, gravidity, family history of CVD, CVD	Age, Ethnicity, social deprivation, smoking status, BMI, SBP, Atypical antipsychotic medications, corticosteroids medication, Antihypertensives, statins, Atrial fibrillation, Migraine, learning disability, chronic kidney disease (CKD), Rheumatoid arthritis, severe mental illness, pregnancy year, gravidity, family history of CVD, CVD, hypertension	Age, Ethnicity, social deprivation, smoking status, BMI, SBP, Atypical antipsychotic medications, corticosteroids medication, Atrial fibrillation, Migraine, chronic kidney disease (CKD), Rheumatoid arthritis, severe mental illness, pregnancy year, gravidity, family history of CVD	Age, Ethnicity, social deprivation, smoking status, alcohol misuse, BMI, SBP, Atypical antipsychotic medications, corticosteroids medication, Antihypertensives, Atrial fibrillation, Migraine, Rheumatoid arthritis, severe mental illness, pregnancy year, gravidity, family history of CVD, CVD, hypertension

Placental abruption	Age, Ethnicity, social deprivation, smoking status, BMI, Atypical antipsychotic medications, corticosteroids medication, Atrial fibrillation, Migraine, chronic kidney disease (CKD), Rheumatoid arthritis, severe mental illness, pregnancy year, gravidity, family history of CVD, CVD, diabetes mellitus, Pre-eclampsia	Age, Ethnicity, social deprivation, smoking status, BMI, SBP, Atypical antipsychotic medications, corticosteroids medication, Antihypertensives, statins, Atrial fibrillation, Migraine, learning disability, chronic kidney disease (CKD), Rheumatoid arthritis, severe mental illness, pregnancy year, gravidity, family history of CVD, CVD, hypertension, Pre-eclampsia	Age, Ethnicity, social deprivation, smoking status, BMI, SBP, Atypical antipsychotic medications, corticosteroids medication, Antihypertensives, Atrial fibrillation, Migraine, chronic kidney disease (CKD), Rheumatoid arthritis, severe mental illness, pregnancy year, gravidity, family history of CVD, hypertension, diabetes mellitus, Pre-eclampsia	Age, Ethnicity, social deprivation, smoking status, alcohol misuse, BMI, SBP, Atypical antipsychotic medications, corticosteroids medication, Antihypertensives, Atrial fibrillation, Migraine, Rheumatoid arthritis, severe mental illness, pregnancy year, gravidity, family history of CVD, CVD, diabetes, hypertension, Pre-eclampsia
Stillbirth	Age, Ethnicity, social deprivation, smoking status, BMI, Atypical antipsychotic medications, corticosteroids medication, Atrial fibrillation, Migraine, chronic kidney disease (CKD), Rheumatoid arthritis, severe mental illness, pregnancy year, gravidity, family history of CVD, CVD, diabetes mellitus, Pre-eclampsia	Age, Ethnicity, social deprivation, smoking status, BMI, SBP, Atypical antipsychotic medications, corticosteroids medication, Antihypertensives, statins, Atrial fibrillation, Migraine, learning disability, chronic kidney disease (CKD), Rheumatoid arthritis, severe mental illness, pregnancy year, gravidity, family history	Age, Ethnicity, social deprivation, smoking status, BMI, SBP, Atypical antipsychotic medications, corticosteroids medication, Antihypertensives, Atrial fibrillation, Migraine, chronic kidney disease (CKD), Rheumatoid arthritis, severe mental illness, pregnancy year,	Age, Ethnicity, social deprivation, smoking status, alcohol misuse, BMI, SBP, Atypical antipsychotic medications, corticosteroids medication, Antihypertensives, Atrial fibrillation, Migraine, Rheumatoid arthritis, severe mental illness, pregnancy year, gravidity, family history of CVD, CVD, diabetes, hypertension, Pre-eclampsia

		of CVD, CVD, hypertension, Pre-eclampsia	gravidity, family history of CVD, hypertension, diabetes mellitus, Pre-eclampsia	
Preterm birth	Age, Ethnicity, social deprivation, smoking status, BMI, Atypical antipsychotic medications, corticosteroids medication, Atrial fibrillation, Migraine, chronic kidney disease (CKD), Rheumatoid arthritis, severe mental illness, pregnancy year, gravidity, family history of CVD, CVD, diabetes mellitus, Pre-eclampsia	Age, Ethnicity, social deprivation, smoking status, BMI, SBP, Atypical antipsychotic medications, corticosteroids medication, Antihypertensives, statins, Atrial fibrillation, Migraine, learning disability, chronic kidney disease (CKD), Rheumatoid arthritis, severe mental illness, pregnancy year, gravidity, family history of CVD, CVD, hypertension, Pre-eclampsia	Age, Ethnicity, social deprivation, smoking status, BMI, SBP, Atypical antipsychotic medications, corticosteroids medication, Antihypertensives, Atrial fibrillation, Migraine, chronic kidney disease (CKD), Rheumatoid arthritis, severe mental illness, pregnancy year, gravidity, family history of CVD, hypertension, diabetes mellitus, Pre-eclampsia	Age, Ethnicity, social deprivation, smoking status, alcohol misuse, BMI, SBP, Atypical antipsychotic medications, corticosteroids medication, Antihypertensives, Atrial fibrillation, Migraine, Rheumatoid arthritis, severe mental illness, pregnancy year, gravidity, family history of CVD, CVD, diabetes, hypertension, Pre-eclampsia
SGA	Age, Ethnicity, social deprivation, smoking status, BMI, Atypical antipsychotic medications, corticosteroids medication, Atrial fibrillation, Migraine, chronic kidney disease (CKD), Rheumatoid arthritis, severe mental illness, pregnancy year, gravidity, family history of CVD,	Age, Ethnicity, social deprivation, smoking status, BMI, SBP, Atypical antipsychotic medications, corticosteroids medication, Antihypertensives, statins, Atrial fibrillation, Migraine, learning disability, chronic kidney disease (CKD),	Age, Ethnicity, social deprivation, smoking status, BMI, SBP, Atypical antipsychotic medications, corticosteroids medication, Antihypertensives, Atrial fibrillation, Migraine,	Age, Ethnicity, social deprivation, smoking status, alcohol misuse, BMI, SBP, Atypical antipsychotic medications, corticosteroids medication, Antihypertensives, Atrial fibrillation, Migraine, Rheumatoid arthritis, severe

	CVD, diabetes mellitus, Pre-eclampsia	Rheumatoid arthritis, severe mental illness, pregnancy year, gravidity, family history of CVD, CVD, hypertension, Pre-eclampsia	chronic kidney disease (CKD), Rheumatoid arthritis, severe mental illness, pregnancy year, gravidity, family history of CVD, hypertension, diabetes mellitus, Pre-eclampsia	mental illness, pregnancy year, gravidity, family history of CVD, CVD, diabetes, hypertension, Pre-eclampsia
Postnatal depression	Age, Ethnicity, social deprivation, smoking status, BMI, Atypical antipsychotic medications, corticosteroids medication, Atrial fibrillation, Migraine, chronic kidney disease (CKD), Rheumatoid arthritis, severe mental illness, pregnancy year, gravidity, family history of CVD, CVD, diabetes mellitus	Age, Ethnicity, social deprivation, smoking status, BMI, SBP, Atypical antipsychotic medications, corticosteroids medication, Antihypertensives, statins, Atrial fibrillation, Migraine, learning disability, chronic kidney disease (CKD), Rheumatoid arthritis, severe mental illness, pregnancy year, gravidity, family history of CVD, CVD, hypertension	Age, Ethnicity, social deprivation, smoking status, BMI, SBP, Atypical antipsychotic medications, corticosteroids medication, Antihypertensives, Atrial fibrillation, Migraine, chronic kidney disease (CKD), Rheumatoid arthritis, severe mental illness, pregnancy year, gravidity, family history of CVD, hypertension, diabetes mellitus	Age, Ethnicity, social deprivation, smoking status, alcohol misuse, BMI, SBP, Atypical antipsychotic medications, corticosteroids medication, Antihypertensives, Atrial fibrillation, Migraine, Rheumatoid arthritis, severe mental illness, pregnancy year, gravidity, family history of CVD, CVD, diabetes, hypertension

Covariates included in adjusted regression models assessing the association between pregnancy complications and cardiometabolic outcomes. Models include demographic, socioeconomic, lifestyle, reproductive, medication, and clinical factors, selected a priori based on clinical relevance and directed acyclic graphs (DAGs). **Abbreviations:** BMI, body mass index; CVD, cardiovascular disease; IMD, Index of Multiple Deprivation.

Figure 1a: DAG for the association between pregnancy complications and cardiovascular disease.

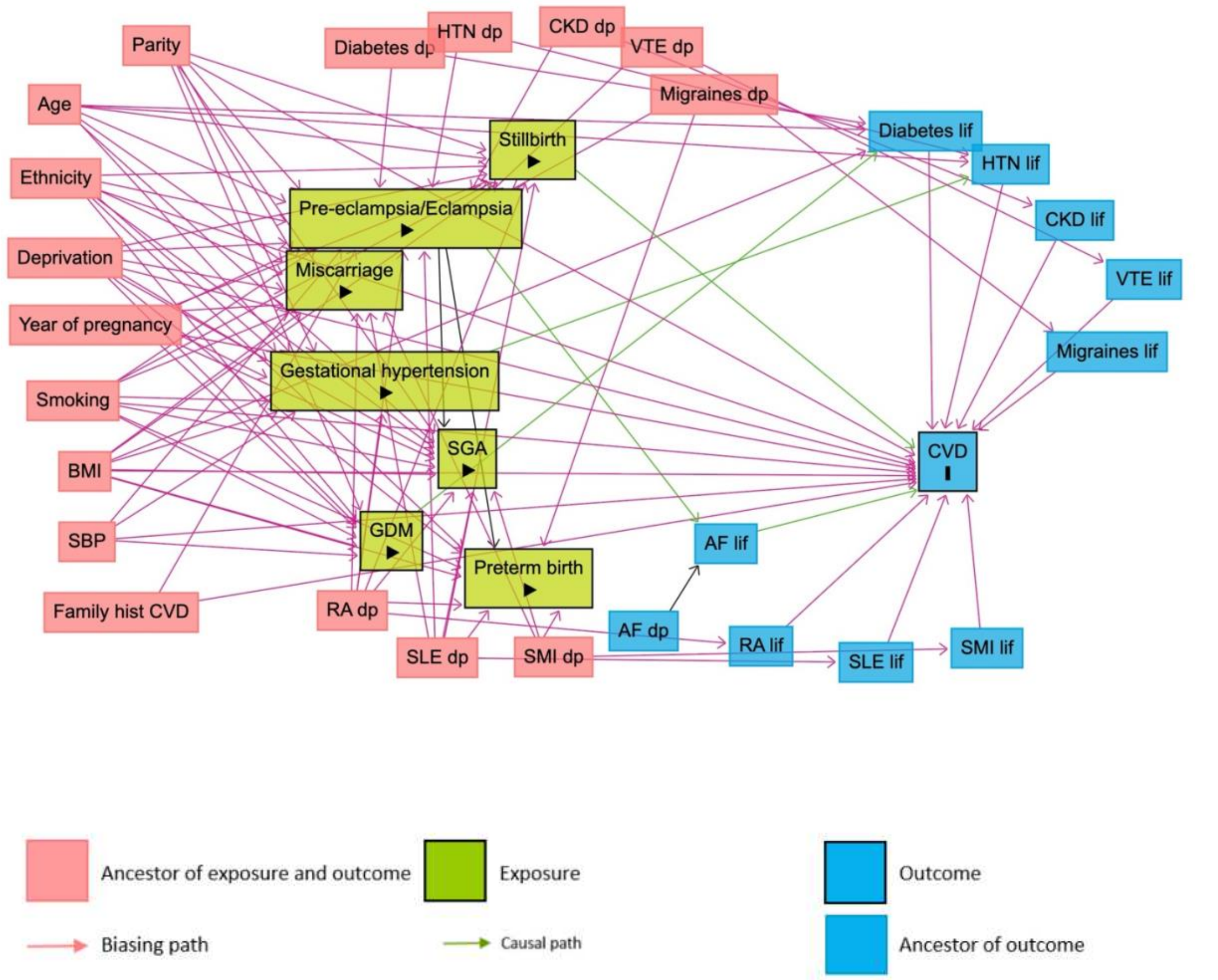

BMI-Body Mass Index , **SBP** – Systolic Blood Pressure, **CVD** – Cardiovascular disease, **HTN** – Hypertension, **CKD** – Chronic Kidney Disease, **VTE** – Venous Thromboembolism, **SGA** – Small for Gestational Age, **GDM** – Gestational Diabetes Mellitus, **RA**- Rheumatoid Arthritis, **SLE** - Systemic Lupus Erythematosus, **SMI** – Severe Mental Illness, **AF** – Atrial Fibrillation

dp – during pregnancy, **lif**- later in life

The DAG was developed by the authors using DAGitty, with the selection of some variables and causal relationships informed by Täufer Cederlöf et al (Täufer Cederlöf E, Lundgren M, Lindahl B, Christersson C. Pregnancy complications and risk of cardiovascular disease later in life: a nationwide cohort study. J Am Heart Assoc. 2022;11(2):e023079. doi:10.1161/JAHA.121.023079)

Figure 1b: DAG for association between pregnancy complications and type 2 diabetes (T2DM)

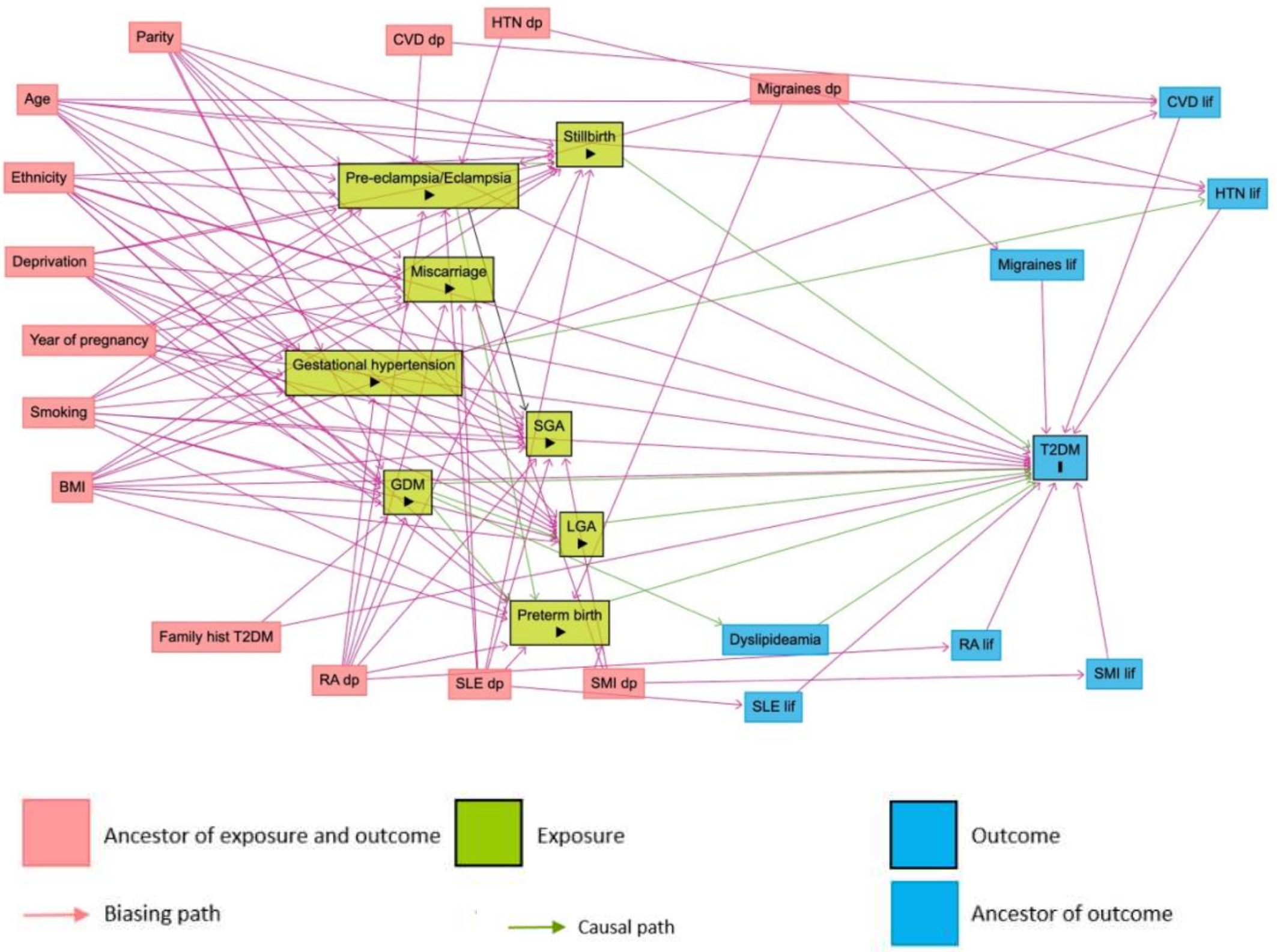

BMI-Body Mass Index , **SBP** – Systolic Blood Pressure, **CVD** – Cardiovascular disease, **HTN** – Hypertension, **CKD** – Chronic Kidney Disease, **VTE** – Venous Thromboembolism, **SGA** – Small for Gestational Age, **GDM** – Gestational Diabetes Mellitus, **RA**- Rheumatoid Arthritis, **SLE** - Systemic Lupus Erythematosus, **SMI** – Severe Mental Illness, **AF** – Atrial Fibrillation

dp – during pregnancy, **lif**- later in life

Figure 1c: DAG for association between pregnancy complications and hypertension

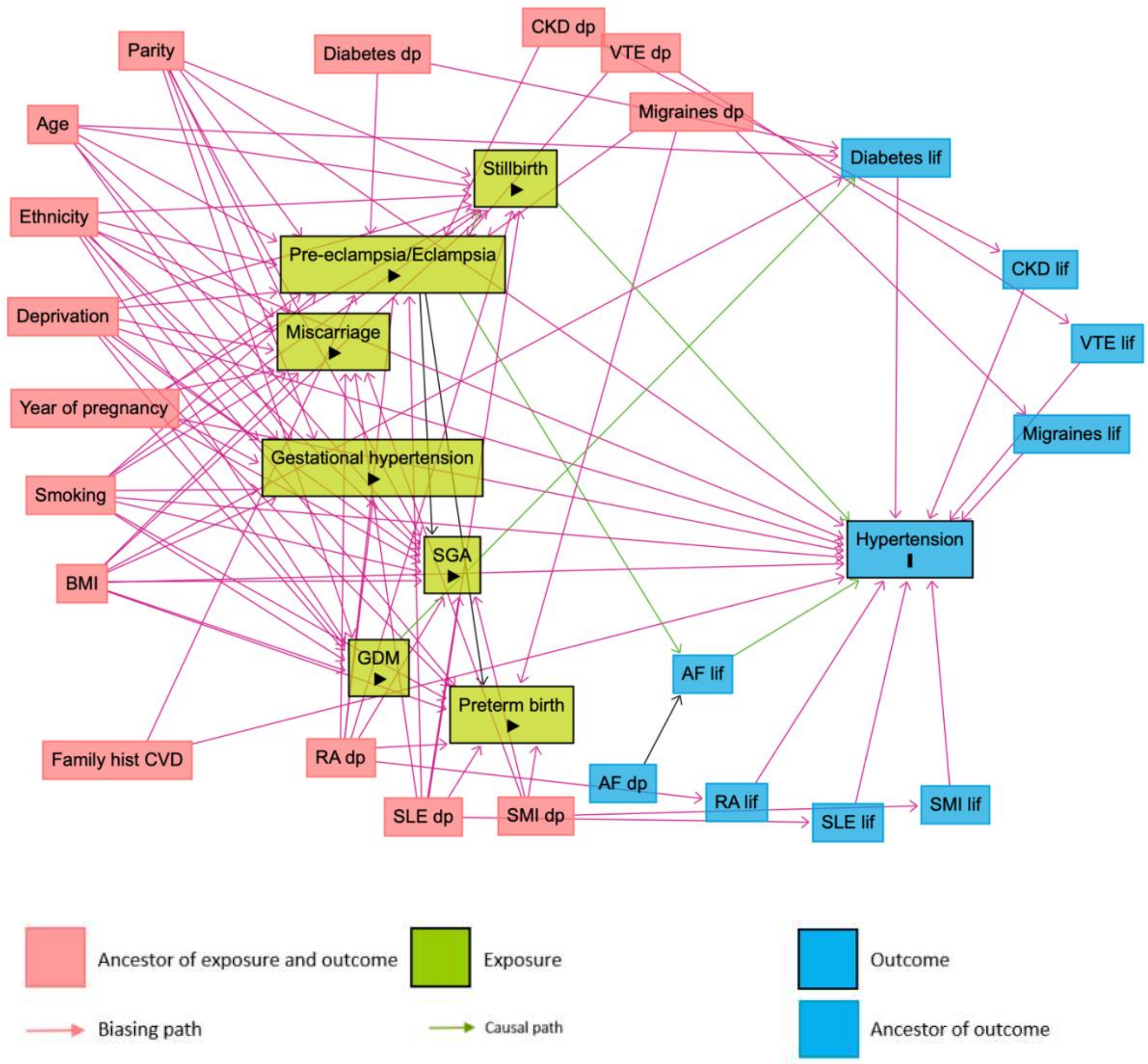

BMI-Body Mass Index , **SBP** – Systolic Blood Pressure, **CVD** – Cardiovascular disease, **HTN** – Hypertension, **CKD** – Chronic Kidney Disease, **VTE** – Venous Thromboembolism, **SGA** – Small for Gestational Age, **GDM** – Gestational Diabetes Mellitus, **RA**- Rheumatoid Arthritis, **SLE** - Systemic Lupus Erythematosus, **SMI** – Severe Mental Illness, **AF** – Atrial Fibrillation

dp – during pregnancy, **lif**- later in life

Figure 1d: DAG for association between pregnancy complications and chronic kidney disease

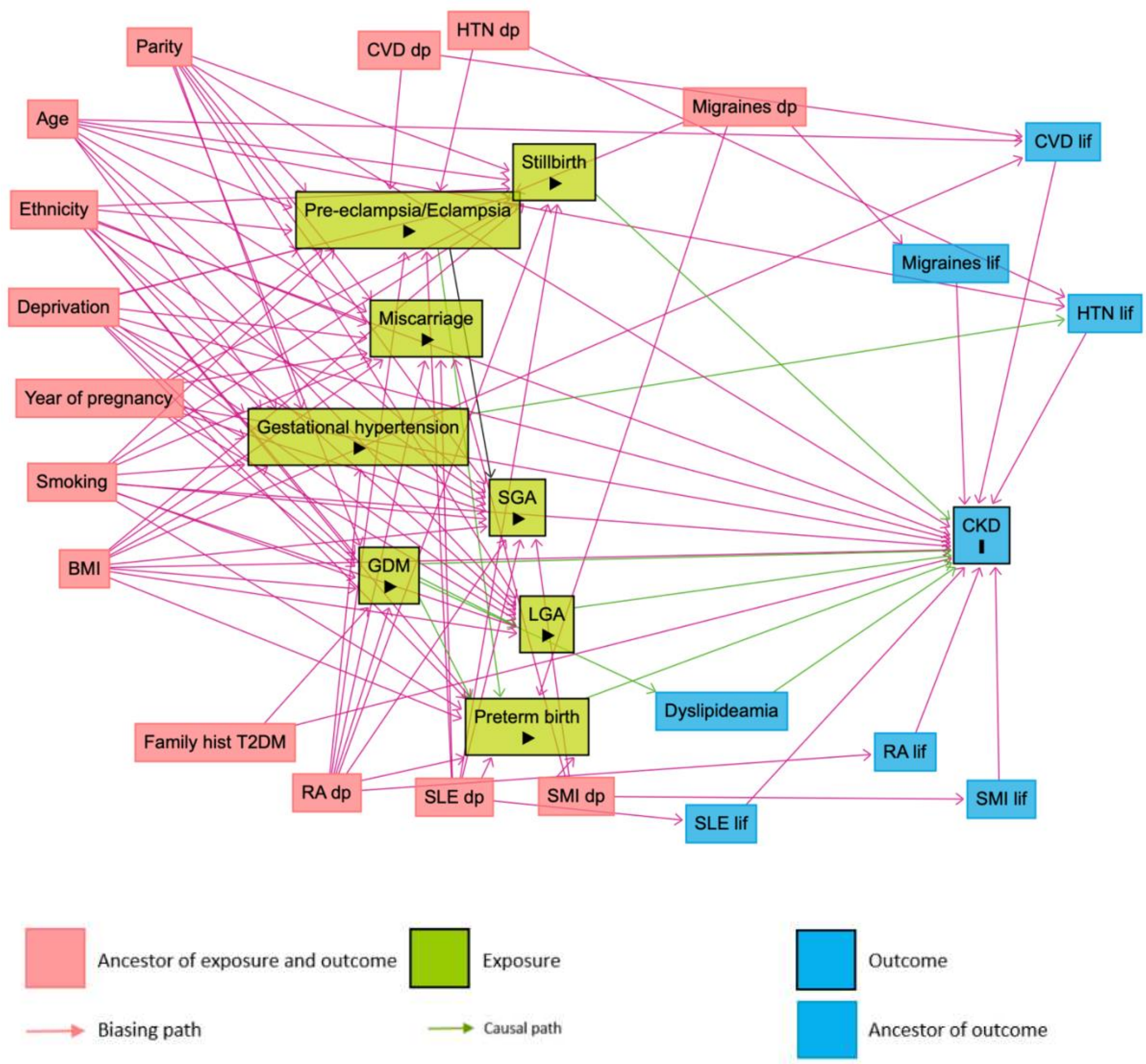

BMI-Body Mass Index , **SBP** – Systolic Blood Pressure, **CVD** – Cardiovascular disease, **HTN** – Hypertension, **CKD** – Chronic Kidney Disease, **VTE** – Venous Thromboembolism, **SGA** – Small for Gestational Age, **GDM** – Gestational Diabetes Mellitus, **RA**- Rheumatoid Arthritis, **SLE** - Systemic Lupus Erythematosus, **SMI** – Severe Mental Illness, **AF** – Atrial Fibrillation

dp – during pregnancy, **lif**- later in life

Unadjusted Hazard Ratios

Figure 2: Unadjusted hazard ratios for hypertension outcome

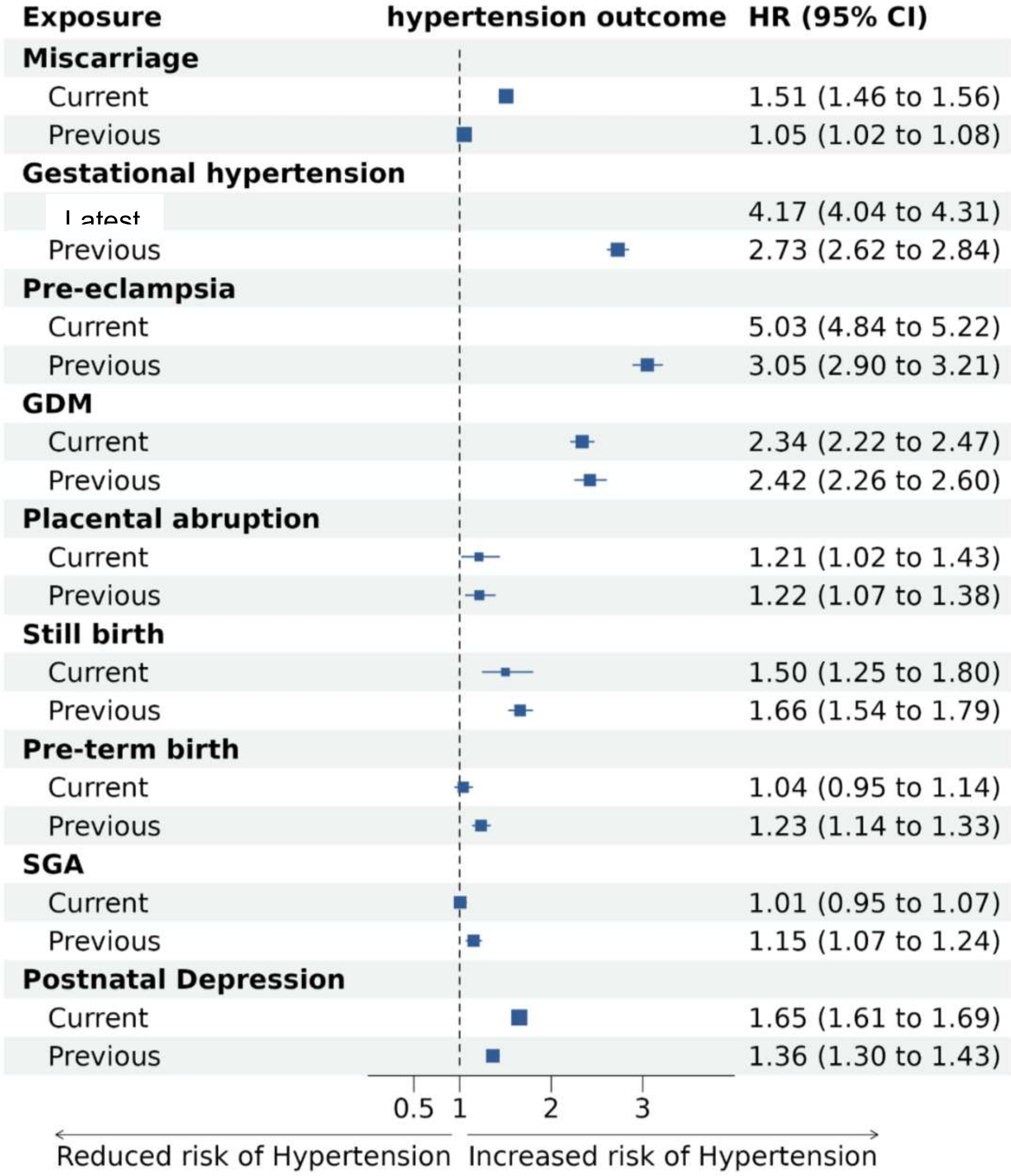

Figure 3: Unadjusted hazard ratios for type 2 diabetes outcome

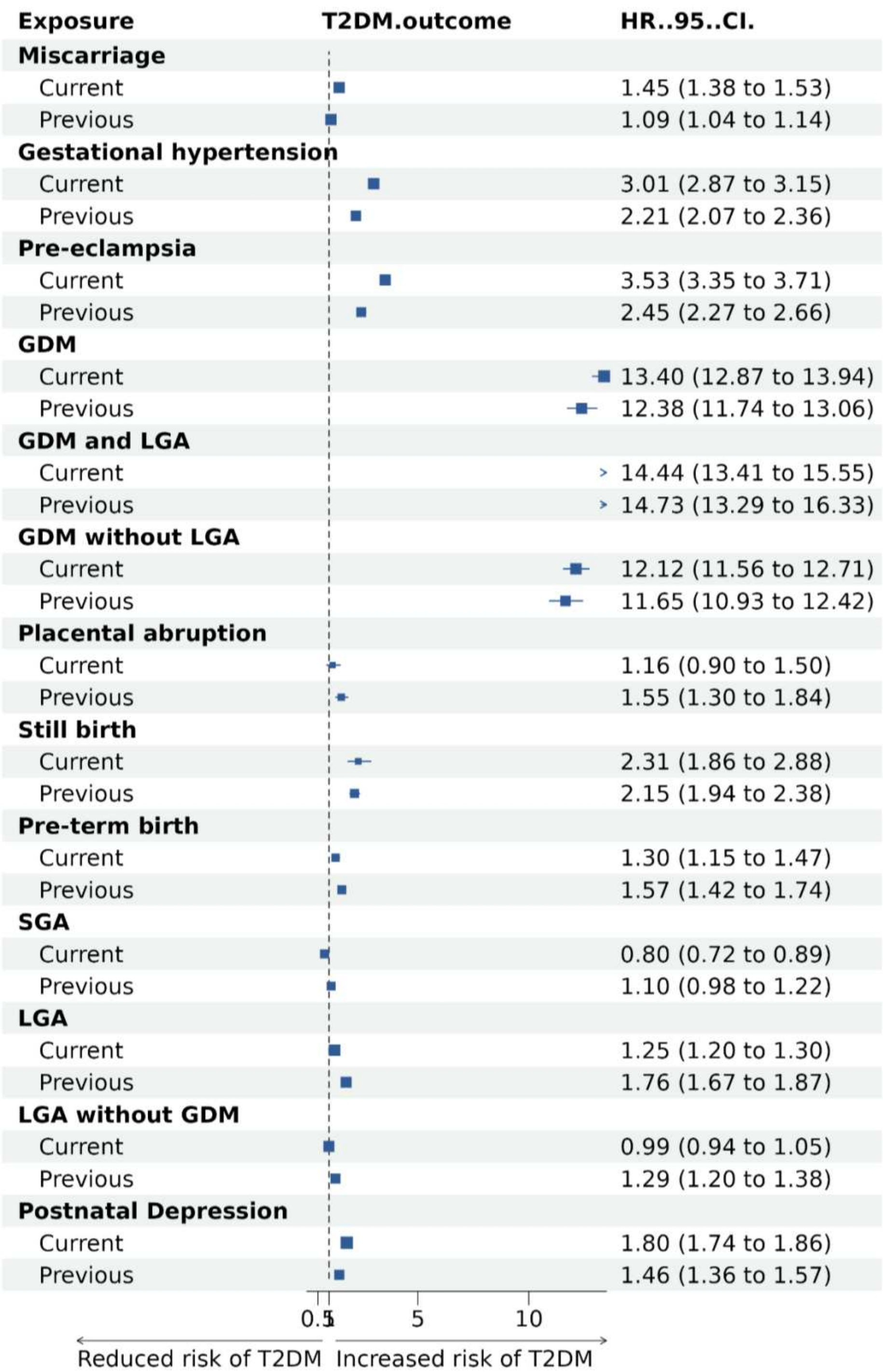

Figure 4: Unadjusted hazard ratios for CVD outcome

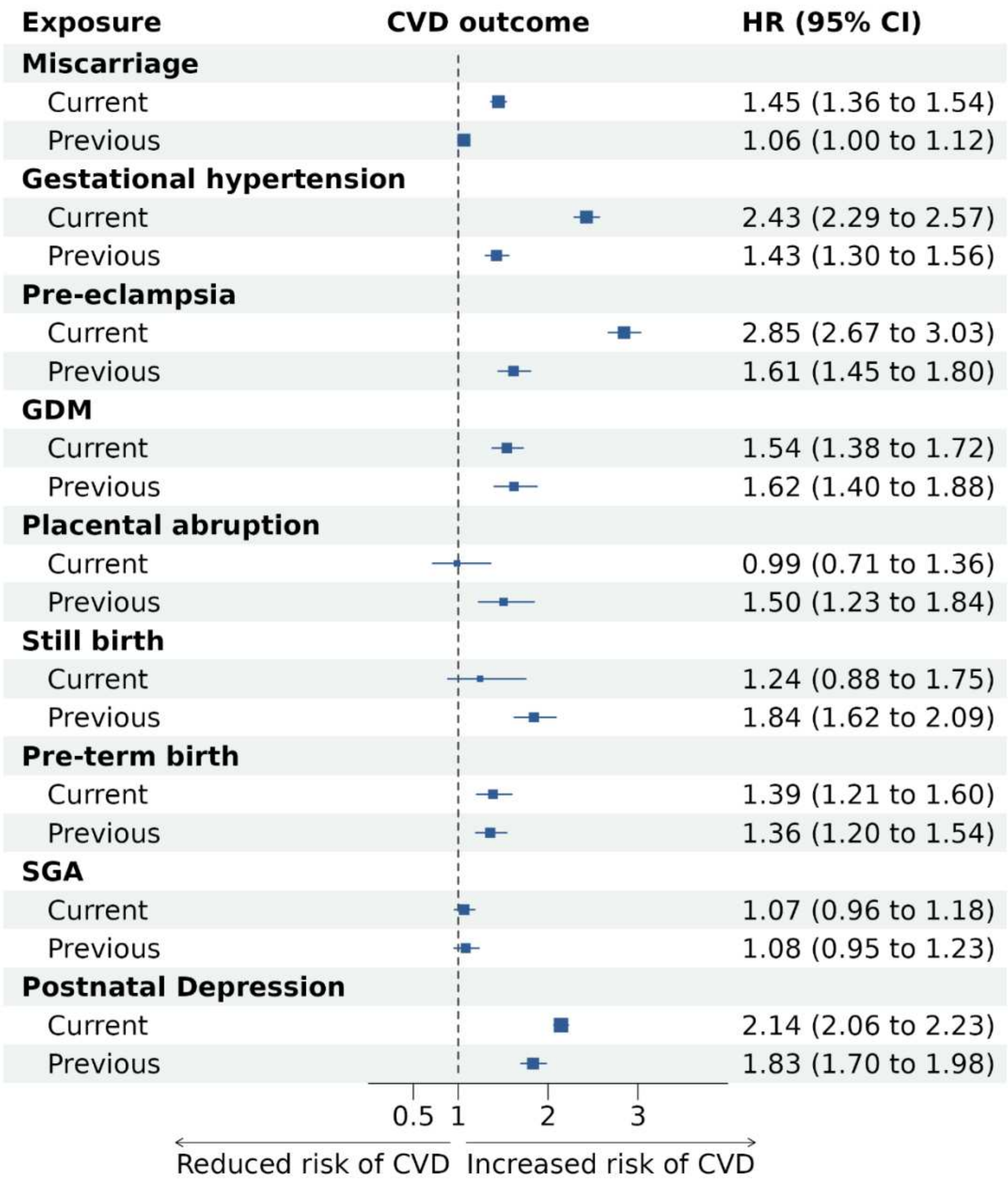

Figure 5: Unadjusted hazard ratios for CKD outcome

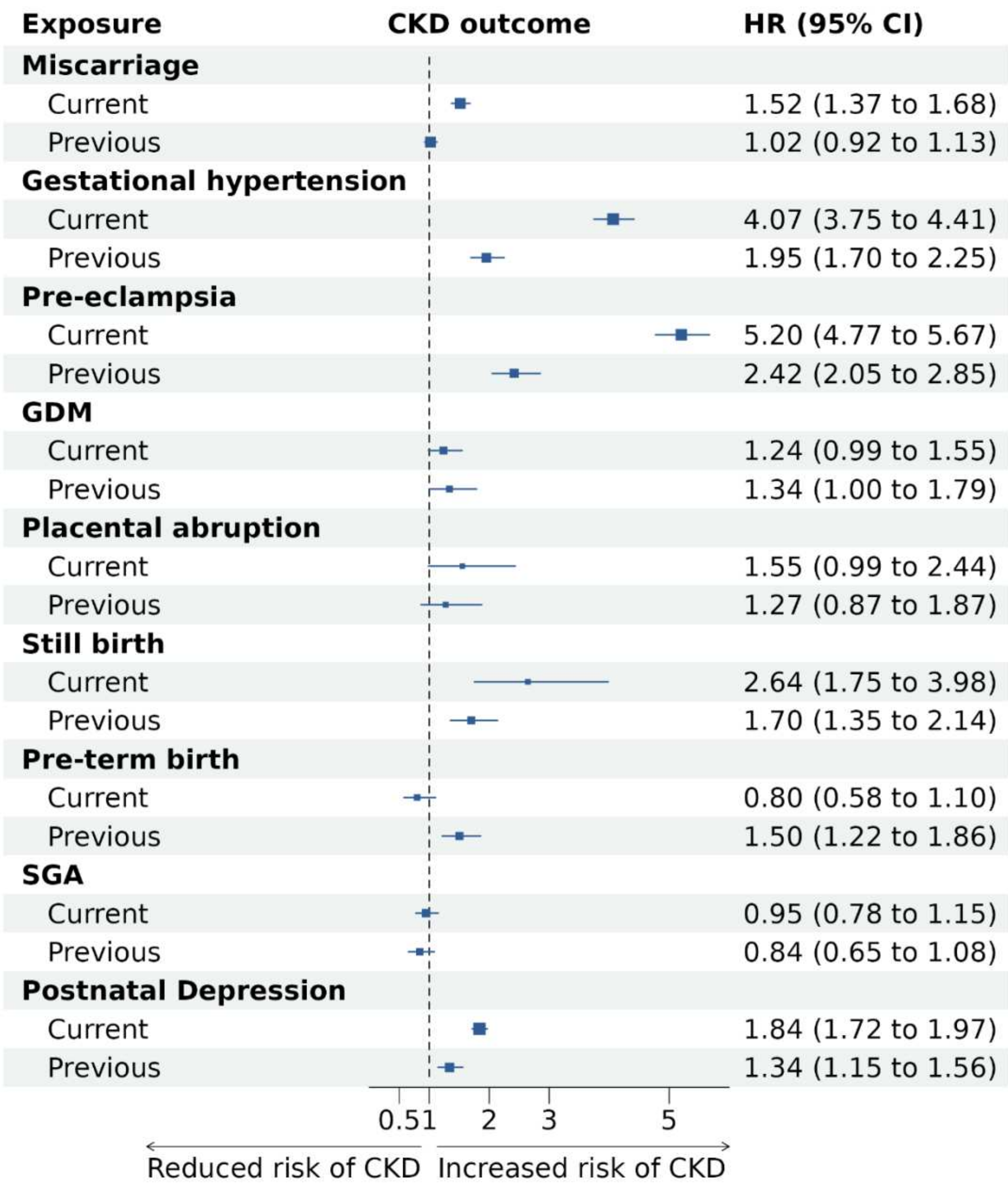

Sensitivity analysis after adding time interaction to variables violating the Cox PH Assumption

Figure 6: Adjusted hazard ratios for CVD

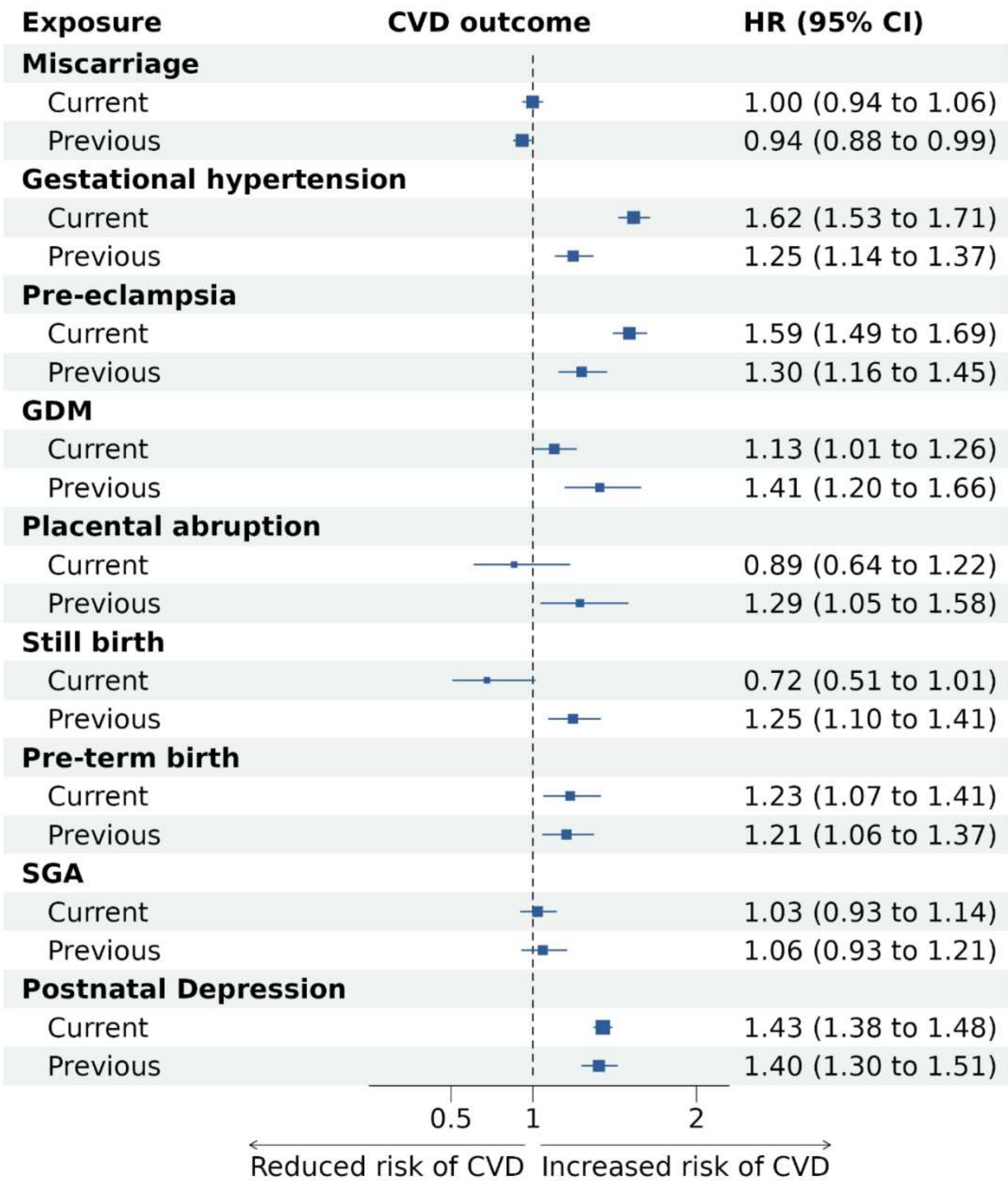

Figure 7: Adjusted hazard ratios for CKD

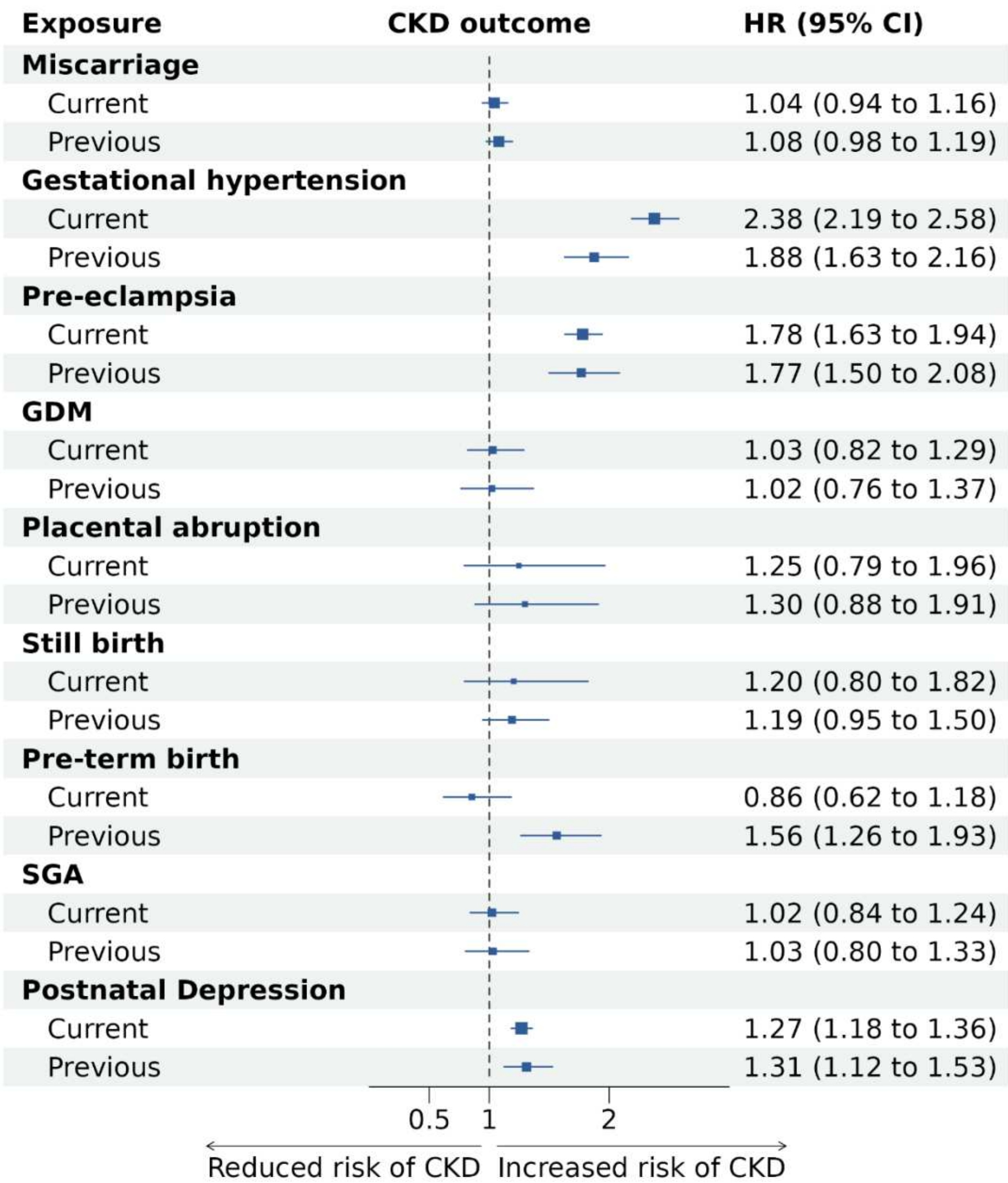

Multiple testing adjustment

Table 8: P-values adjusted for multiple testing

Exposure group	Outcome	P-value from multivariable model before adjustment for multiple testing	P-value from multivariable model after adjustment for multiple testing
Latest miscarriage	Hypertension	0.0001	0.0003
Latest miscarriage	Type 2 diabetes	0.1007	0.1007
Latest miscarriage	Cardiovascular disease	0.0224	0.0447
Latest miscarriage	Chronic Kidney Disease	0.0370	0.0494
Previous miscarriage	Hypertension	0.2152	0.2870
Previous miscarriage	Type 2 diabetes	0.7505	0.7505
Previous miscarriage	Cardiovascular disease	0.1062	0.2123
Previous miscarriage	Chronic Kidney Disease	**0.0314**	**0.1256**
Latest Gestational hypertension	Hypertension	< 0.0001	< 0.0001
Latest Gestational hypertension	Type 2 diabetes	< 0.0001	< 0.0001
Latest Gestational hypertension	Cardiovascular disease	< 0.0001	< 0.0001
Latest Gestational hypertension	Chronic Kidney Disease	< 0.0001	< 0.0001
Previous Gestational hypertension	Hypertension	< 0.0001	< 0.0001
Previous Gestational hypertension	Type 2 diabetes	< 0.0001	< 0.0001
Previous Gestational hypertension	Cardiovascular disease	0.0003	0.0003
Previous Gestational hypertension	Chronic Kidney Disease	< 0.0001	< 0.0001
Latest Pre-eclampsia	Hypertension	< 0.0001	< 0.0001
Latest Pre-eclampsia	Type 2 diabetes	< 0.0001	< 0.0001
Latest Pre-eclampsia	Cardiovascular disease	< 0.0001	< 0.0001
Latest Pre-eclampsia	Chronic Kidney Disease	< 0.0001	< 0.0001
Previous Pre-eclampsia	Hypertension	< 0.0001	< 0.0001
Previous Pre-eclampsia	Type 2 diabetes	< 0.0001	< 0.0001
Previous Pre-eclampsia	Cardiovascular disease	0.0001	0.0001
Previous Pre-eclampsia	Chronic Kidney Disease	< 0.0001	< 0.0001
Latest GDM	Hypertension	< 0.0001	< 0.0001
Latest GDM	Type 2 diabetes	< 0.0001	< 0.0001
Latest GDM	Cardiovascular disease	< 0.0001	< 0.0001

Latest GDM	Chronic Kidney Disease	0.6138	0.6138
Previous GDM	Hypertension	< 0.0001	< 0.0001
Previous GDM	Type 2 diabetes	< 0.0001	< 0.0001
Previous GDM	Cardiovascular disease	0.4404	0.5871
Previous GDM	Chronic Kidney Disease	0.6841	0.6841
Latest Placental abruption	Hypertension	< 0.0001	< 0.0001
Latest Placental abruption	Type 2 diabetes	0.0132	0.0197
Latest Placental abruption	Cardiovascular disease	0.6661	0.6661
Latest Placental abruption	Chronic Kidney Disease	0.0148	0.0197
Previous Placental abruption	Hypertension	0.0789	0.1052
Previous Placental abruption	Type 2 diabetes	0.0738	0.1052
Previous Placental abruption	Cardiovascular disease	**0.0252**	**0.1010**
Previous Placental abruption	Chronic Kidney Disease	0.2946	0.2946
Latest Stillbirth	Hypertension	0.1313	0.1750
Latest Stillbirth	Type 2 diabetes	< 0.0001	< 0.0001
Latest Stillbirth	Cardiovascular disease	0.8375	0.8375
Latest Stillbirth	Chronic Kidney Disease	0.0035	0.0070
Previous Stillbirth	Hypertension	< 0.0001	< 0.0001
Previous Stillbirth	Type 2 diabetes	< 0.0001	< 0.0001
Previous Stillbirth	Cardiovascular disease	< 0.0001	0.0001
Previous Stillbirth	Chronic Kidney Disease	0.0513	0.0513
Latest Preterm birth	Hypertension	< 0.0001	< 0.0001
Latest Preterm birth	Type 2 diabetes	0.0004	0.0006
Latest Preterm birth	Cardiovascular disease	< 0.0001	< 0.0001
Latest Preterm birth	Chronic Kidney Disease	0.9264	0.9264
Previous Preterm birth	Hypertension	< 0.0001	< 0.0001
Previous Preterm birth	Type 2 diabetes	< 0.0001	< 0.0001
Previous Preterm birth	Cardiovascular disease	0.0081	0.0081
Previous Preterm birth	Chronic Kidney Disease	< 0.0001	0.0001
Latest SGA	Hypertension	0.5629	0.5629
Latest SGA	Type 2 diabetes	< 0.0001	< 0.0001
Latest SGA	Cardiovascular disease	0.0083	0.0110
Latest SGA	Chronic Kidney Disease	0.1308	0.1308
Previous SGA	Hypertension	< 0.0001	< 0.0001
Previous SGA	Type 2 diabetes	0.0819	0.1639

Previous SGA	Cardiovascular disease	0.7713	0.9410
Previous SGA	Chronic Kidney Disease	0.9410	0.9410
Latest Postnatal depression	Hypertension	< 0.0001	< 0.0001
Latest Postnatal depression	Type 2 diabetes	< 0.0001	< 0.0001
Latest Postnatal depression	Cardiovascular disease	< 0.0001	< 0.0001
Latest Postnatal depression	Chronic Kidney Disease	< 0.0001	< 0.0001
Previous Postnatal depression	Hypertension	< 0.0001	< 0.0001
Previous Postnatal depression	Type 2 diabetes	< 0.0001	< 0.0001
Previous Postnatal depression	Cardiovascular disease	< 0.0001	< 0.0001
Previous Postnatal depression	Chronic Kidney Disease	0.0003	0.0003

Results of multiple testing adjustment applied to primary analyses assessing associations between pregnancy complications and cardiometabolic outcomes. Adjusted p-values are presented to account for the number of statistical tests performed. **Abbreviations:** CVD, cardiovascular disease; CKD, chronic kidney disease; T2DM, type 2 diabetes mellitus. Multiple testing adjustment was performed using Benjamini-Hochberg method of false discovery rate (FDR).

Sensitivity analyses

Table 9: Comparison of cohort size, outcome counts and median follow-up between the primary (latest-pregnancy) analysis and the random- and first-pregnancy sensitivity analyses.

Outcome cohort	Index-pregnancy definition	n	Outcomes during follow-up, n (%)	Median follow-up, years (IQR)
**Hypertension**	Latest pregnancy (primary analysis)	1,389,460	49,233 (3.5)	4.11 (1.46 to 9.36)
	Random pregnancy	1,489,094	54,776 (3.7)	4.88 (1.79 to 10.52)
	First pregnancy	1,568,537	59,763 (3.8)	5.63 (2.12 to 11.67)
**T2DM**	Latest pregnancy (primary analysis)	1,411,883	21,754 (1.5)	4.21 (1.49 to 9.65)
	Random pregnancy	1,509,387	24,354 (1.6)	4.99 (1.82 to 10.82)
	First pregnancy	1,586,728	26,512 (1.7)	5.76 (2.15 to 11.97)
**CVD**	Latest pregnancy (primary analysis)	1,422,287	16,069 (1.1)	4.24 (1.49 to 9.69)
	Random pregnancy	1,519,305	17,459 (1.1)	5.02 (1.82 to 10.87)
	First pregnancy	1,596,025	18,636 (1.2)	5.80 (2.16 to 12.04)
**CKD**	Latest pregnancy (primary analysis)	1,430,965	5,288 (0.4)	4.25 (1.49 to 9.75)
	Random pregnancy	1,527,623	5,619 (0.4)	5.04 (1.83 to 10.93)
	First pregnancy	1,603,984	5,914 (0.4)	5.83 (2.17 to 12.10)

IQR, interquartile range.

For each of the four cardiometabolic-renal outcome cohorts, the table shows the number of eligible women (n), the number and percentage who developed the outcome during follow-up, and the median follow-up time with interquartile range (IQR), under each index-pregnancy definition. The primary analysis used the latest pregnancy as the index date; the two sensitivity analyses used a randomly selected pregnancy and the first recorded pregnancy, respectively. n denotes the number of women remaining after exclusion of those with a record of the outcome at or before the index date.

Pregnancy complications and long-term maternal cardiometabolic outcomes: adjusted estimates from primary (last-pregnancy) and sensitivity (random- and first-pregnancy) analyses

Figure 8a: Risk of hypertension

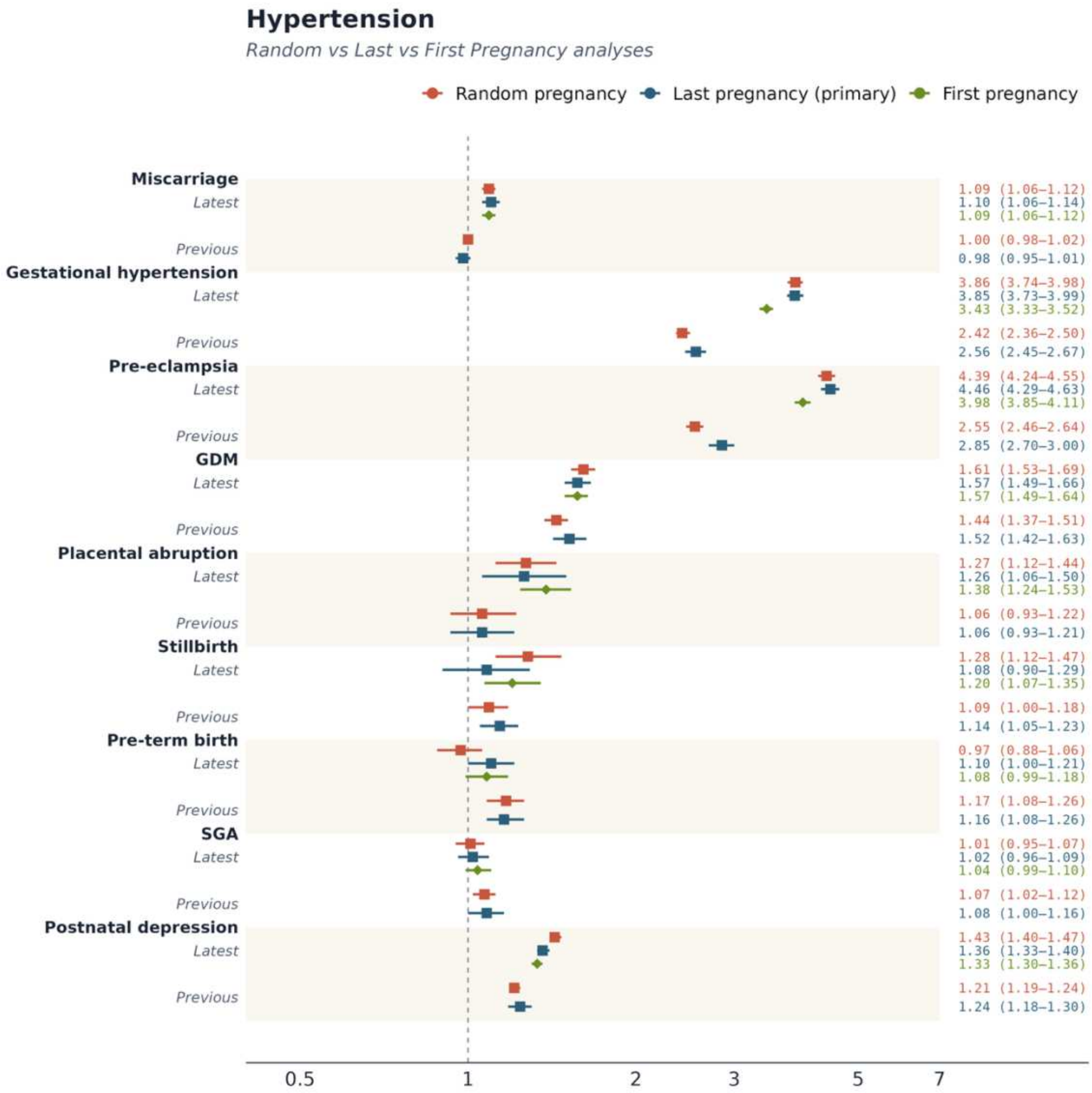

Figure 8b: Risk of T2DM

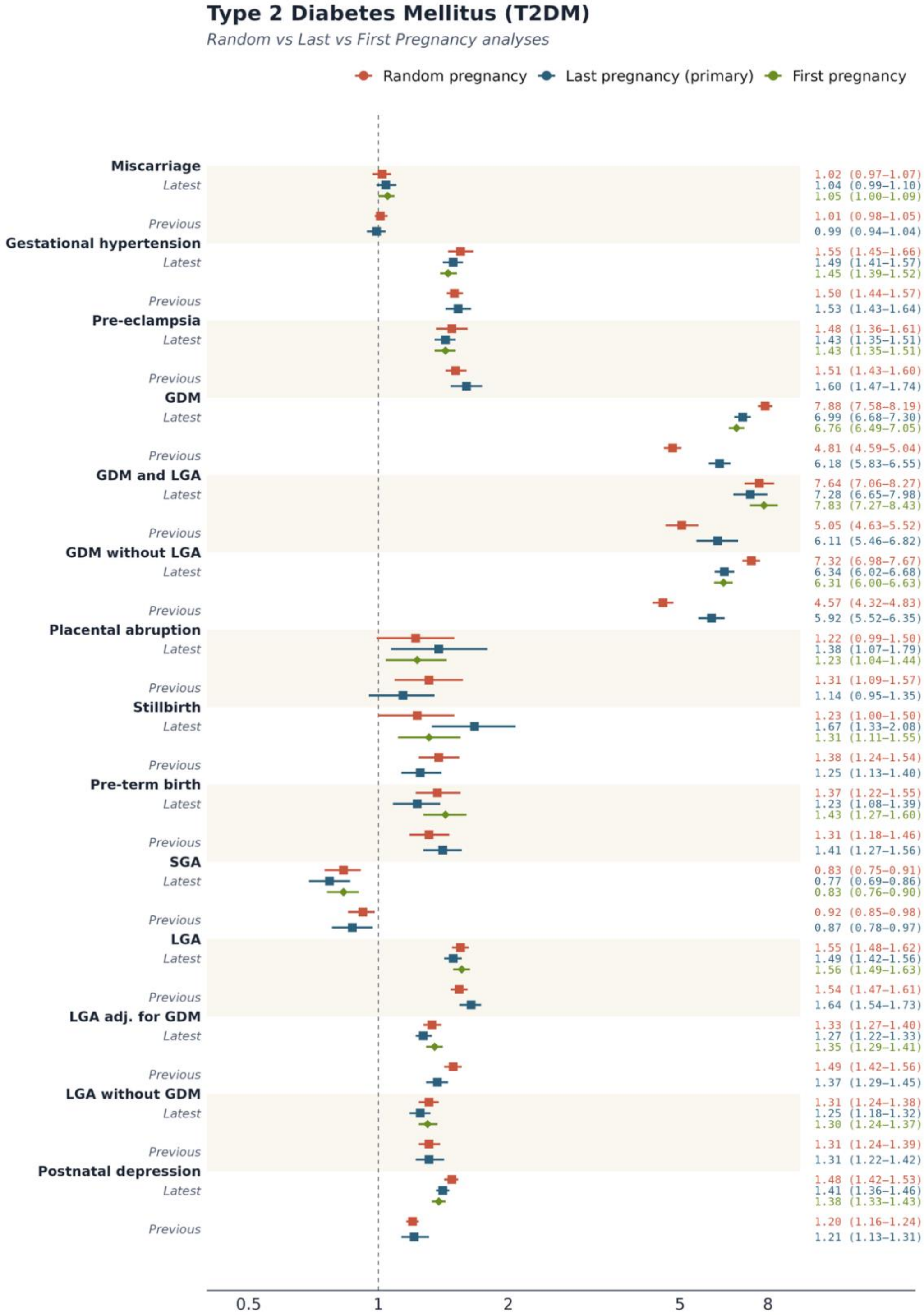

Figure 8c: Risk of CVD

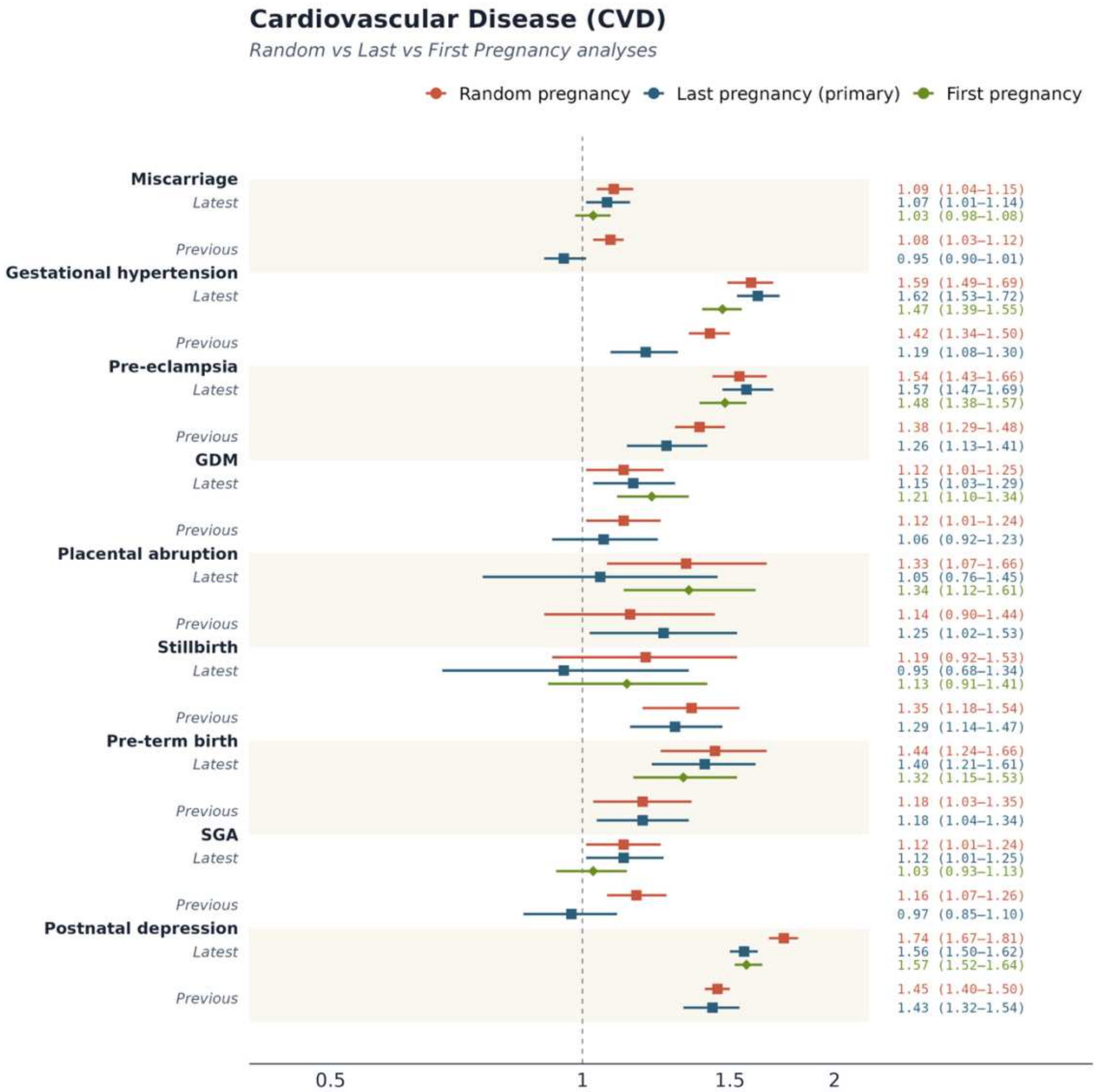

Figure 8d: Risk of CKD

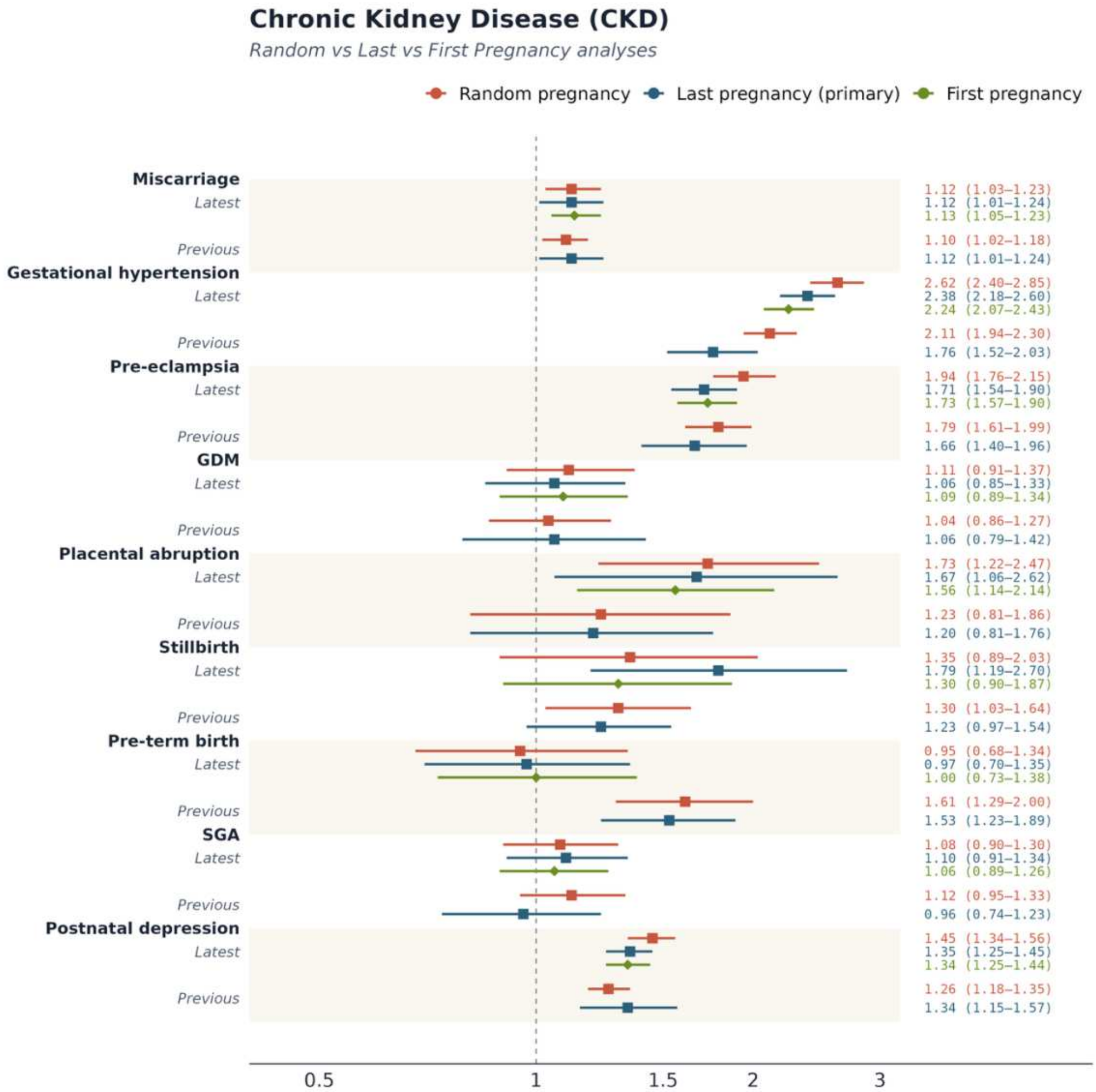

Proportion of women with a recorded SBP, HbA1c and eGFR measurement, and number of consultations, within 1 year of the index date, by exposure status

Table 10: Hypertension cohort

Exposure	Status	N	SBP recorded, n (%)	HbA1c recorded, n (%)	eGFR recorded, n (%)	Consultations, median (IQR)
**Overall cohort**		1,389,460	496,909 (35.8)	99,100 (7.1)	234,584 (16.9)	3 (1–7)
GDM, gestational hypertension and pre-eclampsia						
**Gestational diabetes**	None	1,326,516	470,697 (35.5)	80,076 (6.0)	213,446 (16.1)	3 (1–7)
	Current	44,332	19,664 (44.4)	14,150 (31.9)	15,806 (35.7)	5 (2–11)
	Previous	18,612	6,548 (35.2)	4,874 (26.2)	5,332 (28.6)	4 (1–9)
**Gestational hypertension**	None	1,327,774	471,354 (35.5)	93,442 (7.0)	222,133 (16.7)	3 (1–7)
	Current	34,407	14,696 (42.7)	2,909 (8.5)	6,939 (20.2)	4 (1–8)
	Previous	27,279	10,859 (39.8)	2,749 (10.1)	5,512 (20.2)	4 (1–8)
**Pre-eclampsia**	None	1,351,129	480,495 (35.6)	95,191 (7.0)	226,191 (16.7)	3 (1–7)
	Current	22,098	9,858 (44.6)	2,113 (9.6)	4,899 (22.2)	4 (1–8)
	Previous	16,233	6,556 (40.4)	1,796 (11.1)	3,494 (21.5)	4 (1–8)
Other pregnancy complications						
**Small for gestational age**	None	1,334,625	476,925 (35.7)	95,210 (7.1)	225,186 (16.9)	3 (1–7)
	Current	33,624	12,276 (36.5)	1,813 (5.4)	5,256 (15.6)	3 (1–7)
	Previous	21,211	7,708 (36.3)	2,077 (9.8)	4,142 (19.5)	4 (1–8)
**Postnatal depression**	None	1,163,203	404,167 (34.7)	73,466 (6.3)	177,389 (15.3)	3 (1–6)
	Current	181,526	75,181 (41.4)	20,859 (11.5)	46,635 (25.7)	6 (2–12)
	Previous	44,731	17,561 (39.3)	4,775 (10.7)	10,560 (23.6)	5 (2–10)
**Miscarriage**	None	1,188,362	425,536 (35.8)	80,749 (6.8)	195,399 (16.4)	3 (1–7)
	Current	76,565	26,847 (35.1)	6,887 (9.0)	15,437 (20.2)	4 (1–8)
	Previous	124,533	44,526 (35.8)	11,464 (9.2)	23,748 (19.1)	4 (1–8)
**Preterm birth**	None	1,342,122	479,754 (35.7)	93,830 (7.0)	225,371 (16.8)	3 (1–7)
	Current	17,244	6,314 (36.6)	1,897 (11.0)	3,453 (20.0)	4 (1–8)
	Previous	24,809	8,773 (35.4)	3,124 (12.6)	5,004 (20.2)	4 (1–9)
**Stillbirth**	None	1,375,223	491,887 (35.8)	97,666 (7.1)	231,756 (16.9)	3 (1–7)
	Current	2,049	679 (33.1)	184 (9.0)	372 (18.2)	3 (1–9)
	Previous	11,118	3,956 (35.6)	1,210 (10.9)	2,308 (20.8)	4 (1–8)
**Placental abruption**	None	1,380,102	493,501 (35.8)	98,332 (7.1)	232,814 (16.9)	3 (1–7)
	Current	3,942	1,445 (36.7)	246 (6.2)	662 (16.8)	3 (1–8)
	Previous	5,416	1,963 (36.2)	522 (9.6)	1,108 (20.5)	4 (1–8)

Table 11: T2DM cohort

Exposure	Status	N	SBP recorded, n (%)	HbA1c recorded, n (%)	eGFR recorded, n (%)	Consultations, median (IQR)
**Overall cohort**		1,411,883	511,078 (36.2)	97,199 (6.9)	240,485 (17.0)	3 (1–7)
GDM, gestational hypertension and pre-eclampsia						
**Gestational diabetes**	None	1,358,544	492,236 (36.2)	83,958 (6.2)	226,306 (16.7)	3 (1–7)
	Current	34,713	12,268 (35.3)	8,495 (24.5)	8,940 (25.8)	4 (1–9)
	Previous	18,626	6,574 (35.3)	4,746 (25.5)	5,239 (28.1)	4 (1–9)
**Gestational hypertension**	None	1,332,890	473,217 (35.5)	89,030 (6.7)	220,632 (16.6)	3 (1–7)
	Current	48,183	24,942 (51.8)	5,090 (10.6)	13,175 (27.3)	4 (1–9)
	Previous	30,810	12,919 (41.9)	3,079 (10.0)	6,678 (21.7)	4 (1–8)
**Pre-eclampsia**	None	1,358,997	483,816 (35.6)	91,021 (6.7)	225,499 (16.6)	3 (1–7)
	Current	34,142	19,140 (56.1)	4,146 (12.1)	10,601 (31.0)	5 (1–9)
	Previous	18,744	8,122 (43.3)	2,032 (10.8)	4,385 (23.4)	4 (1–8)
Other pregnancy complications						
**Small for gestational age**	None	1,355,544	490,070 (36.2)	93,243 (6.9)	230,596 (17.0)	3 (1–7)
	Current	34,458	12,856 (37.3)	1,829 (5.3)	5,510 (16.0)	3 (1–7)
	Previous	21,881	8,152 (37.3)	2,127 (9.7)	4,379 (20.0)	4 (1–8)
**Postnatal depression**	None	1,180,321	414,898 (35.2)	71,779 (6.1)	181,369 (15.4)	3 (1–6)
	Current	186,007	78,119 (42.0)	20,695 (11.1)	48,280 (26.0)	6 (2–12)
	Previous	45,555	18,061 (39.6)	4,725 (10.4)	10,836 (23.8)	5 (2–10)
**Miscarriage**	None	1,206,838	437,215 (36.2)	79,173 (6.6)	200,174 (16.6)	3 (1–7)
	Current	78,220	27,966 (35.8)	6,759 (8.6)	15,970 (20.4)	4 (1–8)
	Previous	126,825	45,897 (36.2)	11,267 (8.9)	24,341 (19.2)	4 (1–8)
**Preterm birth**	None	1,363,953	493,585 (36.2)	92,130 (6.8)	231,207 (17.0)	3 (1–7)
	Current	17,444	6,422 (36.8)	1,789 (10.3)	3,470 (19.9)	4 (1–8)
	Previous	25,207	8,998 (35.7)	3,066 (12.2)	5,079 (20.1)	4 (1–9)
**Stillbirth**	None	1,397,320	505,875 (36.2)	95,878 (6.9)	237,624 (17.0)	3 (1–7)
	Current	2,097	697 (33.2)	164 (7.8)	378 (18.0)	3 (1–9)
	Previous	11,402	4,125 (36.2)	1,130 (9.9)	2,346 (20.6)	4 (1–8)
**Placental abruption**	None	1,402,208	507,472 (36.2)	96,413 (6.9)	238,606 (17.0)	3 (1–7)
	Current	4,039	1,515 (37.5)	244 (6.0)	695 (17.2)	3 (1–8)
	Previous	5,636	2,091 (37.1)	542 (9.6)	1,184 (21.0)	4 (1–8.25)
**Large for gestational age**	None	1,215,416	438,933 (36.1)	83,393 (6.9)	207,608 (17.1)	3 (1–7)
	Current	144,807	53,592 (37.0)	8,431 (5.8)	22,424 (15.5)	3 (1–7)
	Previous	51,660	18,553 (35.9)	5,375 (10.4)	10,453 (20.2)	4 (1–8)

Table 12: CVD cohort

Exposure	Status	N	SBP recorded, n (%)	HbA1c recorded, n (%)	eGFR recorded, n (%)	Consultations, median (IQR)
**Overall cohort**		1,422,287	521,198 (36.6)	106,245 (7.5)	250,696 (17.6)	3 (1–7)
GDM, gestational hypertension and pre-eclampsia						
**Gestational diabetes**	None	1,353,217	490,133 (36.2)	84,161 (6.2)	225,459 (16.7)	3 (1–7)
	Current	49,336	23,751 (48.1)	16,796 (34.0)	19,341 (39.2)	6 (2–11)
	Previous	19,734	7,314 (37.1)	5,288 (26.8)	5,896 (29.9)	5 (1–9)
**Gestational hypertension**	None	1,340,875	481,178 (35.9)	96,261 (7.2)	228,809 (17.1)	3 (1–7)
	Current	49,938	26,542 (53.1)	6,392 (12.8)	14,646 (29.3)	4 (1–9)
	Previous	31,474	13,478 (42.8)	3,592 (11.4)	7,241 (23.0)	4 (1–8)
**Pre-eclampsia**	None	1,367,284	492,046 (36.0)	98,502 (7.2)	233,942 (17.1)	3 (1–7)
	Current	35,747	20,599 (57.6)	5,324 (14.9)	11,938 (33.4)	5 (2–10)
	Previous	19,256	8,553 (44.4)	2,419 (12.6)	4,816 (25.0)	4 (1–9)
Other pregnancy complications						
**Small for gestational age**	None	1,365,831	500,010 (36.6)	102,052 (7.5)	240,603 (17.6)	3 (1–7)
	Current	34,488	12,924 (37.5)	1,927 (5.6)	5,600 (16.2)	3 (1–7)
	Previous	21,968	8,264 (37.6)	2,266 (10.3)	4,493 (20.5)	4 (1–8)
**Postnatal depression**	None	1,188,078	422,439 (35.6)	78,321 (6.6)	188,976 (15.9)	3 (1–6)
	Current	188,191	80,264 (42.7)	22,759 (12.1)	50,436 (26.8)	6 (2–12)
	Previous	46,018	18,495 (40.2)	5,165 (11.2)	11,284 (24.5)	5 (2–10)
**Miscarriage**	None	1,215,276	445,459 (36.7)	86,438 (7.1)	208,523 (17.2)	3 (1–7)
	Current	79,095	28,803 (36.4)	7,510 (9.5)	16,757 (21.2)	4 (1–8)
	Previous	127,916	46,936 (36.7)	12,297 (9.6)	25,416 (19.9)	4 (1–8)
**Preterm birth**	None	1,373,643	503,121 (36.6)	100,587 (7.3)	240,842 (17.5)	3 (1–7)
	Current	17,713	6,655 (37.6)	2,020 (11.4)	3,689 (20.8)	4 (1–8)
	Previous	25,566	9,291 (36.3)	3,373 (13.2)	5,380 (21.0)	4 (1–9)
**Stillbirth**	None	1,407,269	515,626 (36.6)	104,629 (7.4)	247,509 (17.6)	3 (1–7)
	Current	2,151	745 (34.6)	201 (9.3)	416 (19.3)	4 (1–9)
	Previous	11,780	4,429 (37.6)	1,375 (11.7)	2,621 (22.2)	4 (1–8)
**Placental abruption**	None	1,412,520	517,520 (36.6)	105,396 (7.5)	248,749 (17.6)	3 (1–7)
	Current	4,080	1,544 (37.8)	263 (6.4)	721 (17.7)	3 (1–8)
	Previous	5,687	2,134 (37.5)	586 (10.3)	1,226 (21.6)	4 (1–9)

Table 13: CKD cohort

Exposure	Status	N	SBP recorded, n (%)	HbA1c recorded, n (%)	eGFR recorded, n (%)	Consultations, median (IQR)
**Overall cohort**		1,430,965	525,311 (36.7)	107,549 (7.5)	253,334 (17.7)	3 (1–7)
GDM, gestational hypertension and pre-eclampsia						
**Gestational diabetes**	None	1,361,182	493,778 (36.3)	85,148 (6.3)	227,679 (16.7)	3 (1–7)
	Current	49,902	24,141 (48.4)	17,048 (34.2)	19,679 (39.4)	6 (2–11)
	Previous	19,881	7,392 (37.2)	5,353 (26.9)	5,976 (30.1)	5 (1–10)
**Gestational hypertension**	None	1,348,849	484,899 (35.9)	97,397 (7.2)	231,191 (17.1)	3 (1–7)
	Current	50,381	26,789 (53.2)	6,500 (12.9)	14,803 (29.4)	4 (1–9)
	Previous	31,735	13,623 (42.9)	3,652 (11.5)	7,340 (23.1)	4 (1–8)
**Pre-eclampsia**	None	1,375,497	495,897 (36.1)	99,680 (7.2)	236,423 (17.2)	3 (1–7)
	Current	36,060	20,776 (57.6)	5,414 (15.0)	12,046 (33.4)	5 (2–10)
	Previous	19,408	8,638 (44.5)	2,455 (12.6)	4,865 (25.1)	4 (1–9)
Other pregnancy complications						
**Small for gestational age**	None	1,374,160	503,983 (36.7)	103,316 (7.5)	243,145 (17.7)	3 (1–7)
	Current	34,676	13,003 (37.5)	1,946 (5.6)	5,645 (16.3)	3 (1–7)
	Previous	22,129	8,325 (37.6)	2,287 (10.3)	4,544 (20.5)	4 (1–8)
**Postnatal depression**	None	1,194,076	425,074 (35.6)	79,016 (6.6)	190,481 (16.0)	3 (1–6)
	Current	190,445	81,529 (42.8)	23,298 (12.2)	51,425 (27.0)	6 (2–12)
	Previous	46,444	18,708 (40.3)	5,235 (11.3)	11,428 (24.6)	5 (2–10)
**Miscarriage**	None	1,222,489	448,923 (36.7)	87,504 (7.2)	210,706 (17.2)	3 (1–7)
	Current	79,684	29,058 (36.5)	7,606 (9.5)	16,963 (21.3)	4 (1–8)
	Previous	128,792	47,330 (36.7)	12,439 (9.7)	25,665 (19.9)	4 (1–8)
**Preterm birth**	None	1,381,965	507,088 (36.7)	101,818 (7.4)	243,389 (17.6)	3 (1–7)
	Current	17,817	6,693 (37.6)	2,038 (11.4)	3,710 (20.8)	4 (1–8)
	Previous	25,795	9,391 (36.4)	3,424 (13.3)	5,445 (21.1)	4 (1–9)
**Stillbirth**	None	1,415,805	519,662 (36.7)	105,898 (7.5)	250,083 (17.7)	3 (1–7)
	Current	2,169	754 (34.8)	204 (9.4)	427 (19.7)	4 (1–9)
	Previous	11,891	4,489 (37.8)	1,404 (11.8)	2,667 (22.4)	4 (1–8)
**Placental abruption**	None	1,421,121	521,586 (36.7)	106,682 (7.5)	251,356 (17.7)	3 (1–7)
	Current	4,103	1,557 (37.9)	266 (6.5)	728 (17.7)	3 (1–8)
	Previous	5,741	2,168 (37.8)	601 (10.5)	1,250 (21.8)	4 (1–9)
