# Supplementary material for: Pregnancy complications and risk of cardiometabolic-renal conditions in 1.4 million women in England: longitudinal study
Source: BMJ Med. 2026 Oct 6;5(1):e003035. doi: 10.1136/bmjmed-2026-003035 (PMC13647177; doi:10.1136/bmjmed-2026-003035)
Supplement: Supplementary file 3 [file bmjmed-5-1-s003.pdf]

Data for forest plots in the main manuscript

Figure 4: Adjusted hazard ratios for the association between pregnancy complications and hypertension

Pregnancy complication	Pregnancy history	Adjusted HR (95% CI)
Miscarriage	Latest	1.10 (1.06 to 1.14)
Miscarriage	Previous	0.98 (0.95 to 1.01)
Gestational hypertension	Latest	3.85 (3.73 to 3.99)
Gestational hypertension	Previous	2.56 (2.45 to 2.67)
Pre-eclampsia	Latest	4.46 (4.29 to 4.63)
Pre-eclampsia	Previous	2.85 (2.70 to 3.00)
GDM	Latest	1.57 (1.49 to 1.66)
GDM	Previous	1.52 (1.42 to 1.63)
Placental abruption	Latest	1.26 (1.06 to 1.50)
Placental abruption	Previous	1.06 (0.93 to 1.21)
Stillbirth	Latest	1.08 (0.90 to 1.29)
Stillbirth	Previous	1.14 (1.05 to 1.23)
Preterm birth	Latest	1.10 (1.00 to 1.21)
Preterm birth	Previous	1.16 (1.08 to 1.26)
SGA	Latest	1.02 (0.96 to 1.09)
SGA	Previous	1.08 (1.00 to 1.16)
Postnatal depression	Latest	1.36 (1.33 to 1.40)
Postnatal depression	Previous	1.24 (1.18 to 1.30)

Figure 6: Adjusted hazard ratios for the association between pregnancy complications and type 2 diabetes mellitus

Pregnancy complication	Pregnancy history	Adjusted HR (95% CI)
Miscarriage	Latest	1.04 (0.99 to 1.10)
Miscarriage	Previous	0.99 (0.94 to 1.04)
Gestational hypertension	Latest	1.49 (1.41 to 1.57)
Gestational hypertension	Previous	1.53 (1.43 to 1.64)
Pre-eclampsia	Latest	1.43 (1.35 to 1.51)
Pre-eclampsia	Previous	1.60 (1.47 to 1.74)
GDM	Latest	6.99 (6.68 to 7.30)
GDM	Previous	6.18 (5.83 to 6.55)
GDM and LGA	Latest	7.28 (6.65 to 7.98)
GDM and LGA	Previous	6.11 (5.46 to 6.82)
GDM without LGA	Latest	6.34 (6.02 to 6.68)
GDM without LGA	Previous	5.92 (5.52 to 6.35)
Placental abruption	Latest	1.38 (1.07 to 1.79)
Placental abruption	Previous	1.14 (0.95 to 1.35)
Stillbirth	Latest	1.67 (1.33 to 2.08)
Stillbirth	Previous	1.25 (1.13 to 1.40)
Preterm birth	Latest	1.23 (1.08 to 1.39)
Preterm birth	Previous	1.41 (1.27 to 1.56)
SGA	Latest	0.77 (0.69 to 0.86)
SGA	Previous	0.87 (0.78 to 0.97)
LGA	Latest	1.49 (1.42 to 1.56)
LGA	Previous	1.64 (1.54 to 1.73)
LGA adjusting for GDM	Latest	1.27 (1.22 to 1.33)
LGA adjusting for GDM	Previous	1.37 (1.29 to 1.45)
LGA without GDM	Latest	1.25 (1.18 to 1.32)
LGA without GDM	Previous	1.31 (1.22 to 1.42)
Postnatal depression	Latest	1.41 (1.36 to 1.46)
Postnatal depression	Previous	1.21 (1.13 to 1.31)

Figure 8: Adjusted hazard ratios for the association between pregnancy complications and cardiovascular disease

Pregnancy complication	Pregnancy history	Adjusted HR (95% CI)
Miscarriage	Latest	1.07 (1.01 to 1.14)
Miscarriage	Previous	0.95 (0.90 to 1.01)
Gestational hypertension	Latest	1.62 (1.53 to 1.72)
Gestational hypertension	Previous	1.19 (1.08 to 1.30)
Pre-eclampsia	Latest	1.57 (1.47 to 1.69)
Pre-eclampsia	Previous	1.26 (1.13 to 1.41)
GDM	Latest	1.15 (1.03 to 1.29)
GDM	Previous	1.06 (0.92 to 1.23)
Placental abruption	Latest	1.05 (0.76 to 1.45)
Placental abruption	Previous	1.25 (1.02 to 1.53)
Stillbirth	Latest	0.95 (0.68 to 1.34)
Stillbirth	Previous	1.29 (1.14 to 1.47)
Preterm birth	Latest	1.40 (1.21 to 1.61)
Preterm birth	Previous	1.18 (1.04 to 1.34)
SGA	Latest	1.12 (1.01 to 1.25)
SGA	Previous	0.97 (0.85 to 1.10)
Postnatal depression	Latest	1.56 (1.50 to 1.62)
Postnatal depression	Previous	1.43 (1.32 to 1.54)

Figure 10: Adjusted hazard ratios for the association between pregnancy complications and chronic kidney disease

Pregnancy complication	Pregnancy history	Adjusted HR (95% CI)
Miscarriage	Latest	1.12 (1.01 to 1.24)
Miscarriage	Previous	1.12 (1.01 to 1.24)
Gestational hypertension	Latest	2.38 (2.18 to 2.60)
Gestational hypertension	Previous	1.76 (1.52 to 2.03)
Pre-eclampsia	Latest	1.71 (1.54 to 1.90)
Pre-eclampsia	Previous	1.66 (1.40 to 1.96)
GDM	Latest	1.06 (0.85 to 1.33)
GDM	Previous	1.06 (0.79 to 1.42)
Placental abruption	Latest	1.67 (1.06 to 2.62)
Placental abruption	Previous	1.20 (0.81 to 1.76)
Stillbirth	Latest	1.79 (1.19 to 2.70)
Stillbirth	Previous	1.23 (0.97 to 1.54)
Preterm birth	Latest	0.97 (0.70 to 1.35)
Preterm birth	Previous	1.53 (1.23 to 1.89)
SGA	Latest	1.10 (0.91 to 1.34)
SGA	Previous	0.96 (0.74 to 1.23)
Postnatal depression	Latest	1.35 (1.25 to 1.45)
Postnatal depression	Previous	1.34 (1.15 to 1.57)
